# Human *PRH1*, *PRH2* susceptibility and resistance and *Streptococcus mutans* virulence phenotypes specify different microbial profiles in caries

**DOI:** 10.1016/j.ebiom.2024.105001

**Published:** 2024-02-15

**Authors:** Nongfei Sheng, Lena Mårell, Raviprakash Tumkur Sitaram, Gunnel Svensäter, Anna Westerlund, Nicklas Strömberg

**Affiliations:** a Department of Odontology/Cariology, Umeå University, 901 87, Umeå, Sweden; b Faculty of Odontology, Malmö University, 205 06, Malmö, Sweden; c Department of Orthodontics, Sahlgrenska Academy, University of Gothenburg, 413 90, Göteborg, Sweden

**Keywords:** *Streptococcus mutans*, Host susceptibility, *PRH1/PRH2*, Caries, Commensal pathogen, Adhesion

## Abstract

**Background:**

Lifestyle- and sucrose-dependent polymicrobial ecological shifts are a primary cause of caries in populations with high caries prevalence. In populations with low prevalence, *PRH1*, *PRH2* susceptibility and resistance phenotypes may interact with the *Streptococcus mutans* adhesin cariogenicity phenotype to affect caries progression, but studies are lacking on how these factors affect the microbial profile of caries.

**Methods:**

We analysed how the residency and infection profiles of *S. mutans* adhesin (SpaP A/B/C and Cnm/Cbm) phenotypes and commensal streptococci and lactobacilli influenced caries progression in a prospective case–referent sample of 452 Swedish adolescents with high (P4a), moderate (P6), and low (P1) caries *PRH1*, *PRH2* phenotypes. Isolates of *S. mutans* from participants were analysed for adhesin expression and glycosylation and *in vitro* and *in situ* mechanisms related to caries activity.

**Findings:**

Among adolescents with the resistant (P1) phenotype, infection with *S. mutans* high-virulence phenotypes was required for caries progression. In contrast, with highly (P4a) or moderately (P6) susceptible phenotypes, caries developed from a broader polymicrobial flora that included moderately cariogenic oral commensal streptococci and lactobacilli and *S. mutans* phenotypes. High virulence involved unstable residency and fluctuating SpaP ABC, B-1, or Cnm expression/glycosylation phenotypes, whereas low/moderate virulence involved SpaP A phenotypes with stable residency. Adhesin phenotypes did not display changes in individual host residency but were paired within individuals and geographic regions.

**Interpretation:**

These results suggest that receptor *PRH1*, *PRH2* susceptibility and resistance and *S. mutans* adhesin virulence phenotypes specify different microbial profiles in caries.

**Funding:**

10.13039/501100004359Swedish Research Council and funding bodies listed in the acknowledgement section.

**Research in context**

**Evidence before this study**

The causes of dental caries have been studied extensively because improved risk prediction and therapeutic tools are needed. Lifestyle- and sucrose-dependent polymicrobial ecological shifts and mechanisms are primary causes of caries in populations with high caries prevalence and physiological (lifestyle) heterogeneity. However, in populations with low caries prevalence and lifestyle homogeneity, the influence of human genetic (*PRH1*, *PRH2*) susceptibility and resistance and *S. mutans* moderate-to high-cariogenicity phenotypes remains to be studied.

**Added value of this study**

Human genetic *PRH1*, *PRH2* susceptibility and resistance phenotypes may dictate both the repertoire of and sensitivity to the microbiota colonising teeth in low-caries populations. In a population with low caries prevalence in Sweden, caries progression in individuals with resistance was linked to high-virulence phenotypes of *S. mutans*, whereas individuals with high or moderate susceptibility had a broader commensal and moderate cariogenic flora. High-virulence *S. mutans* phenotypes had unstable residency and mixed fluctuating SpaP ABC, B-1, and Cnm expression/glycosylation phenotypes, whereas moderately cariogenic SpaP A phenotypes showed stable persistence and high prevalence. The specificity (tropism) of *S. mutans* for individual hosts and the narrow distribution of high-virulence phenotypes in a few individuals emphasise the influence of the host genome on the microbiota and the unique character of each host–microbe interaction.

**Implications of all the available evidence**

Our findings emphasise that the influence of the host genome on the microbiota and innate defences may play a causal role in caries progression in populations with low caries prevalence and physiological (lifestyle) homogeneity. Furthermore, biomarker classification models of low, moderate, and high risk should be designed to fit the target population in terms of caries prevalence/physiological homogeneity. Fine-tuned personalised risk assessment models will require detailed information about the unique character of each host–microbe and host–exposome interaction.

## Introduction

Dental caries is a common, costly, and chronic infectious disease.^1^, ^2^, ^3^ In populations with a high prevalence of caries and physiological heterogeneity in terms of poor diet and oral hygiene, it is considered a lifestyle condition.^1^, ^2^, ^3^ In Sweden and other Western countries, however, prevention and other advances combined with lifestyle homogeneity have led to populations with a low caries prevalence.^4^, ^5^, ^6^ Accordingly, a large proportion of these populations (∼85%) is virtually free of caries, even as a high-risk subset (∼15%) experiences recurrent caries.^4^^,^^5^^,^^7^ These low- and high-risk cases manifest stable trajectories in the primary and permanent dentitions.^4^^,^^5^^,^^7^ In this context, human genetic diversity takes on an increasing causal role in populations with decreasing caries prevalence.

Twin^8^ and human experimental^9^ studies implicated genetics in caries early on but led to no practical applications in populations with a high caries prevalence driven by poor diet and oral hygiene. Adding to limits on clinical utility, a conservative interpretation is that caries results from a multigenic predisposition involving myriad genetic and other polymorphisms that each confer a modest relative risk. A third factor limiting clinical application is that evidence has been lacking for specific genes or alleles that predict caries progression. Accordingly, genome-wide association studies^10^ and targeted genetic^11^^,^^12^ studies with prospective designs may be fruitful for identifying high-risk individuals in low-caries populations.^13^, ^14^, ^15^

Among the genetic candidates are the *PRH1* and *PRH2* genes, which encode salivary acidic proline-rich proteins (PRPs). These two genes are represented by five major allelic variants [PIF, Pa, Db (*PRH1*), and PRP-1 and PRP-2 (*PRH2*)], which lead to three common phenotypes (P4a, P6, and P1) and up to 15 minor PRP phenotypes.^11^ PRPs are tooth surface receptors for sucrose-independent adhesion of oral pioneer streptococci and actinomycetes bacteria.^16^, ^17^, ^18^ These bacteria also adhere to each other to form the intra-generic *Streptococcus* group 1–6 and *Streptococcus*-*Actinomyces* group A–F biofilm communities.^16^, ^17^, ^18^ Moreover, the PRPs act as co-receptors for *S. mutans*,^16^ as a barrier against bacterial acids, and in taste perception and pattern recognition.^19^^,^^20^ The PRPs, which release bioactive peptides upon bacterial proteolysis,^21^ are secreted as large receptor active and small receptor inactive variants with increased affinity for the tooth surface. It is thus reasonable to hypothesise that *PRH1*, *PRH2*–related variation in PRP saliva composition may dictate the repertoire of and sensitivity to initial biofilm communities on teeth.

As we have shown in a prospective case–referent study of 452 adolescents in Sweden, human *PRH1*, *PRH2* allelic variation predicts caries progression in terms of high (P4a), moderate (P6), and low (P1) caries risk phenotypes and can be used to classify individuals into immunodeficient (P4a) and lifestyle (P1) causal subtypes of caries.^11^ In addition, in that study, caries progression with the *PRH1*, *PRH2* phenotypes P4a, P6, and P1 varied by infection status (+, –) with *S. mutans* and lactobacilli.^11^

The unspecific, specific, and ecological plaque (biofilm) hypotheses all relate to causal caries models developed in high caries populations.^1^^,^^22^, ^23^, ^24^ According to the unspecific hypothesis, all bacterial members and load cause caries, whereas the specific hypothesis predicts that the *S. mutans* pathogen drives caries. The ecological hypothesis predicts that caries is an ecological affliction in which plaque acidification selects for acid-tolerant and acid-producing bacteria, such as mutans and non-mutans streptococci and lactobacilli. Factors in all three hypotheses can synergise with lifestyle factors, such as poor oral hygiene and sugar intake, to affect bacteria load. Sucrose intake and associated mechanisms promote *S. mutans* biofilm formation, and poor oral hygiene with frequent sugar intake causes plaque acidification. The cariogenic mechanisms associated with sucrose are thus acid production and tolerance and sucrose-dependent adhesion and biofilm formation through glycosyltransferase activity. Support for the specific plaque hypothesis, which also may be viewed as implying a monomicrobial cause, has been hampered by the lack of evidence for *S. mutans* subtypes of low to high cariogenicity. In contrast, the ecological plaque hypothesis, which may viewed as implying a polymicrobial cause, has been supported by the identification of increasing numbers of caries-associated bacteria. However, no studies to date have been conducted in a low-caries population to evaluate the monomicrobial and polymicrobial profiles in caries or to test plaque hypotheses related to human genetic susceptibility and resistance and *S. mutans* virulence phenotypes.

Previously, we stratified *S. mutans* based on sucrose-independent adhesin (*spaP*/*cnm*/*cbm*) and housekeeping gene polymorphisms related to caries progression in our study population of adolescents.^12^ For that work, we distinguished *S. mutans* biotypes A, B, and C and corresponding core genome SpaP A/B/C (100%) and pan-genome Cnm (6%) and Cbm (1.5%) adhesin phenotypes of different cariogenic potential.^12^ Almost half of the adolescents who were infected (∼48%) harboured single dominant SpaP A/B/C types (94%), with SpaP B and Cnm phenotypes linked to high cariogenic potential and SpaP A showing comparably low potential.^12^^,^^25^ A few adolescents who were infected had mixed SpaP ABC (6%) types of unknown residency and cariogenicity. Biotypes A, B, and C differed in cariogenic mechanisms, such as in their adhesion and acid-tolerance properties.^12^

Saliva DMBT1 is a pattern-recognition receptor involved in caries.^26^^,^^27^ As we have shown, the binding avidity of the SpaP B and Cnm strains but not the SpaP A strain to DMBT1 correlates with caries activity in the strain donor.^12^ In that work, we also performed *spaP*-guided sequence typing of single dominant isolates from 35 extreme caries cases and 35 caries-free participants from among the 217 adolescents who were infected. We found that these patterns matched the identification of clonal complexes/lineages of different cariogenicity.^12^ For example, SpaP B-1 and SpaP A-1 showed markedly high and low *in vivo* cariogenic potential and *in vitro* adhesion and acid tolerance, respectively. SpaP^28^ is a member of the oral streptococcal AgI/II adhesin family. These adhesins mediate bacteria–bacteria adhesion, biofilm formation, and saliva adhesion and aggregation by DMBT1/salivary agglutinin.^29^^,^^30^

In addition to an association with caries, the Cnm and Cbm phenotypes of *S. mutans* are implicated as virulence factors in stroke, inflammatory bowel disease, and endocarditis.^22^^,^^31^^,^^32^ Each Cnm and Cbm adhesin binds to collagen and DMBT1 and harbours a collagen-binding domain and threonine-rich B-repeat for O-glycosylation. Cnm and Cbm are encoded by separate *cnm* and *cbm* operons with glycosyltransferase genes for O-glycosylation.^33^^,^^34^ O-glycosylation influences virulence through driving inhibition or avoidance of host immunity,^35^ but how Cnm/Cbm expression and glycosylation influence caries and virulence is not known.

We hypothesise that genetic variation in the human receptor and the *S. mutans* adhesin specify different microbial profiles in caries of populations with a low caries prevalence. The current study had three specific aims. We first sought to examine whether the *PRH1*, *PRH2* receptor phenotype dictates the repertoire of and sensitivity to the pioneer microbiota colonising teeth, predicting that resistant (P1) individuals develop caries largely from high-virulence *S. mutans* phenotypes and susceptible (P4a/P6) phenotypes from a broader microbial profile. Second, we tested whether the dominant *S. mutans* phenotypes have specificity (tropism) for individual hosts and show plausible family patterns. Our third and final aim was to further resolve each *S. mutans* SpaP A/B/C and Cnm/Cbm adhesin grouping in terms of low-to high-virulence phenotypes and mechanisms, residency, and distribution among individual hosts.

## Methods

### Precaries adolescence study

**Fig. 1:**
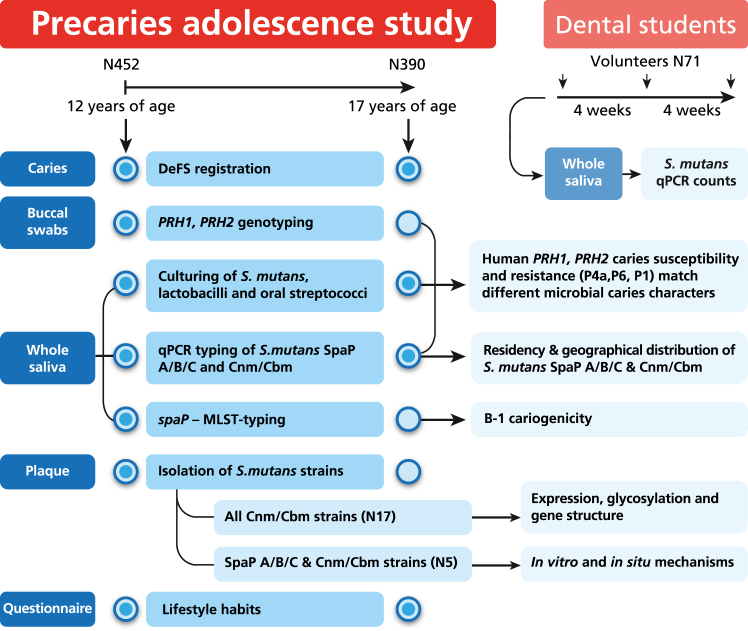
**Study design and overview**. The Precaries Adolescence Study case–referent design, data sampling, and analyses of participants at ages 12 and 17 years are outlined,^11^^,^^12^ with the data collected and analysed in the present paper marked in light blue. Also marked are the biological samples/data collected at ages 12 and 17 years (outer circle) and samples/data used at both ages (inner blue dots). The nested dental student study design is also outlined.

The adolescents included in this analysis participated in the Precaries Adolescence Study, a prospective case-referent study of 452 children sampled at ages 12 and 17 years at 13 public dental service clinics in the county of Västerbotten in northern Sweden (Fig. 1).^11^^,^^12^ The sample size of 452 cases–referents with a 14% dropout is justified by our previous studies, the 1.5:1 case–referent ratio, and established norms.^11^^,^^12^ Sex was self-reported by the participants and equally represented. The children underwent operative treatment and caries prevention, and were treated for orthodontic reasons if needed, according to the ordinary routines and policies at the clinics and in the county.^11^^,^^12^ Briefly, caries was recorded in each participant at ages 12 and 17 years.^11^ Three dentists conducted the assessments (intra- and inter-examiner kappa ≥0.98) using a mirror, probe, and two bitewing radiographs. The collection of clinical and biological samples and data, described in detail elsewhere, are outlined below (Fig. 1).^11^^,^^12^ The primary clinical outcome variables were decayed, enamel-included, filled surfaces (DeFS) at 12 and 17 years of age and ΔDeFS, calculated using the following equation:

$$\left(\frac{DeFS\;at\;17\;years\;-\;DeFS\;at\;12\;years}{number\;of\;days\;between\;two\;examinations}\times 365\times 5\right)$$

The 5-year change (ΔDeFS increment), a good estimate of incidence rate in this age group and population,^11^^,^^12^ is referred to in the text as caries progression or development. Buccal swabs, whole saliva, and plaque also were sampled from participants at ages 12 and 17 years.^11^^,^^12^ Whole saliva was collected by having the participant chew on paraffin, and plaque was sampled from buccal surfaces of teeth 34–36. Lifestyle and oral behaviour data were collected using questionnaires.^11^^,^^12^ The questions addressed oral hygiene (tooth brushing irregularly, once per day, twice per day, more than twice per day); intake frequency of sweets (*e.g*., cookies, biscuits, ice cream, dried fruit) and sugary drinks (never, once per month, once per week, several times per week, once per day, several times per day); and extra fluoride treatment in addition to fluoride in toothpaste (no = none, yes = fluoride mouth rinse, fluoride-containing chewing gum, or fluoride tablets).

The adolescents were categorised as having high (P4a), moderate (P6), and low (P1) caries phenotypes by *PRH1*, *PRH2* genotyping with an Illumina Golden Gate array (SNP&SEQ Technology Platform, Uppsala) and TaqMan typing, as previously described.^11^ Participants were typed for infection, *S. mutans* load *per se*, and *spaP A*/*B*/*C* and *cnm*/*cbm* adhesin types in whole saliva by qPCR, as has been described.^12^ Briefly, DNA was prepared from 400 μL of whole saliva. We used the GenElute Bacterial Genomic DNA Kit for DNA preparation (Sigma Aldrich, Sweden, NA2120). For qPCR typing of *S. mutans*, we used pure DNA from whole saliva, the KAPA SYBR FAST Universal qPCR kit (Sigma–Aldrich, Sweden, KK4602), and primers specific to the conserved *gtfB* and *gtfC* regions in a Corbett Rotor-Gene 6000 apparatus.^12^^,^^36^ Quantitative calibration curves were developed from DNA purified from serial dilutions of a *S. mutans* reference strain, and 3000 pg was set as the cut-off value for *S. mutans*–positive versus –negative saliva. For qPCR typing of the *spaP A*/*B*/*C* and *cnm*/*cbm* adhesion types, we used pure DNA from whole saliva, the KAPA SYBR Fast Universal qPCR kit (Sigma–Aldrich, Sweden, KK4602), and primers specific to each adhesin type in a Corbett Rotor gene 600 apparatus.^12^ The primers for *cnm* and *cbm* did not cross-react between or with other templates, and the primers for *spaP A*, *B*, and *C* were from the *spaP* sequences based on prior testing and lack of cross-reactivity among *A*, *B*, and *C* or with DNA from oral streptococci with *spaP* analogues. For typing, we used internal standards and quantitative calibration curves from dilutions of DNA purified from a reference genotype of each adhesin type and cut-off values for *A* (3000 pg), *B* (3000 pg), *C* (6000 pg), *cnm* (3000 pg), and *cbm* (1000 pg).

A total of 2500 strains of *S. mutans* were isolated from plaque on buccal surfaces 34–36 and on caries lesions by a foam pellet. Samples were cultured to homogeneity by repeated growth on selective agar plates and characterised by metabolic tests, as described previously.^12^ Typing of one isolate from each of the 214 participants with infection was done by qPCR for the adhesins SpaP A/B/C and Cnm/Cbm and serotype status, and was in good agreement with the results of saliva typing.^12^ The single isolates from 70 extreme cases (35 extreme caries and 35 caries-free cases) were also analysed by *spaP*-guided multi-locus sequence typing (MLST) and for cariogenic mechanisms, such as adhesion and acid tolerance.^11^^,^^12^ Additional isolates (2–8 isolates) from each individual showed the same sequence type by MLST in each case. A total of 12 *cnm*- and 5 *cbm*-positive strains were isolated.

Numbers of colony-forming units of *S. mutans*, oral streptococci, and lactobacilli in whole saliva or plaque suspension per millilitre were established by culturing on selective mitis salivarius-bacitracin, mitis salivarius, and Rogosa agar plates, respectively. The saliva and plaque suspensions in sterile M-DIL buffer (4.4 g NaCl, 0.42 g KCl, 1 g Na_2_HPO_4_, 1 g KH_2_PO_4_, 10 g sodium glycerophosphate, 0.1 g MgCl_2_ × 6H_2_O dissolved in 1000 mL water) were sonicated before culture. The plates were incubated at 37 °C for 2 days in a CO_2_ atmosphere for *S. mutans* and oral streptococci and at 37 °C for 2 days in an anaerobic atmosphere for lactobacilli.

### Genotyping of SpaP B-1 and non–B-1 subtypes

Individuals typed by qPCR as infected by *S. mutans* SpaP B were further assessed for B-1 or non–B-1 status by TaqMan typing of DNA from the dominant SpaP B isolate using the single nucleotide polymorphism (SNP) SpaP_3458_ (T defines the B-1 type and C the non–B-1).^12^ For the TaqMan assay, we used the TaqMan Genotyping Master Mix (Applied Biosystems, 4,371,355) and a predesigned PCR program consisting of an initial denaturing at 95 °C for 10 min, followed by 40 cycles of 92 °C for 15 s and 60 °C for 1 min. The SpaP_3458_ SNP and other canonical SNPs were generated from a reference set of 70 sequenced SpaP A/B/C and SpaP B-1 and non-B1 isolates using MEGA6 software,^37^ the Thermo Fisher custom genomic TaqMan assay, a design pipeline for non-human templates (https://www.thermofisher.com/order/custom-genomic-products/tools/genotyping), and the ABI PRISM 7900 HT system (Applied Biosystems). TaqMan typing of the 70 isolates from extreme caries cases and caries-free adolescents using SNP SpaP_3458_ reflected the expected B-1 and non–B-1 types.

### Geomapping of *S. mutans* adhesin types

Residential address coordinates for each individual infected with specific *S. mutans* SpaP A/B/C and Cnm/Cbm adhesion phenotypes were identified and plotted on a map using Google maps. The individual plots were allocated to northern and southern regions of the county of Västerbotten in northern Sweden. Each of the two regions was divided into urban and rural areas.

### Analyses of Cnm expression and glycosylation of *S. mutans Cnm* and *Cbm* wild-type isolates from the adolescents

A panel of 12 *cnm* and five *cbm* strains isolated from the adolescents^12^ was analysed for Cnm expression and glycosylation, using a combination of *cnm* and *cbm* gene structures and western blot experiments with a glycan detection kit and anti-Cbm and Cnm antisera.

#### Sequence structures of *cnm* and *cbm*

Each full-length *cnm* and *cbm* gene was amplified by PCR using three specific primer pairs: the forward (F) and reverse (R) primer pairs (1–3) as described for *cnm* elsewhere^12^ and for *cbm*: 1F, GACAAACTAATGAAATCTAA; 1R, TCATCAGGAACCAGCGCACA; 2F, AGCTGAAGTTAGTGTTGTAA; 2R, ATGCCG CCGGCAGCATTAAC; 3F, CAATAGTAAAGCTTGGTACA; and 3R, GCAAAAACTGTT GTCCCTGC. Amplified fragments were sequenced forward and in reverse by dideoxy chain termination/cycling sequencing on an ABI 3730 XL machine (Eurofins Genomics, Germany). Assembly of the full gene was done using CodonCode software, with translation into protein sequences using MEGA 7 software.^37^ Alignment of Cnm and Cbm sequences was performed in the Clustal Omega program for protein homology between and within Cnm and Cbm strains.

#### *Cnm* and *Cbm* membrane extracts

A bacterial suspension (1 mL; 6 × 10^9^ cells/mL) was centrifuged at 14,000 rpm for 10 min. After removal of supernatants, 100 μL of spheroplasting buffer (20 mM Tris–HCl, pH 6.8, 10 mM MgCl_2_, 26% w/v raffinose, 500 U mutanolysin, 0.5 mM phenylmethylsulfonyl fluoride) was added to the bacterial pellet and incubated for 30 min at 37 °C, followed by centrifugation at 14,000 rpm for 15 min at 4 °C and storage of the extracts at −20 °C until use.

#### Western blot glycan and antisera detection

For western blot analyses of membrane extracts, we used a glycosylation kit (a glycoprotein detection system, GE Healthcare, RPN2190) and Cnm- and Cbm-specific antisera (generated by Agrisera, Umeå, Sweden, using recombinant Cnm and Cbm proteins as antigens). Membrane extracts (5 μL) were separated by sodium dodecyl sulphate (SDS)-polyacrylamide gel electrophoresis on 4%–15% gradient gels (Mini-PROTEAN TGX Precast Protein Gels, 10-well, 30 μL, Bio-Rad, 4,561,083) and transferred to a polyvinylidene fluoride membrane (pore size 0.2 μm, Roche, 03010040001) using the Trans-Blot Turbo Transfer System (Bio-Rad).

Validation of antisera against Cnm or Cbm was performed on a western blot with cell wall extracts from *S. mutans* reference strains, showing only bands of expected molecular weights for Cnm and Cbm in Cnm- and Cbm-positive isolates, respectively. No bands were detected when SpaP A, B, or C isolates negative for *cnm* or *cbm* were used. The Cnm- and Cbm-antisera did not show cross-reactivity, and no antibodies against Cnm or Cbm were detected in the preserum from immunisation.

The membrane intended for glycan detection was washed with phosphate-buffered saline (PBS; 25 mM phosphate, 85 mM NaCl, pH 7.4) and incubated in 100 mM acetate buffer (pH 5.5) with 10 mM sodium metaperiodate in darkness. After three washes with PBS, modified carbohydrates were biotinylated by incubating the membrane in 20 mL of 100 mM acetate buffer (pH 5.5) with 4 μL of 0.123 mM biotin hydrazide for 1 h. After three washes with PBS, the membrane was incubated with 5% milk and then incubated with 20 mL of streptavidin–horseradish peroxidase (diluted 1:6000 in PBS-Tween) for 30 min. After another three washes, detected proteins were visualised with enhanced chemiluminescence reagent (SuperSignal West Dura Extended Duration Substrate, Bio-Rad, 34,075) in ChemiDoc XRS (Bio-Rad).

The membrane intended for Cnm and Cbm detection with antisera was washed three times with TBS-Tween (50 mM Tris, 150 mM NaCl, and 0.05% Tween-20, pH 7.4), blocked with blocking buffer (TBS-T containing 5% w/v non-fat dried milk) for 1 h, and incubated with Cnm- and Cbm-specific antisera (1:3000 in blocking buffer) for 1 h. After three washes with TBS-T, the membranes were incubated for 1 h with horseradish peroxidase–conjugated polyclonal goat anti-rabbit IgG (Dako Denmark, P0448) in blocking buffer. After three more washes, detected proteins were visualised as described above.

### *In vitro* and *in situ* properties of *S. mutans* SpaP A/B/C and Cnm/Cbm reference isolates from participants

The following representative reference isolates of the SpaP A/B/C and Cnm/Cbm adhesion phenotypes and associated mutants^12^ were used for binding to human buccal mucosal tissues *in situ*, biofilm formation, and acid/oxygen tolerance: *spaP A* (isolate 98), *spaP B* (isolate 189), *spaP C* (isolate 449), *cnm* (isolate 56, SpaP A), and *cbm* (isolate 422, SpaP B). In addition, we used three knockout mutants generated from *cnm*, *spaP A* isolate 56: spaP *A*-negative, *cnm*-negative, and spaP- and cnm-negative.^12^ In binding experiments, we used recombinant Cnm and Cnm proteins generated at the Protein Expertise Platform, Umeå University. Briefly, *cnm* and *cbm* genes were cloned into the expression vector pETHis1a and expressed in Rosetta cells. Expressed proteins were purified with a Ni-NTA purification system (Novex, Life Technologies, catalogue number K950-01) and Superdex 200 gel filtration chromatography (Sigma–Aldrich) and stored at −80 °C until use.

#### Dot blot binding to extracellular proteins

A dot blot assay was used to assess binding of *S. mutans* to collagen types I–VI, casein, and albumin. Briefly, 1 μL of serial dilutions of each protein (500 ng/μL, 100 ng/μL, 20 ng/μL, 4 ng/μL, 0.8 ng/μL, 0.16 ng/μL) was spotted onto a nitrocellulose membrane (0.45 μm, Bio-Rad) and allowed to dry. The membrane was then blocked with 5% bovine serum albumin (BSA, Sigma–Aldrich, A7030) in PBS-T for 2 h while rotating, washed three times with PBS-T, and incubated with 1 mL biotinylated bacterial suspension (5 × 10^7^ cells/mL) in ADH buffer (1 M KCl, 0.1 M CaCl_2_ × 2H_2_O, 0.01 M MgCl_2_ × 6H_2_O, 0.1 M K_2_HPO_4_, 0.1 M KH_2_PO_4_) at +4 °C overnight. After four washes with PBS-T, the membrane was incubated with horseradish peroxidase–conjugated streptavidin (1:10,000 in ADH buffer) for 1 h while rotating. After three washes with PBS-T, substrate (SuperSignal West Dura Extended Duration Substrate, Bio-Rad, 34,075) was added to the membrane, and bound bacteria were detected with ChemiDoc XRS (Bio-Rad). Sources of protein ligands were collagen types I (Sigma–Aldrich, C2249), II–V (Millipore; CC052, CC054, CC076, CC077, respectively), and VI (Abcam, 7538), casein (Sigma–Aldrich, C6905), and BSA (Sigma–Aldrich, A7030).

#### Binding to buccal mucosal tissues in situ

For bacterial binding to a buccal mucosa tissue section, a biopsy from a human volunteer and bacterial cells were treated as described below.^38^ Bacterial cells were labelled with fluorescein isothiocyanate (FITC) (Sigma–Aldrich, 46,950), washed three times in PBS-T, adjusted to optical density (OD) 1.0 at A600 nm, and resuspended in carbonate buffer (0.1 M carbonate, 0.15 M NaCl, pH 9). FITC (10 mg/mL in dimethyl sulphoxide) was added (10 μL/mL of bacterial suspension) and incubated for 8 min in darkness. After incubation, the bacteria were washed until the supernatant was no longer yellow. The bacterial pellet was then washed once in blocking buffer (PBS-T containing 1% w/v BSA), and the OD adjusted to 1.0 at 600 nm in blocking buffer. Before bacteria binding, the buccal mucosal tissue section was deparaffinised in xylene and isopropanol, rehydrated in ethanol and PBS, and incubated in blocking buffer for at least 1 h. After removal of blocking buffer, 100 μL FITC-labelled bacteria (OD 0.2 at 600 nm) was added to the tissue slide, incubated for at least 2 h in darkness, and washed by submersion in PBS-T 50 × 3 times. After being dried, the tissue sections were left in mounting medium in darkness at 4 °C overnight, and bound bacteria were detected with a fluorescence microscope (Zeiss).

#### Biofilm formation on saliva-coated surfaces

Biofilm formation was achieved on saliva-coated channel slides (μ-Slide VI 0.4, Ibidi, 80,606).^39^ Parotid saliva (100 μL) pooled from several individuals and diluted 1:1 in PBS with 0.3 M CaCl_2_ was added to each lane of the channel slide and incubated overnight. The lanes were rinsed in 100 μL PBS three times to remove excess saliva and then incubated with 120 μL per lane of bacterial suspensions in log phase (OD 0.8 at 600 nm) in minimal medium MM4 containing 40 mM phosphate/citrate buffer (pH 7.5) and 20 mM glucose for 2 h at 37 °C in nitrogen in a 5% CO_2_ atmosphere. After the incubation, the channels were rinsed three times with MM4 medium, and adhered cells were stained with 60 μL LIVE/DEAD BacLight viability stain (Invitrogen, L7007) per lane to visualise the initial biofilm formation under a fluorescence microscope (confocal laser scanning microscopy).

#### Acid/oxygen tolerance by *S. mutans* isolates

Bacterial suspensions (120 μL, log phase OD 0.8 at 600 nm) in MM4 medium were added directly to lanes of channel slides and incubated for 2 h at 37 °C in nitrogen under a 5% CO_2_ atmosphere, followed by removal of non-adherent cells by two rinses with pH 7.5 MM4 medium.^40^ For acid adaptation, MM4 medium adjusted to sublethal pH 5.5 was added to the lanes and incubated for 2 h prior to exposure to pH 3.5 MM4 medium for an additional 2-h incubation. For acid tolerance without adaptation, pH 3.5 MM4 medium was added directly to the lanes and incubated for 2 h. Cells incubated in pH 7.5 MM4 medium were used as control. Dead and viable cells were visualised by addition of 60 μL LIVE/DEAD BacLight viability stain (Invitrogen, L7007) and counted as described above.

### A nested study of *S. mutans* infection and counts in repeated saliva samples

In a nested study using 71 dental student volunteers, we evaluated the behaviour of *S. mutans* infection and load over time using three repeated measures. Whole saliva was collected three times at 4-week intervals from the volunteers by having them chew on paraffin. Saliva (200 μL) was centrifuged at 12,000 rpm for 10 min, and the pellet was resuspended in 100 μL 0.2% SDS and incubated at 96 °C for 10 min. After a second centrifugation (12,000 rpm, 5 min), 1 μL of supernatant with released DNA was analysed for *S. mutans per se* as described above.

### Statistics

Quantitative differences in numbers of DeFS caries lesions at ages 12 and 17 years and of 5-year increment ΔDeFS are given as mean differences with 95% confidence intervals (CIs; using bootstrapping), along with means ± standard deviations (SDs) for comparability with previous relevant studies [*e.g.,* 11, 12]. To ensure the fullest possible perspective on the data, we also report medians with interquartile ranges (IQRs) for positively skewed caries and microbiota data. These sets of findings are complementary and collectively give a full view of the underlying data distribution and structure. The visualised quantitative data and data trends were interpreted in terms of fold and mean difference in mean/median values, combined with P-values and mean difference with 95% CI and total partial least squares (PLS) models. This established compatibility with our hypotheses and avoided overreliance on statistical significance.^41^ Of the ∼200 analyses performed (Table 1, Table 2, Table 3, Table 4), about 33% (n = 65/195) showed associations at the level of P < 0.05, supporting a data structure compatible with our conclusions. Mann–Whitney U tests were used because of the large spread and skewed distribution of the caries and microbiota data. To analyse proportions, we used Fisher's exact test except for the use of chi-square test for contingency tables exceeding 2 × 2. All analyses used SPSS software (version 28). We performed analyses for specific hypotheses/questions to avoid screening of variables that would justify adjustment for multiple comparisons. The statistical analyses used two-tailed tests, with P < 0.05 considered significant. The PLS models, which relate two data matrices (*X* and *Y*) to each other, used Simca software (version 17) to establish and visualise the influence of the P4a^+^ and P4a^−^ (*e.g*., P6, P1) phenotypes on the microbial profile in caries. The PLS models show the ability of the *X* variables to explain (R^2^) or predict (Q^2^) the variation in *Y*. PLS1 models with a single Y caries outcome measure were used. The predictive ability (Q^2^) was estimated by cross-validation via PLS modelling in seven repeated blocks that excluded all participants once. Skewed data were log transformed by established norm and auto-scaled to unit variance before PLS models were generated.

**Table 1:** Influence of *S. mutans* SpaP A/B/C adhesion types on caries progression in high (P4a), moderate (P6), and low (P1) caries genotypes and phenotypes.

Types^a^	n	DeFS-12 y^b^			n	DeFS-17 y^b^			ΔDeFS-5y^c^		
		mean ± SD	P^d^	mean diff (95% CI)^e^		mean ± SD	P^d^	mean diff (95% CI)^e^	mean ± SD	P^d^	mean diff (95% CI)^e^
P4a											
SpaP A	19	3.7 ± 2.9	0.075	1.4 (−0.2, 2.9)	16	9.8 ± 11.0	0.51	3.4 (−1.6, 10.1)	6.0 ± 10.9	0.61	2.2 (−2.5, 8.7)
SpaP B	11	4.7 ± 5.0	0.13	2.3 (−0.6, 5.6)	9	12.0 ± 10.0	0.025	5.6 (0.0, 13.5)	6.1 ± 4.6	0.065	2.2 (−0.7, 5.8)
SpaP C	2	3.5 ± 0.7	0.34	1.1 (0.0, 2.2)	2	8.5 ± 5.0	0.35	2.1 (−2.7, 7.0)	5.3 ± 5.0	0.48	1.5 (−3.0, 6.0)
SpaP neg	42	2.4 ± 2.5	Ref.	Ref.	37	6.4 ± 6.3	Ref.	Ref.	3.8 ± 4.5	Ref.	Ref.
P6											
SpaP A	25	3.2 ± 2.8	0.0077	1.7 (0.5, 3.0)	24	7.3 ± 6.0	0.033	3.3 (0.7, 5.9)	4.1 ± 4.2	0.11	1.7 (−0.1, 3.6)
SpaP B	17	3.9 ± 3.3	0.0013	2.4 (1.0, 4.3)	11	13.6 ± 16.8	0.0044	9.6 (1.8, 21.2)	8.4 ± 13.4	0.11	6.0 (−0.3, 15.0)
SpaP C	4	3.3 ± 2.5	0.11	1.8 (−1.2, 4.5)	4	7.3 ± 6.6	0.29	3.3 (−3.9, 11.7)	3.6 ± 5.4	0.84	1.1 (−2.6, 8.8)
SpaP neg	56	1.5 ± 1.7	Ref.	Ref.	54	4.0 ± 3.8	Ref.	Ref.	2.5 ± 2.8	Ref.	Ref.
P1											
SpaP A	23	1.8 ± 1.8	0.42	0.2 (−0.7, 1.1)	21	6.3 ± 8.3	0.47	2.4 (−1.2, 6.6)	4.4 ± 6.8	0.40	1.9 (−0.8, 5.4)
SpaP B	17	3.1 ± 3.1	0.067	1.5 (−0.1, 3.0)	14	6.7 ± 4.3	0.013	2.8 (0.1, 5.2)	3.7 ± 3.2	0.048	1.3 (−0.7, 3.3)
SpaP C	6	3.0 ± 2.0	0.18	1.4 (−0.1, 3.1)	5	4.4 ± 3.6	0.95	0.4 (−3.0, 4.1)	1.8 ± 2.2	0.60	−0.7 (−2.6, 1.8)
SpaP neg	69	1.6 ± 2.1	Ref.	Ref.	56	4.0 ± 4.8	Ref.	Ref.	2.5 ± 4.0	Ref.	Ref.

a High (P4a), moderate (P6), and low (P1) caries phenotypes with Swedish ethnicity defined by *PRH1*, *PRH2* genetic variation.

b Caries DeFS (Decayed, enamel-included, Filled Surfaces) at 12 and 17 years of age.

c ΔDeFS (5 y) = 5-year prospective caries increment from 12 to 17 years of age.

d 2-sided P value from Mann–Whitney U test.

e Mean difference estimate with 95% CIs by bootstrapping 1000 times.

**Table 2:** Influence of load of *S. mutans*, lactobacilli, and streptococci on caries progression in the high (P4a), moderate (P6), and low (P1) caries phenotypes defined by *PRH1*, *PRH2* genetic variation.

Types^a^	Load	n	DeFS-12 y^b^			n	DeFS-17 y^b^			ΔDeFS-5y^c^		
			mean ± SD	P^d^	mean diff (95% CI)^i^		mean ± SD	P^d^	mean diff (95% CI)^i^	mean ± SD	P^d^	mean diff (95% CI)^i^
*S. mutans*^e^^,^^f^												
P4a	Negative	34	2.1 ± 2.2	Ref.	Ref.	30	6.9 ± 8.5	Ref.	Ref.	4.7 ± 7.8	Ref.	Ref.
	Low	21	3.6 ± 3.3	0.086	1.5 (0.1, 3.3)	16	7.4 ± 5.4	0.23	0.5 (−4.0, 4.4)	3.3 ± 3.8	0.84	−1.5 (−5.3, 1.5)
	High	20	4.2 ± 3.8	0.033	2.0 (0.2, 3.8)	19	10.6 ± 9.6	0.061	3.7 (−1.8, 9.6)	6.0 ± 6.4	0.25	1.3 (−2.8, 5.2)
P6	Negative	55	1.4 ± 1.8	Ref.	Ref.	51	3.9 ± 3.7	Ref.	Ref.	2.3 ± 2.9	Ref.	Ref.
	Low^f^	21	2.0 ± 2.0	0.31	0.5 (−0.5, 1.5)	19	5.6 ± 5.7	0.56	1.7 (−1.0, 4.7)	3.5 ± 4.1	0.34	1.2 (−0.7, 3.4)
	High^f^	26	4.6 ± 3.0	<0.0001	3.1 (2.0, 4.4)	23	11.5 ± 12.2	<0.0001	7.6 (3.4, 13.5)	6.7 ± 9.5	0.0024	4.4 (1.1, 9.0)
P1	Negative	61	1.5 ± 2.1	Ref.	Ref.	50	3.6 ± 4.0	Ref.	Ref.	2.1 ± 2.8	Ref.	Ref.
	Low^f^	26	2.0 ± 2.3	0.25	0.5 (−0.5, 1.5)	21	4.9 ± 5.9	0.64	1.3 (−1.2, 4.3)	3.3 ± 5.8	0.84	1.2 (−1.1, 4.1)
	High^f^	28	2.8 ± 2.4	0.0069	1.3 (0.3, 2.4)	25	7.6 ± 7.4	0.0041	4.0 (1.0, 7.1)	4.7 ± 6.1	0.024	2.5 (0.3, 5.2)
Lactobacilli^e^^,^^g^												
P4a	Negative	36	3.0 ± 3.7	Ref.	Ref.	33	6.2 ± 7.4	Ref.	Ref.	3.2 ± 4.2	Ref.	Ref.
	Low	23	3.0 ± 2.5	0.46	−0.0 (−1.6, 1.6)	18	7.3 ± 4.9	0.18	1.0 (−2.7, 4.4)	3.8 ± 3.8	0.37	0.6 (−1.7, 3.0)
	High	16	3.5 ± 2.8	0.27	0.5 (−1.4, 2.3)	14	13.4 ± 11.4	0.019	7.2 (0.7, 13.7)	9.5 ± 11.0	0.062	6.3 (0.6, 12.7)
P6	Negative	54	1.9 ± 2.6	Ref.	Ref.	49	4.0 ± 4.0	Ref.	Ref.	2.2 ± 2.9	Ref.	Ref.
	Low	28	2.4 ± 2.5	0.29	0.5 (−0.7, 1.7)	26	8.4 ± 12.3	0.093	4.4 (0.2, 9.6)	5.4 ± 9.3	0.078	3.2 (0.2, 7.2)
	High	20	3.5 ± 2.0	0.0015	1.6 (0.5, 2.6)	18	8.7 ± 5.0	0.00047	4.7 (2.0, 7.3)	4.9 ± 4.1	0.0060	2.7 (0.6, 4.8)
P1	Negative	68	1.6 ± 2.0	Ref.	Ref.	55	4.4 ± 4.9	Ref.	Ref.	2.8 ± 4.1	Ref.	Ref.
	Low	31	2.1 ± 2.7	0.33	0.6 (−0.4, 1.7)	27	5.0 ± 6.5	0.90	0.7 (−1.9, 3.5)	3.3 ± 5.6	0.98	0.6 (−1.6, 3.0)
	High	16	3.1 ± 2.2	0.0037	1.6 (0.5, 2.8)	14	6.9 ± 6.6	0.077	2.6 (−0.9, 6.7)	3.6 ± 4.8	0.63	0.8 (−1.6, 3.7)
Streptococci^e^^,^^h^												
P4a	Low	21	2.5 ± 1.8	Ref.	Ref.	18	6.1 ± 6.9	Ref.	Ref.	3.1 ± 5.0	Ref.	Ref.
	Moderate	26	3.5 ± 3.9	0.67	1.0 (−0.5, 2.9)	24	8.8 ± 9.5	0.52	2.7 (−2.3, 7.6)	5.1 ± 5.7	0.20	1.9 (−1.3, 4.9)
	High	28	3.1 ± 3.2	0.85	0.7 (−0.8, 2.0)	23	8.9 ± 7.9	0.056	2.8 (−1.8, 7.2)	5.7 ± 8.4	0.25	2.6 (−1.2, 6.8)
P6	Low	29	2.1 ± 2.4	Ref.	Ref.	27	4.3 ± 6.3	Ref.	Ref.	2.2 ± 4.0	Ref.	Ref.
	Moderate	36	2.6 ± 2.9	0.48	0.5 (−0.8, 1.8)	32	8.5 ± 10.5	0.014	4.2 (0.0, 8.6)	5.5 ± 8.3	0.019	3.3 (0.3, 6.7)
	High	37	2.3 ± 2.3	0.56	0.3 (−0.9, 1.3)	34	5.3 ± 4.6	0.20	1.0 (−2.3, 3.5)	3.0 ± 3.0	0.18	0.7 (−1.3, 2.4)
P1	Low	19	2.1 ± 2.0	Ref.	Ref.	15	3.3 ± 2.8	Ref.	Ref.	1.3 ± 2.4	Ref.	Ref.
	Moderate	56	1.9 ± 2.3	0.55	−0.2 (−1.3, 0.9)	46	5.0 ± 5.6	0.45	1.7 (−0.5, 3.9)	3.3 ± 4.6	0.084	2.0 (0.3, 3.8)
	High	40	1.9 ± 2.4	0.39	−0.3 (−1.5, 1.0)	35	5.4 ± 6.6	0.43	2.1 (−0.3, 4.9)	3.5 ± 5.3	0.14	2.2 (0.2, 4.5)

a High (P4a), moderate (P6), and low (P1) caries phenotypes with Swedish ethnicity defined by *PRH1*, *PRH2* genetic variation.

b Caries DeFS (Decayed, enamel-included, Filled Surfaces) at 12 and 17 years of age.

c ΔDeFS (5 y) = 5-year prospective caries increment from 12 to 17 years of age.

d 2-sided P value from Mann–Whitney U test.

e *S. mutans*, Lactobacilli and Streptococci load in whole saliva measured by culture counts at 12 years of age.

f *S. mutans* infection and load (Negative <10,000 CFU, Low 10,000–300,000 CFU, High >300,000 CFU).

g Lactobacilli infection and load (Negative <20,000 CFU, Low 20,000–100,000 CFU, High >100,000 CFU).

h Streptococci infection and load (Low <10,000,000 CFU, Moderate 10,000,000–50,000,000 CFU, High >50,000,000 CFU).

i Mean difference estimate with 95% CIs by bootstrapping 1000 times.

**Table 3:** Influence of load of *S.mutans*, lactobacilli and streptococci on caries progression in 452 adolescents.

Bacterial load	n	DeFS-12 y^c^			n	DeFS-17 y^c^			ΔDeFS-5y^d^		
Percentile^a^^,^^b^ (CFU)		mean ± SD	P^e^	mean diff (95%CI)^f^		mean ± SD	P^e^	mean diff (95% CI)^f^	mean ± SD	P^e^	mean diff (95% CI)^f^
*S. mutans*^a^											
Neg (0)	185	1.5 ± 2.0	0.0033	−1.0 (−1.8, −0.3)	166	4.6 ± 5.6	0.021	−1.7 (−3.6, 0.1)	3.0 ± 5.1	0.076	−0.7 (−2.1, 0.6)
<*P*20 (1–32960)	53	2.5 ± 2.4	Ref.	Ref.	41	6.2 ± 5.4	Ref.	Ref.	3.8 ± 3.9	Ref.	Ref.
*P*20-40 (32,961–98100)	53	2.7 ± 2.9	0.87	0.2 (−0.9, 1.2)	44	7.0 ± 6.2	0.78	0.8 (−1.8, 3.4)	4.2 ± 4.7	0.85	0.4 (−1.4, 2.3)
*P*40-60 (98,101–508000)	54	3.2 ± 2.7	0.19	0.7 (−0.3, 1.7)	46	6.8 ± 6.5	0.74	0.6 (−1.9, 3.0)	3.8 ± 5.8	0.53	0.1 (−1.9, 2.1)
*P*60-80 (508,001–1380,000)	54	4.4 ± 3.4	0.0021	1.9 (0.8, 3.1)	46	13.1 ± 12.1	0.00086	6.8 (3.4, 10.7)	8.2 ± 10.1	0.025	4.4 (1.6, 7.6)
>*P*80 (>1,380,000)	53	3.8 ± 2.9	0.021	1.2 (0.2, 2.3)	47	9.7 ± 8.2	0.026	3.5 (0.8, 6.4)	5.7 ± 5.8	0.090	2.0 (−0.0, 4.0)
Lactobacilli^b^											
Neg (0)	61	1.5 ± 1.9	0.32	−0.6 (−1.4, 0.2)	53	3.8 ± 3.5	0.056	−2.1 (−3.9, −0.4)	2.1 ± 2.6	0.050	−1.5 (−2.7, −0.3)
<*P*20 (1–2480)	78	2.1 ± 2.8	Ref.	Ref.	71	5.9 ± 6.0	Ref.	Ref.	3.6 ± 4.1	Ref.	Ref.
*P*20-40 (2480–12720)	78	2.4 ± 2.9	0.64	0.3 (−0.6, 1.1)	65	6.5 ± 8.4	0.42	0.6 (−1.7, 3.2)	4.2 ± 7.5	0.38	0.5 (−1.3, 2.6)
*P*40-60 (12,721–32400)	78	2.9 ± 2.7	0.020	0.8 (−0.0, 1.7)	67	6.3 ± 5.3	0.43	0.5 (−1.5, 2.2)	3.4 ± 3.8	0.93	−0.2 (−1.5, 1.1)
*P*60-80 (32,401–100702)	79	2.9 ± 2.7	0.026	0.7 (−0.2, 1.6)	67	8.6 ± 10.7	0.31	2.8 (0.2, 5.8)	5.7 ± 8.5	0.42	2.1 (−0.0, 4.5)
>*P*80 (>100,702)	78	3.6 ± 2.9	<0.0001	1.5 (0.5, 2.4)	67	9.9 ± 7.9	0.00025	4.0 (1.9, 6.5)	6.1 ± 6.7	0.015	2.5 (0.8, 4.4)
Streptococci^b^											
Neg (0)	1	3.0	–	–	1	3.0	–	–	0.0	–	–
<*P*20 (1–9140,000)	90	2.4 ± 2.5	Ref.	Ref.	79	5.7 ± 7.7	Ref.	Ref.	3.2 ± 6.1	Ref.	Ref.
*P*20-40 (9,140,001–26,000,000)	88	2.5 ± 2.6	0.92	0.0 (−0.7, 0.8)	78	7.0 ± 8.4	0.12	1.2 (−1.3, 3.8)	4.5 ± 6.6	0.030	1.3 (−0.6, 3.2)
*P*40-60 (26,000,001–46,000,000)	91	2.6 ± 2.9	0.95	0.1 (−0.7, 0.9)	76	6.8 ± 7.0	0.058	1.0 (−1.5, 3.3)	4.1 ± 4.6	0.015	0.9 (−0.9, 2.5)
*P*60-80 (46,000,001–85,000,000)	90	2.7 ± 2.7	0.49	0.3 (−0.5, 1.0)	76	7.1 ± 6.7	0.0096	1.4 (−0.8, 3.7)	4.5 ± 6.2	0.012	1.3 (−0.6, 3.4)
>*P*80 (>85,000,000)	92	2.7 ± 3.0	0.85	0.2 (−0.6, 1.0)	79	7.9 ± 8.1	0.014	2.1 (−0.2, 4.6)	5.0 ± 6.8	0.0047	1.8 (−0.1, 3.8)

a Percentiles (*P*) of *S. mutans* quantity measured by qPCR (CFU) in whole saliva at 12 years of age.

b Percentiles *(P)* of Lactobacilli and total Streptococci quantity measured by culture (CFU) of whole saliva at 12 years of age.

c Caries DeFS (Decayed, enamel included, Filled Surfaces) at 12 and 17 years of age.

d ΔDeFS (5 y) = 5-years prospective caries increment from 12 to 17 years of age.

e 2-sided P value from Mann–Whitney U-test.

f Mean difference estimate with 95% CIs by bootstrapping 1000 times.

**Table 4:** Influence of residency and load of *S. mutans* dominant and mixed adhesion types on caries progression in 452 adolescents.

*S. mutans*	Infection^a^		Freq & stab.^b^			DeFS-12 y^c^		DeFS-17 y^c^		ΔDeFS-5y^d^	
	12 y	17 y	%	n	(%)	m ± SD	P^e^	m ± SD	P^e^	m ± SD	P^e^
*S. m*	+	+	40.7	158	85	3.5 ± 3.1	<0.0001	9.8 ± 9.1	<0.0001	6.0 ± 7.3	<0.0001
	+	–	7.0	27		2.5 ± 2.5	0.05	5.1 ± 5.0	0.41	2.8 ± 5.0	0.56
	–	+	14.9	58		1.8 ± 1.9	0.31	5.7 ± 6.0	0.05	3.6 ± 5.3	0.07
	–	–	37.4	145		1.6 ± 2.0	Ref.	4.5 ± 5.6	Ref.	2.9 ± 4.7	Ref.
Refs f	+			217		3.6 ± 3.0	<0.0001	9.2 ± 8.8	<0.0001	5.5 ± 7.1	<0.0001
	–			235		1.7 ± 2.1	Ref.	4.9 ± 5.7	Ref.	3.1 ± 4.9	Ref.
Mixed SpaP ABC^f^	B	mix	2.1	8		4.6 ± 3.2	0.12	15.6 ± 13.9	0.07	10.8 ± 12.9	0.21
	–	mix	2.3	9		3.0 ± 1.5	0.92	11.2 ± 10.4	0.53	8.1 ± 11.2	0.38
	mix	B	0.8	3		5.3 ± 4.6	0.29	13.7 ± 11.6	0.30	8.0 ± 8.9	0.46
	mix	A	1.8	7		3.7 ± 2.4	0.40	12.6 ± 12.3	0.37	7.8 ± 8.8	0.39
	Cnm	Cnm	4.4	17	85	3.9 ± 2.2	0.11	11.2 ± 9.2	0.12	6.9 ± 7.5	0.21
	mix	mix	2.6	10	37	4.7 ± 3.3	0.10	11.6 ± 7.1	0.10	6.6 ± 4.9	0.17
	A	A	18.6	72	80	3.0 ± 3.0	0.76	8.7 ± 8.7	0.87	5.5 ± 7.4	0.81
	B	B	6.4	25	51	3.9 ± 3.9	0.39	10.5 ± 12.2	0.33	5.8 ± 9.0	0.56
	mix/dom^g^	mix/dom^g^	8.8	34		4.5 ± 3.0	0.02	12.6 ± 9.9	0.02	7.7 ± 8.2	0.10
	dom^g^	dom^g^	29.0	114		3.2 ± 3.1	Ref.	9.1 ± 9.3	Ref.	5.5 ± 7.5	Ref.
SpaP B-1			3.3	15		5.9 ± 3.4	0.00	15.1 ± 11.7	0.03	10.4 ± 11.8	0.07
SpaP B-1 excl^h^			12.3	56		3.7 ± 3.4	0.29	10.6 ± 10.9	0.16	6.0 ± 8.0	0.26
Load *P*_60-80_^i^	B			17		5.2 ± 4.3	0.04	18.2 ± 17.1	0.01	12.2 ± 14.3	0.03
	Cnm			6		5.0 ± 2.3	0.06	17.2 ± 14.1	0.11	11.1 ± 12.6	0.36
	B-1			11		6.3 ± 2.9	0.00	15.6 ± 13.6	0.09	10.2 ± 14.0	0.52
	mix			16		5.3 ± 3.0	0.01	14.4 ± 10.9	0.01	9.3 ± 10.0	0.04
	A			23		3.7 ± 3.2	0.39	11.0 ± 9.3	0.17	6.9 ± 8.3	0.29
Load > *P*_90_^i^	+	+		3		5.7 ± 5.3	0.25	25.3 ± 16.6	0.04	20.8 ± 16.5	0.02
	+	–		21		3.3 ± 2.7	0.56	8.0 ± 6.6	0.80	5.1 ± 5.0	0.52
	+			24		3.6 ± 3.0	0.36	10.3 ± 10.0	0.35	7.2 ± 8.8	0.18
Refs	A		24.6	111		3.0 ± 2.8	Ref.	8.4 ± 8.2	Ref.	5.2 ± 7.1	Ref.
	B		15.7	71		4.2 ± 3.5	0.03	11.4 ± 11.1	0.05	6.7 ± 8.8	0.11
	C		4.2	19		3.3 ± 1.8	0.37	7.5 ± 4.8	0.75	4.3 ± 4.1	0.93
	Cnm		5.8	26		4.4 ± 2.6	0.01	10.4 ± 8.7	0.18	6.0 ± 7.2	0.46
	Cbm		1.5	7		2.9 ± 3.7	0.63	3.8 ± 2.8	0.20	2.4 ± 1.6	0.60

a Infection status at 12 and 17 years measured by qPCR or culturing (*P*_60-80_).

b Totally 388 adolescents were sampled for saliva at both 12 and 17 years of age.

c Caries DeFS (Decayed, enamel included, Filled Surfaces) at 12 and 17 years of age.

d ΔDeFS (5 y) = 5-years prospective caries increment from 12 to 17 years of age.

e 2-sided P value from Mann–Whitney U-test.

f Mixed (mix) marks adolescents infected with mixed SpaP ABC type, dom marks adolescents infected with dominant SpaP A/B/C type.

g Mix/dom, adolescents infected with mixed SpaP regardless of a stable infection status (mix,mix) or switching between dominant and mixed SpaP (mix, dom or dom,mix).

h SpaP B-1 excl marks adolescents infected with non-B-1 SpaP B type.

i *P* marks Percentile from culturing (*P*_60-80_) or qPCR (>*P*_90_).

### Ethics

The current study was approved by the ethics committee for human experiments at Umeå University, Sweden (reference number: 08-047M, 2010-253-32M). Informed consent was signed by all parents.

### Role of funders

The funding source of the present study had no role in the design of this study, data collection, analyses, interpretation of the data, or writing of the manuscript.

## Results

### The *PRH1*, *PRH2* susceptible P4a/P6 and resistant P1 caries phenotypes fit different microbial profiles in caries

**Fig. 2:**
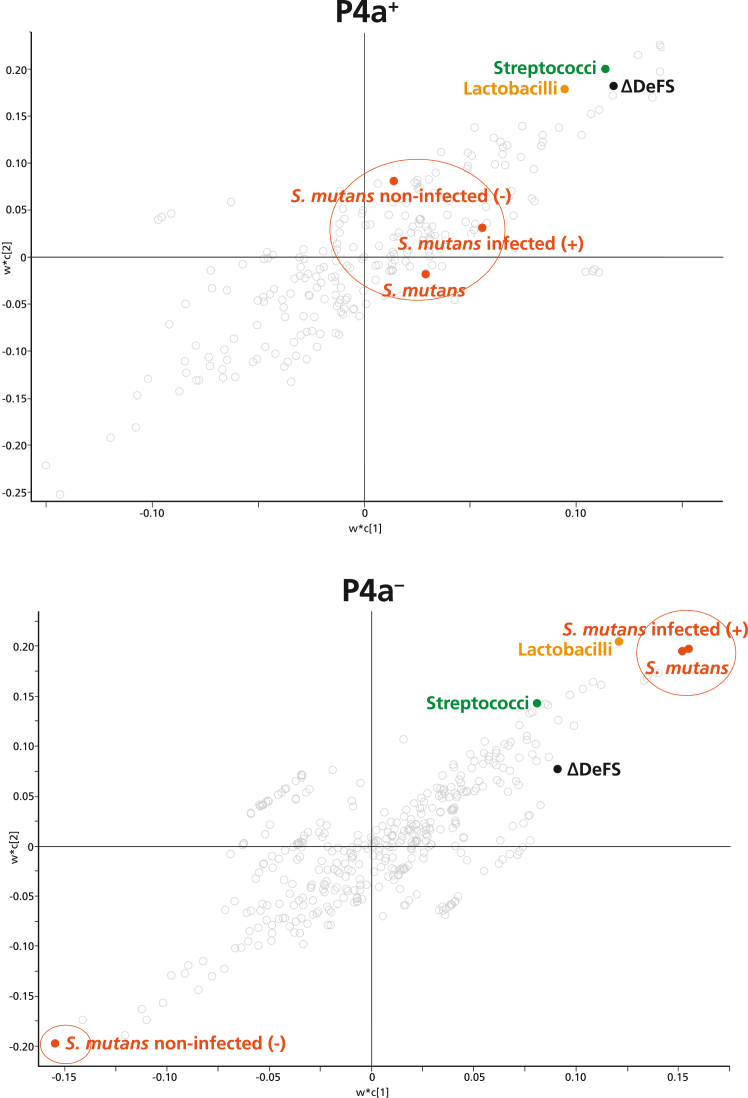
**Differential influence of *S. mutans* versus oral streptococci and lactobacilli on caries progression in P4a**^**+**^**versus P4a**^**−**^**(P6, P1) phenotypes**. The PLS model for P4a^+^ (R^2^ = 0.552, Q^2^ = −0.291) and P4a^−^ (R^2^ = 0.297, Q^2^ = 0.137) phenotypes used X demographic, lifestyle, and genotyping data (grey circles) and microbiota data (coloured) and single Y caries ΔDeFS outcomes. The P4a^−^ phenotype includes the major P6 and P1 and other minor *PRH1*, *PRH2* variations or phenotypes.

We hypothesised that the microbial profile in caries would be influenced by *PRH1*, *PRH2* allelic variation that specifies highly genetic susceptible, high-caries P4a phenotypes versus moderate-caries P6 and low-caries P1 phenotypes. We therefore assessed how caries progression in association with the three phenotypes depended on infection with and load of a small panel of caries-associated bacteria. This panel consisted of *S. mutans* SpaP A/B/C adhesion phenotypes and oral commensal lactobacilli and streptococci, including species of low (*e.g.*, *S. gordonii*) to moderate (*e.g.*, *S. oralis*, *S. mitis* biovar 1) cariogenicity (Tables 1 and 2, Tables S1 and S2, Fig. 2).

We first used total PLS models to explore different microbial profiles on caries progression in highly genetic susceptible P4a^+^ versus P4a^−^ (*e.g.,* P6/P1) phenotypes (Fig. 2). The PLS model showed that caries ΔDeFS progression in P4a^+^ phenotypes was influenced by oral streptococci and lactobacilli, but not by *S. mutans* infection profile (+/−) or load, at baseline. The opposite was true for P4a^−^ phenotypes. This indicates different microbial dependencies on caries progression in highly genetic susceptible versus other human *PRH1*, *PRH2* groupings.

The results of quantitative mean/median differences and statistical estimates next indicated that caries progression in the P4a, P6 and P1 phenotypes had a broad microbial dependence in the order P4a > P6 > P1 (Tables 1 and 2, Tables S1 and S2). Caries progression with P4a phenotypes was high regardless of infection or load of *S. mutans* SpaP A/B/C, lactobacilli, or streptococci. This progression increased marginally (1.3-fold) with *S. mutans* load and markedly (1.8- and 2.9-fold, respectively) with commensal streptococci and lactobacilli loads, respectively. Caries progression in the P6 phenotype was markedly increased (3-fold) from SpaP B and *S. mutans* load *per se*. It also depended on SpaP A and was comparably low in adolescents not infected with *S. mutans*. This progression was markedly dependent on oral commensal streptococci and lactobacilli loads. By contrast, the low-caries P1 phenotype had the narrowest microbiota dependence, primarily requiring infection with *S. mutans* for caries progression. Infection with SpaP B and *S. mutans* load *per se* predicted up to 2-fold increased caries progression, but the load of commensal streptococci or lactobacilli did not. In addition, stratified analyses without the small group of adolescents treated with orthodontic multibrackets during adolescence yielded virtually identical results (Tables S3 and S4).

Infection with *S. mutans* SpaP B but not A or C was linked to increased caries at age 17 years with the P4a, P6, and P1 phenotypes, verifying the comparably high cariogenic potential of the SpaP B phenotypes (Table 1 and Table S1). To verify the significance of the different microbial P4a, P6, and P1 caries profiles, we established that commensal streptococci, lactobacilli, and *S. mutans* loads also influenced caries progression in the entire group of adolescents (Table 3 and Table S5). The loads of *S. mutans*, lactobacilli, and in particular the commensal streptococci predicted 1.5- to 2-fold increased 5-year caries progression. Commensal streptococci load did not coincide with baseline caries, but *S. mutans* and lactobacilli loads did.

### *S. mutans* SpaP A/B/C and Cnm/Cbm types show tropism (specificity) for individual hosts and plausible family distribution patterns

**Fig. 3:**
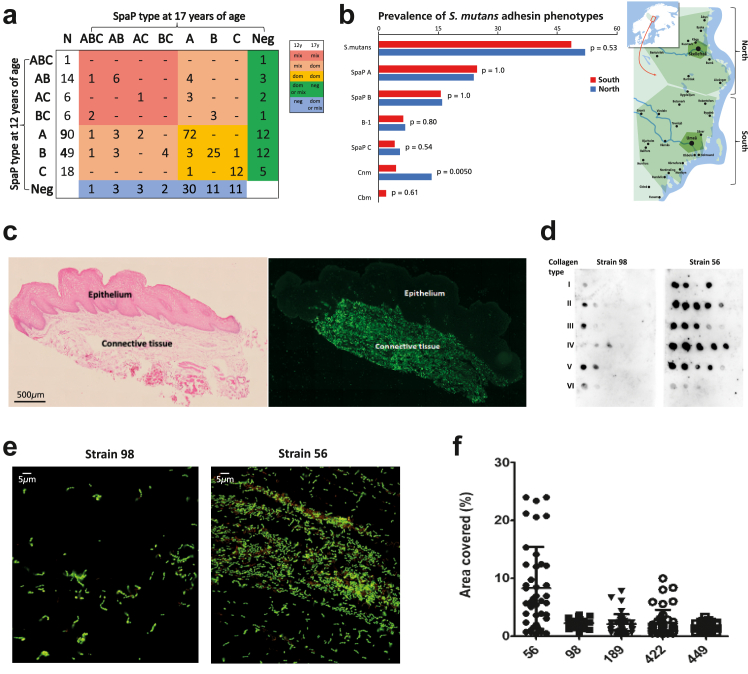
(**a**) Residence and changes in infection with *S. mutans* SpaP dominant and mixed adhesion types between ages 12 and 17 years measured by qPCR in whole saliva in 388 adolescents. (**b**) Different distribution of the adhesion types in the southern (Umeå) and northern (Skellefteå) regions of northern Sweden. Bars indicate the prevalence (%) of adolescents with *S. mutans*, dominant SpaP A/B/C, B-1 subtype, and Cnm/Cnm adhesion types in the south (n = 376) and north (n = 75) of Västerbotten County. The P value to the right of the bars relates to the difference in prevalence of each type between the two regions (Fisher's exact test). (**c**) *In situ* binding (right) of FITC-labelled *S. mutans* strain 56 (Cnm, SpaP A) to a histological tissue section of human buccal epithelium (histochemically stained to the left). Scale bar represents 500 μm. (**d**) Binding of strain 56 (Cnm, SpaP A) and strain 98 (SpaP A) to collagen types I–VI in a dot blot assay. Strain 56 bound avidly to collagen types I–V but not to type VI. (**e**) Microscopic images for biofilm formation of strain 56 (Cnm, SpaP A) and strain 98 (SpaP A) with highest and lowest area coverage, respectively. Scale bar represents 5 μm. (**f**) Biofilm formation of strains 56 (Cnm, SpaP A), 98 (SpaP A), 189 (SpaP B-1), 422 (Cbm, SpaP B-1), and 449 (SpaP C) on parotid saliva–coated surfaces. Results in c–f were replicated at least twice; see also Tables S8 and S12.

To explore the specificity (tropism) of *S. mutans* subtypes for individual hosts and possible family patterns, we analysed residency and interchange of dominant adhesion types in individuals during adolescence and their geographic distribution (Fig. 3, Table 4 and Tables S6 and S7). Only five adolescents showed switches between the dominant SpaP B/C types and none between the Cnm/Cbm types, and the highly persistent and prevalent SpaP A type never switched to SpaP B or C (Fig. 3a). Adolescents with *cnm* infection (n = 26) were more frequently localised in the northern (Skellefteå) region (P = 0.0050, Fisher's exact test), whereas those with *cbm* infection (n = 7) were localised only in the southern (Umeå) region (Fig. 3b, Table S8). We could not localise the more prevalent SpaP A and B types or children who were or were not infected to different geographic locations.

### Unstable residency and mixed fluctuating SpaP ABC phenotypes specify high cariogenic potential and stable SpaP A residency specifies low cariogenic potential

To explore phenotypes and mechanisms that specify high cariogenic and virulence potential of the mixed and dominant SpaP A/B/C types, we explored the residencies of single dominant and mixed SpaP A/B/C and Cnm/Cbm adhesin types related to caries progression in 452 adolescents at the ages of 12 and 17 years (Table 4, Tables S6, S7, S9, S10 and S11). Most adolescents (78%) fell into the persistent infection (+, +) or non-infection (–, –) groups. Regardless of *S. mutans per se* and dominant or mixed adhesin types, caries progression occurred in the following order: persistent infection (+, +) > gain of infection (–, +) > loss of infection (+, –) > persistent non-infection (–, –) (Table 4, Tables S6, S7 and S9).

The changing group (22%) had temporal changes in terms of gain (–, +) or loss (+, –) of infection or a mixed fluctuating mode that switched from a mixed to a dominant SpaP A/B/C type or vice versa (*i.e.*, mix-dom, dom-mix, mix-mix). Adolescents infected with a mixed fluctuating SpaP ABC mode experienced more caries and progression than those infected with dominant SpaP B or SpaP A types, whether analysed combined or separately (Table 4, Tables S6, S7 and S9). Host caries activity accordingly coincided inversely with the stability of infection for SpaP mix–mix (37%), B–B (51%), and A–A (80%) (Table 4, Tables S6, S7 and S9). The mixed SpaP ABC type—typically AB, AC, or BC but rarely ABC—frequently shifted into SpaP A or into another type rather than being lost (Fig. 3a). The stable SpaP A type, on the other hand, was more frequently gained than lost (Fig. 3a), and its low cariogenic potential relative to other adhesion types also remained at a high infection load (Table 4, Tables S9, S10 and S11).

### The SpaP B-1 subtype accounts for a major portion of biotype B cariogenicity

We next examined the cariogenic potential of the SpaP B-1 subtype in the full group of 452 adolescents (Table 4 and Table S9). Infection with the SpaP B-1 type (7% prevalence) coincided with a 1.7-fold increased caries progression compared with SpaP B non-B-1 types in the entire cohort. This finding verifies that the B-1 subtype accounts for a major portion of SpaP B cariogenicity. Moreover, B-1 (7 of 28, P = 0.025 Fisher's exact test) and Cnm (9 of 26, P = 0.00089, Fisher's exact test) were overrepresented in adolescents infected (n = 50) versus not infected (n = 402) with mixed SpaP ABC types that coincided with somewhat increased numbers of *S. mutans* and proportions of total oral streptococci (Tables S6 and S7, Fig. S1). Stable B–B residency, on the other hand, coincided with a comparatively low cariogenicity (Table 4 and Table S9).

### Cnm expression and glycosylation type influence host caries activity and saliva pattern recognition

**Fig. 4:**
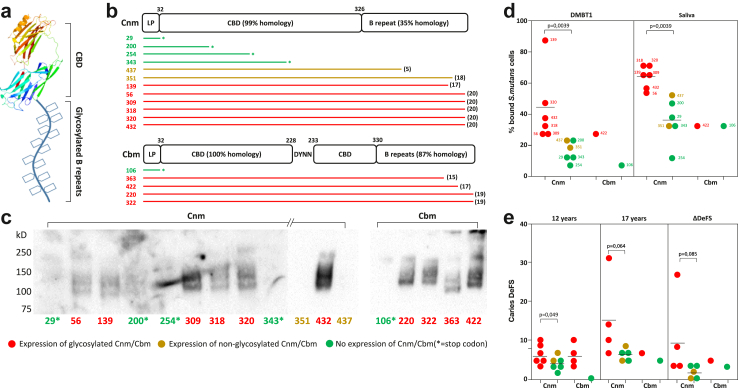
**Loss of Cnm infection and lack of expression of glycosylation reduce caries activity and pattern recognition**. (**a**) Molecular model of Cnm/Cbm with collagen-binding domain (CBD) and glycosylated B repeats. (**b**) Wild-type full-length and truncated Cnm and Cbm protein structures in wild-type isolates from 452 adolescents. Truncated protein structures harbour a stop codon before or in the CBD (marked with green/star). The number of B-repeats in full-length Cnm and Cbm proteins is shown in parentheses. (**c**) Detection of glycosylation of Cnm/Cbm in cell wall extracts from 12 Cnm to 5 Cbm strains using a glycosylation kit (and antisera against Cnm/Cbm) in western blots. Strains with a stop codon in CBD (strains 29, 200, 245–343, and 106, green) and two Cnm strains (351 and 437, orange) with shorter B-repeat domains lacked glycosylation. Results are from three separate membranes. Results were replicated at least twice. (**d, e**) Binding and host caries progression was associated with Cnm/Cbm expression and glycosylation. Both expression and glycosylation of Cnm strains coincided with significantly higher DMBT1 and saliva binding (P = 0.0039 and 0.0039, Mann–Whitney U tests) and host caries experience (age 12 y) and progression (age 17 y, ΔDeFS) compared with non-expressed or non-glycosylated Cnm strains; see also Table S12, Figs. S2 and S3. The corresponding P-values for DMBT1 and saliva binding of Cnm/Cbm combined were P = 0.0017 and P = 0.0060, respectively (Mann–Whitney U tests).

Loss (+,–) and gain (–,+) of *cnm* infection during adolescence coincided with markedly lower caries progression compared with what was seen in stably (+,+) infected children (ΔDeFS 1.3 and 2.3 versus ΔDeFS 6.9) (Tables S6 and S7). We therefore hypothesised that expression and glycosylation of the Cnm adhesin in wild-type isolates could influence caries activity and pattern recognition in the individual strain donors (Fig. 4, Table S12).

Wild-type Cnm (n = 12) and Cbm (n = 5) isolates were analysed for expression and glycosylation of Cnm and Cbm related to caries and saliva pattern recognition/binding. Expression of Cnm and Cbm was absent in four *cnm-* and one *cbm-*positive isolates/individuals, as inferred from translational stop codons in the *cnm/cbm* genes and western blot experiments with membrane extracts probed with Cnm and Cbm antisera and detection of glycosylation (Fig. 4 and Fig. S2). The six isolates/individuals associated with glycosylated Cnm protein had higher binding to saliva (P = 0.0039) and DMBT1 (P = 0.0039) and ∼2-fold more baseline caries than with non-expressing isolates (mean DeFS 6.3 vs 4.2, P = 0.049) (Mann–Whitney U tests) (Fig. 4). The same trend difference remained for caries at age 17 years and for the 5-year caries increment.

Examination of the glycosylation profiles of *cnm*- and *cbm*-positive isolates revealed that those with translational stop codons, but also isolate 437 with a short B-repeat sequence (5 repeats) and isolate 351, all lacked glycosylated protein bands (Fig. 4 and Fig. S3). Among Cnm-expressing isolates, the two non-glycosylated but Cnm-expressing phenotypes 437 and 351 from two participants coincided with the lowest DMBT1 and saliva pattern recognition and 5-year caries increment (Fig. 4 and Fig. S2, Table S12).

### Adolescents with markedly high *S. mutans* numbers and caries levels during adolescence show mixed/fluctuating SpaP B-1 or P4a susceptibility profiles

We hypothesised that the human and *S. mutans* phenotypes may be more important in caries progression than is the quantity of clinical risk markers such as *S. mutans* levels. We therefore examined those adolescents with markedly high *S. mutans* levels at baseline alone and at 17 years of age in association with caries and infection with high-cariogenicity mixed or B-1 types and human *PRH1*, *PRH2* susceptible or resistant phenotypes (Tables 4 and 5 and Table S9). Adolescents with markedly high *S. mutans* numbers at baseline alone (21/452) and also at 17 years of age (3/390) had 1.7- and 7-fold increased caries progression, respectively, compared with children without infection (Tables 4 and 5 and Table S9). Two of the three extreme cases were infected with the mixed, fluctuating SpaP mode (mixed, B-1), and the third had a susceptible P4a phenotype. In addition, based on questionnaire responses, we could not link the three extremes to markedly deviating oral care or lifestyle habits (Table 5).

**Table 5:** Characteristics of 3 extremes stable colonized with high numbers of *S. mutans*.

Character	Adolescent 1	Adolescent 2	Adolescent 3
Sex	Boy	Girl	Boy
*S. mutans* counts^a^			
12 y	896,550	1,676,340	750,600
17 y	2,051,265	427,342	384,557
Caries			
DeFS 12 y^b^	8	0	9
DeFS 17 y^b^	41	8	27
ΔDeFS^c^	38.5	5.9	18.1
Lifestyle^d^			
Toothbrushing			
12 y	Twice/day	Irregularly	Twice/day
17 y	Twice/day	Once/day	Twice/day
Sweets			
12 y	Several times/week	Several times/week	Once/week
17 y	Several times/week	Once/week	Several times/week
Sugary drinks			
12 y	Once/week	Several times/week	Once/day
17 y	Several times/week	Once/day	Once/week
Extra fluoride			
12 y	No	No	No
17 y	No	No	No
*S. mutans* type			
SpaP type			
12 y	B (B-1)	A	Mixed B (B-1)/C
17 y	Mixed B/C	A	B
Genetics			
*PRH1/2* type	–	P4a	–

a *S. mutans* counts in whole saliva measured by qPCR.

b Caries DeFS (Decayed, enamel included, Filled Surfaces) at 12 and 17 years of age.

c ΔDeFS (5 y) = 5-year prospective caries increment from 12 to 17 years of age.

d Lifestyle data collected by questionnaire at 12 and 17 years of age.

Moreover, in the entire cohort, we could not link lifestyle in terms of oral hygiene and frequency of sugar intake to numbers, adhesion type/mode, or temporal changes in *S. mutans* in multivariate PLS (R^2^ = 0.074, Q^2^ = −0.009) or traditional statistical analyses (Table S13); the sole exception was an increased gain in *S. mutans* with poor oral hygiene. In addition, bacterial numbers showed a skewed distribution and generally varied greatly at long (12 and 17 years) and short (4–6 weeks) time scales (Fig. S4).

### *In vitro* and *in situ* properties of the SpaP A/B/C and Cnm/Cbm adhesion types related to cariogenicity and virulence

We next investigated a representative isolate for each SpaP A/B/C and Cnm/Cbm adhesion type for caries- and virulence-related mechanisms. We explored binding of the isolates to oral mucosal tissues *in situ* and to collagen types I–VI and DMBT1 *in vitro* and for saliva-mediated biofilm formation under aerobic/anaerobic and acidic conditions (Fig. 3, Table S14).

The Cnm and Cbm isolates bound strongly to the subepithelial but not the epithelial compartment of buccal mucosal tissue sections *in situ* (Fig. 3c, Table S14). In contrast, neither SpaP A/B/C isolates nor Cnm knockout mutants bound to the subepithelial or epithelial compartments. Moreover, the Cnm and Cbm isolates (and recombinant Cnm/Cbm) bound strongly to collagen types I–V and DMBT1 but not to collagen type VI, casein, or albumin (Fig. 3d, Table S14). SpaP A/B/C strains bound weakly, if at all, to collagens I–VI but strongly to DMBT1.

Biofilm-forming ability on saliva-coated surfaces was in the following order: Cnm 56 (9.3%) showing high-density areas > Cbm, SpaP B and C > SpaP A 98 (1.6%) showing no high-density areas (Fig. 3e and f, Table S14). Moreover, all SpaP A/B/C and Cnm/Cbm types showed moderate to high acid tolerance in aerobic and anaerobic conditions after adaptation, whereas only SpaP A and B had moderate acid tolerance in aerobic conditions without adaptation (Table S14).

### Prevalence of *S. mutans* SpaP A/B/C and Cnm/Cbm adhesion types during adolescence

The prevalence of *S. mutans per se* increased from 48% to 55% from 12 to 17 years of age (P = 0.027, Fisher's exact test) (Table S15). During this time, the prevalences of SpaP A, SpaP C, and mixed SpaP A/B/C types also increased, whereas high-cariogenicity SpaP B decreased from 16% to 10% between these two ages (P = 0.018, Fisher's exact test).

## Discussion

The results of this study suggest that *PRH1*, *PRH2* receptor phenotypes may dictate the repertoire of and sensitivity to pioneer commensal and *S. mutans* microbiota adhering to and colonising teeth in low-caries populations. Caries progression in resistant P1 individuals seemed to require flora with high-virulence *S. mutans* phenotypes, which are mixed fluctuating SpaP ABC, B-1 subtype, and Cnm expression/glycosylation phenotypes. By contrast, highly (P4a) and moderately (P6) susceptible individuals developed caries from a broader microbial flora that included moderately cariogenic oral commensal streptococci and lactobacilli. We hypothesise that the influence of the host genome on the microbiota and innate defences is a primary cause of caries in populations with a low caries prevalence and physiological (lifestyle) homogeneity. The unique character of each host–microbe interaction appears evident from the specificity (tropism) of *S. mutans* SpaP A/B/C for individual hosts and the distribution of the high-virulence phenotypes in a smaller set of adolescents. However, superimposed on these associations are lifestyle- and sucrose-dependent mechanisms that are primary causes of caries progression and polymicrobial shifts in high-caries populations with physiological heterogeneity. Each of the long-lasting monomicrobial (specific) and polymicrobial (unspecific and ecological) plaque hypotheses may accordingly be valid, depending on the individual and population.

*PRH1* and *PRH2*, which encode salivary acidic PRPs, specify the major high- (P4a), moderate- (P6), and low (P1)-caries phenotypes that in turn reflect different causal saliva immunodeficiency (P4a) and lifestyle (P1) subtypes of caries.^11^ The acidic PRPs are primary receptors for oral commensal streptococci and actinomycetes and co-receptors for DMBT1-mediated adhesion of *S. mutans* on tooth surfaces, where they exert barrier and other innate immunity functions. It is therefore not surprising that the *PRH1*, *PRH2* phenotypes seem to be associated with caries development from a narrow, monomicrobial (P1) to a broad (P6) and even broader (P4a) polymicrobial profile of oral commensal streptococci and lactobacilli. The *PRH1*, *PRH2* saliva receptor repertoires may dictate both the adhesion and colonisation profiles of oral pioneer streptococci–actinomycetes and lactobacilli communities and host susceptibility to this microbial challenge in terms of immunity barriers and responses. The immunodeficiency P4a and P6 phenotypes may accordingly also select for and support development of caries from the abundant non-mutans streptococci, such as *S. oralis* and *S. mitis* biovar 1, and lactobacilli of moderate acid tolerance and production. P1 phenotypes, in particular in individuals who have never experienced caries, may select core commensal biofilm members of markedly low cariogenicity and harbour resistance traits, requiring infection with *S. mutans* high-cariogenicity phenotypes and poor lifestyle habits for caries progression. However, the small panel of bacterial species analysed limits our conclusions. We are accordingly undertaking functional analyses of biofilm short chain fatty acids (SCFA) and metagenomic analyses of the saliva microbiome, including whole-genome sequencing of *S. mutans* isolates, from the adolescent participants to explore possible microbial oro-types of the P4a, P6, and P1 and *S. mutans* SpaP A/B/C and Cnm/Cbm phenotypes.

Our results also suggest that the *S. mutans* adhesion types exhibit tropism (specificity) for individual hosts and isoreceptors, emphasising that the human genetic endowment also could determine the initial adhesion and colonisation profile of *S. mutans* phenotypes. Indeed, residency of *S. mutans* adhesion types was generally stable over time and virtually free of switching from one to another dominant SpaP A/B/C or Cnm/Cbm adhesion type in children, despite adolescence and puberty. The different geographic localisation of infection with Cnm and Cbm phenotypes of comparably low prevalence could even mean that *S. mutans* adhesion types—and plausibly even long-time stable lineages/clonal complexes—disseminate within families.^42^ Reminiscent of host tropism and infection susceptibility in the cases of uropathogenic *Escherichia coli*,^43^ the gastric pathogen *Helicobacter pylori*,^44^ and noro/HIV viruses,^14^ these findings suggest a close fit between the structurally distinct SpaP A/B/C receptor-binding domains and their saliva DMBT1 and biofilm isoreceptors.^43^ Considering that the SpaP (AgI/II) adhesin family is typical of oral streptococci and their bacteria–bacteria interactions, the SpaP A/B/C phenotypes may form pioneer, inter-generic streptococcal communities with different acidic microenvironments and cariogenic potentials.^45^

The present findings suggest a wide range of low-to high-virulence phenotypes of *S. mutans* that may explain the variable penetrance of disease in individuals infected with this “commensal pathogen”.^46^ In a few adolescents, high virulence involved fluctuating mixed SpaP ABC modes, B-1, and Cnm expression/glycosylation phenotypes and unstable residency. Indeed, residency was unstable for B-1 subtypes, and the fluctuating mixed SpaP ABC mode, commonly AB but rarely ABC, frequently transformed into single stable SpaP A types, plausibly as a consequence of competition for space, nutrients, and receptor sites. We do not know the mechanisms of this dysbiosis or if a subset of individuals had a “dual” receptor repertoire selecting for competing SpaP A/B/C types. Regardless, the high-cariogenicity B-1 and Cnm phenotypes seemed overrepresented in the fluctuating mode. In contrast, the SpaP A type of comparably low cariogenicity was highly prevalent and stable (persistent) and may have contributed to asymptomatic *S. mutans* infection in many individuals. In this respect, the SpaP A type reminds of the moderate cariogenic but abundant and commensal *S. mitis* biovar 1 and *S. oralis*.^24^ *S. mutans* biotypes A and B differed in cariogenic mechanisms, such as pattern recognition and acid tolerance, and in cariogenic potential *in vivo*, regardless of infection load. However, it remains to be determined whether such phenotypic differences are attenuated or more pronounced in populations with a high caries prevalence driven by general sucrose-dependent biofilm acidity and cariogenicity.

Interestingly, our findings show that Cnm expression and glycosylation in wild-type isolates influenced caries activity and saliva/DMBT1 binding. Accordingly, in a few isolates/human donors, caries and receptor binding activity were reduced in the presence of a complete lack of Cnm expression (n = 4) and O-glycosylation in Cnm-expressing (n = 2) bacteria. Cnm expression may be linked to a regulator affecting the overall Cnm phenotype, and the protein and sugar moieties may exert direct cariogenic effects. The binding avidity of Cnm and SpaP B phenotypes to collagen and DMBT1 correlates with individual caries activity.^12^ In addition, Cnm phenotypes bind strongly to subepithelial tissue sections *in situ*, and recombinant Cnm can block wound healing in an animal stroke model.^31^ Similar to the glycotype of GspB in *Streptococcus gordonii*, the Cnm glycotype may influence tissue binding and invasion and DMBT1-mediated pattern recognition and immunity. Of note, the small DMBT1 caries susceptibility allele I is associated with the loss of binding of a Cnm strain and *FUT2* fucose glycosylation.^47^ We hypothesise that the Cnm phenotype may change SpaP and Cnm expression and glycotype depending on local and systemic niches and conditions during infection.

The load of oral commensal streptococci increased the 5-year caries increment but not the baseline caries experience in the entire cohort of adolescents. This finding is intriguing but consistent with a narrow-to-broad microbiota dependency of the *PRH1*, *PRH2* phenotypes. It also could indicate age-related successions in the oral commensal streptococcal flora of species with low and moderate cariogenicity, or immunity changes during puberty and adolescence, as has been reported.^48^^,^^49^ Of note, the acidic, basic, and glycosylated PRPs encoded by the *PRH1*, *PRH2*, and *PRB1-4* loci display profound age-dependent differences in expression and post-translational modifications during adolescence and the development of the permanent dentition.^50^, ^51^, ^52^ Such changes and the present finding that low-cariogenicity SpaP A prevalence increased while high-cariogenicity SpaP B prevalence decreased during puberty may help to explain why caries attenuates in late adolescence and adulthood. The qualitative nature of caries progression also was supported by the finding that the three adolescents with markedly high and stable numbers of *S. mutans* and caries lesions during adolescence were characterised by mixed fluctuating SpaP B-1 (n = 2) and host P4a susceptibility (n = 1) phenotypes.

A strength of our prospective study design is that it allowed us to use 5-year caries ΔDeFS increment/incidence and DeFS scores at age 17 years to pinpoint baseline predictors of caries and plausible causal or virulence factors in a population of low caries prevalence. Infection with B-1 (6.2%), Cnm (5.8%), and the fluctuating mixed SpaP A/B/C mode (8.8%) defined the high-risk group (15%) better than *S. mutans* infection *per se* (48%) or gross load of infection. In addition, adolescents with markedly high *S. mutans* levels at baseline (>90% percentile, n = 21) had lower caries progression than adolescents infected with *cnm* at baseline (n = 26). We are currently evaluating the classification and prevention of caries based on *PRH1*, *PRH2* genetic P4a^+^ versus P4a^–^ risk and cause in terms of health economy [unpublished, Strömberg et al.]. However, the influence of Cnm phenotype expression and glycosylation on individual caries activity emphasises the unique character of each host–microbe interaction. Individual risk estimation in terms of absolute sensitivity and specificity values will accordingly require extensive host genetic, microbiota, and exposome biomarker analysis and further delineation of susceptibility or virulence genes and pathways. Another strength of the study design is that it allowed use of single dominant isolates from strain donors to match strain glycosylation and receptor binding to individual caries activity. Future diagnostics or mechanistic approaches thus may require whole-genome sequencing of both isolates and caries-free and affected cases in families. Isolates and host tissue samples may then be explored for misbehaving molecular mechanisms and pathways. This knowledge will allow for improved diagnostics, prevention, and treatment approaches in personalised dentistry, adjusted to populations of low versus high caries prevalence. This individualisation could be developed and applied for children, adults, and elderly patients or in the perinatal and infancy periods when the human genetic complement synergises with the exposome to shape the microbiota and human phenotype.^53^

In summary, our results suggest that individuals who are genetically resistant to caries develop caries from microflora with *S. mutans* high-virulence mixed/fluctuating SpaP ABC, B-1, or Cnm expression/glycosylation phenotypes. Highly and moderately genetically susceptible individuals, on the other hand, seem to develop caries from a broader microflora, including moderate cariogenic oral commensal streptococci and lactobacilli. Our findings also emphasise that each host–microbe interaction is unique and that genetic rather than lifestyle factors may be the primary causal agents of caries in populations with low caries prevalence. In this sense, risk classification models based on genetic, microbiota, and lifestyle biomarkers should be adjusted to the type of population. Fine-tuned personalised risk assessment will require detailed information about the unique character of each host–microbe and host–exposome interaction.

## Contributors

Conceptualisation: NS, NOS.

Methodology: All.

Investigation: All.

Visualisation: NS, NOS.

Data curation, formal analysis; NS, NOS.

Funding acquisition: NS, AW, LM, GS.

Writing—original draft: NS, NOS.

Writing—review and editing: All.

Verification of underlying data: NS, NOS, GS.

All authors read and approved the final version of the manuscript.

## Data sharing statement

All data are available in the main text and supplementary materials. Correspondence and requests for materials should be addressed to Nicklas Strömberg. The *cbm* nucleotide sequence data were deposited at GenBank https://www.ncbi.nlm.nih.gov/genbank/ with accession numbers OR596380 through OR596384 for strains 106, 220, 322, 363, and 422, respectively.

## Declaration of interests

Authors declare that they have no competing interests.

## Appendix A Supplementary data

Full blots
