# Supplementary material for: Human PRH1, PRH2 susceptibility and resistance and Streptococcus mutans virulence phenotypes specify different microbial profiles in caries
Source: eBioMedicine. 2024 Feb 15;101:105001. doi: 10.1016/j.ebiom.2024.105001 (PMC10878843; doi:10.1016/j.ebiom.2024.105001)

**Full membranes for western blots**

**Glycosylation of Cnm and Cbm proteins (Figure 4c and Figure S2b)**


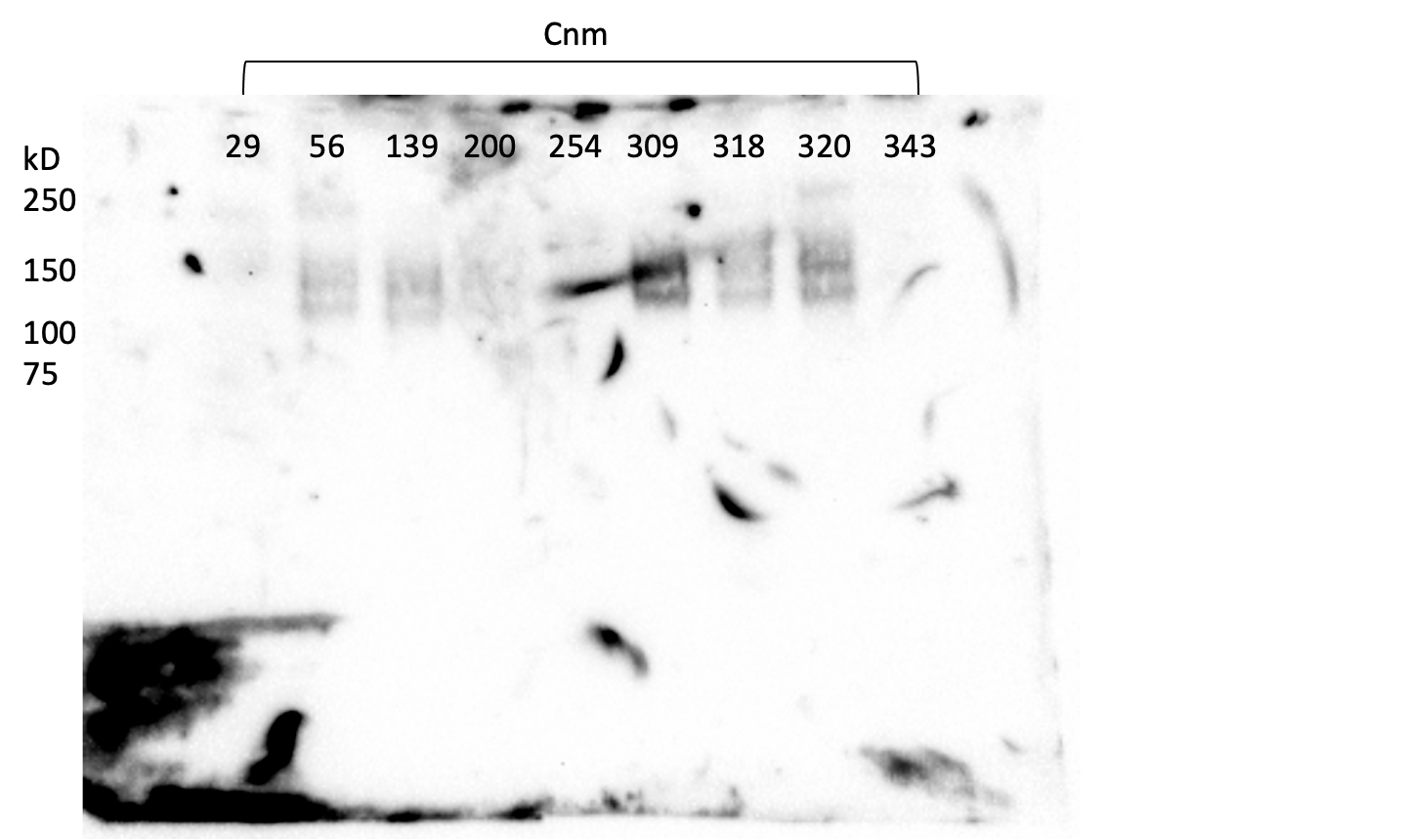


**Glycosylation of Cnm and Cbm proteins (Figure 4c and Figure S2b)**


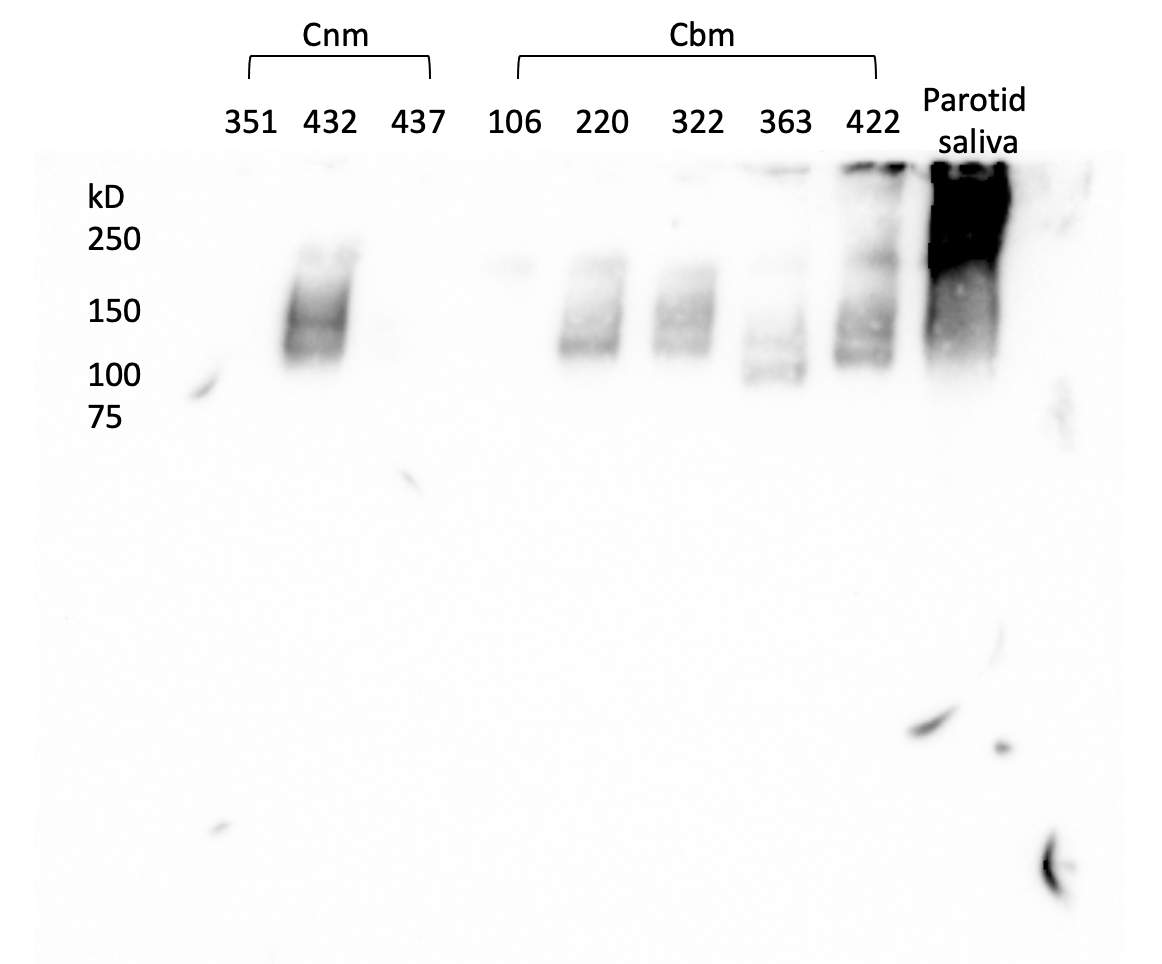


**Expression of Cnm proteins (Figure S2a)**


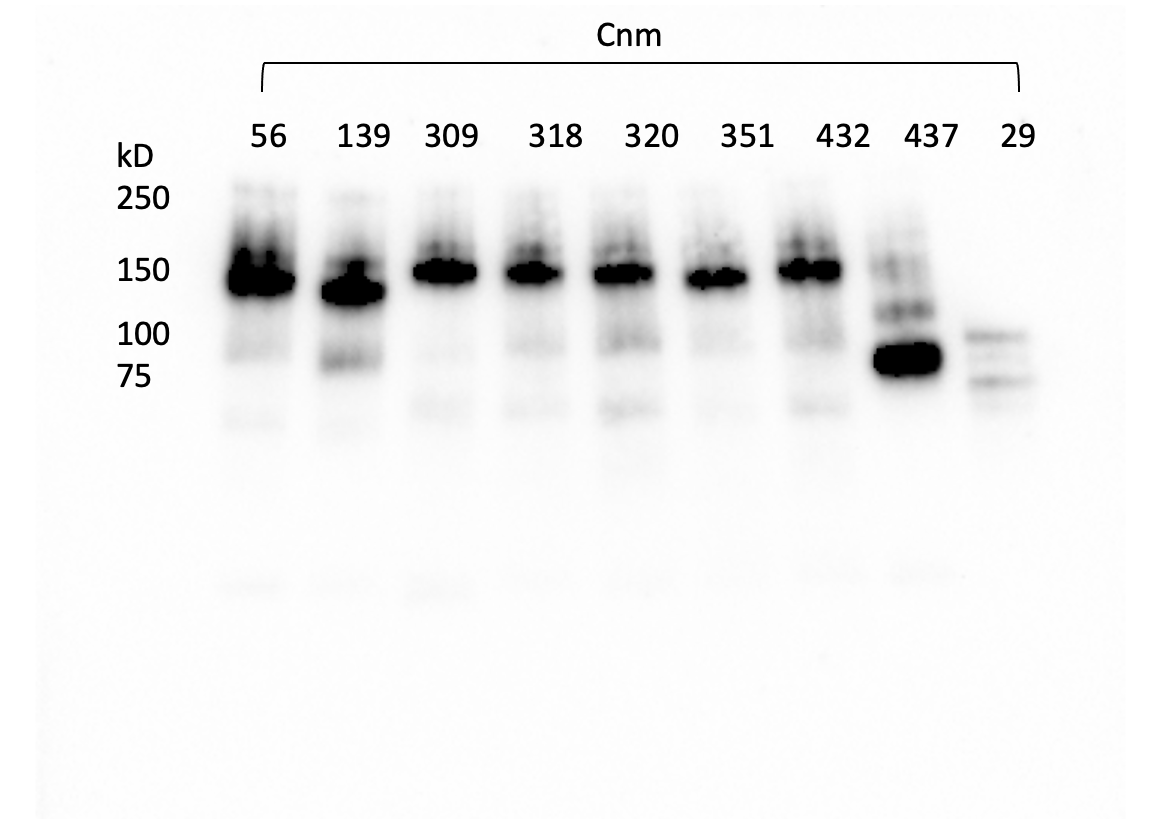


**Expression of Cnm proteins (Figure S2a)**


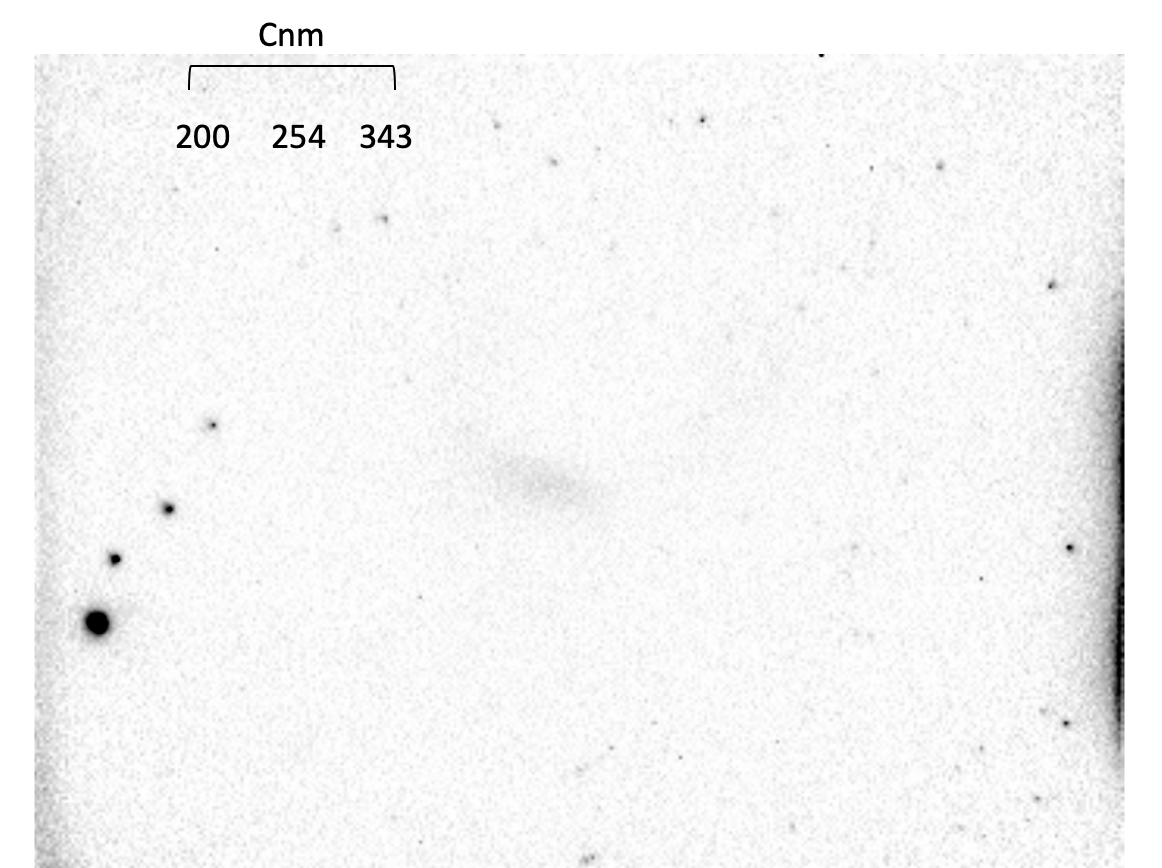


**Expression of Cbm proteins (Figure S2a)**


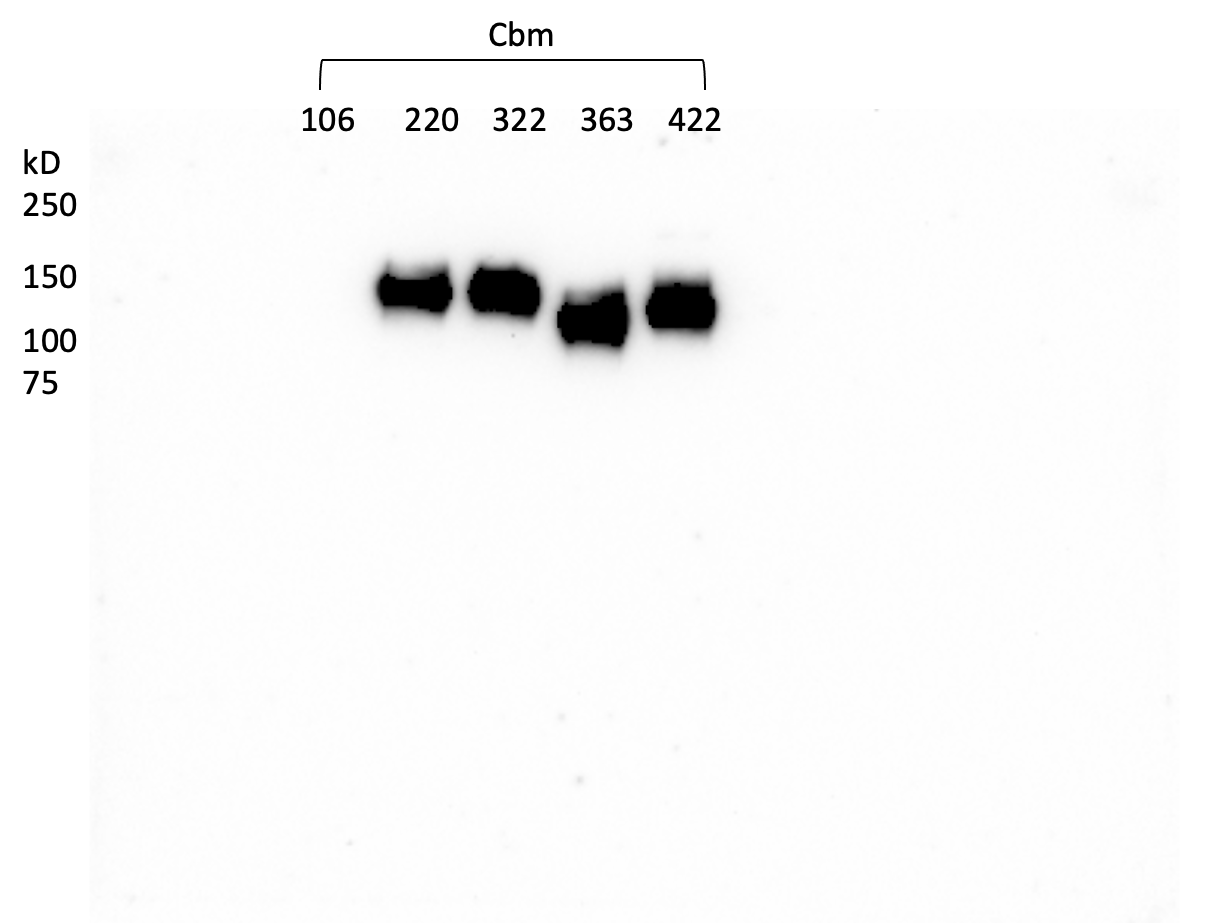

Supplement: Full blots [file mmc1.docx]
