# Supplementary material for: Human PRH1, PRH2 susceptibility and resistance and Streptococcus mutans virulence phenotypes specify different microbial profiles in caries
Source: eBioMedicine. 2024 Feb 15;101:105001. doi: 10.1016/j.ebiom.2024.105001 (PMC10878843; doi:10.1016/j.ebiom.2024.105001)
Supplement: Supplementary file 2 — Tables S1–S15 and Figures S1–S4 [file mmc2.docx]

**Supplemental material**

Table of contents

[**Table S1** 2](#_Toc154056292)

[**Table S2** 3](#_Toc154056293)

[**Table S3** 4](#_Toc154056294)

[**Table S4** 5](#_Toc154056295)

[**Table S5** 6](#_Toc154056296)

[**Table S6** 7](#_Toc154056297)

[**Table S7** 8](#_Toc154056298)

[**Table S8** 9](#_Toc154056299)

[**Table S9** 10](#_Toc154056300)

[**Table S10** 11](#_Toc154056301)

[**Table S11** 12](#_Toc154056302)

[**Table S12** 13](#_Toc154056303)

[**Table S13** 14](#_Toc154056304)

[**Table S14** 15](#_Toc154056305)

[**Table S15** 16](#_Toc154056306)

[**Fig. S1** 17](#_Toc154056307)

[**Fig. S2** 19](#_Toc154056308)

[**Fig. S3** 20](#_Toc154056309)

[**Fig. S4** 21](#_Toc154056310)

| **Table S1**. Influence of *S. mutans* SpaP A/B/C adhesion types on caries progression in high (P4a), moderate (P6), and low (P1) caries genotypes and phenotypes | | | | | | | | | |
| --- | --- | --- | --- | --- | --- | --- | --- | --- | --- |
| Type | DeFS-12y^b^ | | |  | DeFS-17y^b^ | |  | ΔDeFS-5y^c^ | |
|  | n | median (IQR) | P^d^ | n | median (IQR) | P^d^ |  | median (IQR) | P^d^ |
| P4a |  |  |  |  |  |  |  |  |  |
| SpaP A | 19 | 3.0 (2.0, 6.0) | 0.075 | 16 | 6.0 (2.0, 14.5) | 0.51 |  | 1.7 (0.0, 11.0) | 0.61 |
| SpaP B | 11 | 3.0 (1.0, 7.0) | 0.13 | 9 | 9.0 (6.0, 13.5) | 0.025 |  | 4.2 (3.1, 8.6) | 0.065 |
| SpaP C | 2 | 3.5 (-) | 0.34 | 2 | 8.5 (-) | 0.35 |  | 5.3 (-) | 0.48 |
| SpaP neg | 42 | 2.0 (0.0, 4.0) | Ref. | 37 | 5.0 (2.0, 10.0) | Ref. |  | 2.5 (0.0, 7.1) | Ref. |
|  |  |  |  |  |  |  |  |  |  |
| P6 |  |  |  |  |  |  |  |  |  |
| SpaP A | 25 | 3.0 (0.0, 5.0) | 0.0077 | 24 | 7.0 (1.3, 10.8) | 0.033 |  | 2.6 (0.9, 8.2) | 0.11 |
| SpaP B | 17 | 4.0 (1.5, 5.5) | 0.0013 | 11 | 8.0 (6.0, 10.0) | 0.0044 |  | 4.1 (0.9, 8.0) | 0.11 |
| SpaP C | 4 | 3.5 (0.8, 5.5) | 0.11 | 4 | 6.5 (1.5, 13.8) | 0.29 |  | 1.3 (0.2, 9.1) | 0.84 |
| SpaP neg | 56 | 0.5 (0.0, 3.0) | Ref. | 54 | 3.0 (1.0, 6.3) | Ref. |  | 1.6 (0.0, 4.0) | Ref. |
|  |  |  |  |  |  |  |  |  |  |
| P1 |  |  |  |  |  |  |  |  |  |
| SpaP A | 23 | 2.0 (0.0, 3.0) | 0.42 | 21 | 4.0 (1.0, 8.0) | 0.47 |  | 2.4 (0.0, 5.9) | 0.40 |
| SpaP B | 17 | 2.0 (0.0, 5.0) | 0.067 | 14 | 7.0 (3.0, 9.8) | 0.013 |  | 3.0 (1.5, 5.3) | 0.048 |
| SpaP C | 6 | 3.0 (1.5, 5.0) | 0.18 | 5 | 4.0 (1.5, 7.5) | 0.95 |  | 1.1 (0.5, 3.5) | 0.60 |
| SpaP neg | 69 | 1.0 (0.0, 2.5) | Ref. | 56 | 3.0 (1.0, 5.0) | Ref. |  | 1.7 (0.0, 3.3) | Ref. |
| ^a^ High (P4a), moderate (P6), and low (P1) caries phenotypes with Swedish ethnicity defined by *PRH1*, *PRH2* genetic variation. | | | | | | | | | |
| ^b^ Caries DeFS (Decayed, enamel-included, Filled Surfaces) at 12 and 17 years of age. | | | | | | | | | |
| ^c^ ΔDeFS (5y) = 5-year prospective caries increment from 12 to 17 years of age. | | | | | | | | | |
| ^d^ 2-sided P value from Mann-Whitney U test. | | | | | | | | | |

| **Table S2**. Influence of load of *S. mutans*, lactobacilli, and streptococci on caries progression in the high (P4a), moderate (P6), and low (P1) caries phenotypes defined by *PRH1*, *PRH2* genetic variation | | | | | | | | | | |
| --- | --- | --- | --- | --- | --- | --- | --- | --- | --- | --- |
| Types | Load |  | DeFS-12y^b^ | |  | DeFS-17y^b^ | |  | ΔDeFS-5y^c^ | |
|  |  | n | median (IQR) | P^d^ | n | median (IQR) | P^d^ |  | median (IQR) | P^d^ |
| *S. mutans*^e,f^ | |  |  |  |  |  |  |  |  |  |
| P4a | Negative | 34 | 2.0 (0.0, 4.0) | Ref. | 30 | 4.0 (1.0, 11.0) | Ref. |  | 2.1 (0.0, 8.0) | Ref. |
|  | Low | 21 | 3.0 (1.5, 5.0) | 0.086 | 16 | 5.0 (3.0, 10.0) | 0.23 |  | 3.2 (1.1, 4.3) | 0.84 |
|  | High | 20 | 2.5 (1.3, 7.0) | 0.033 | 19 | 8.0 (5.0, 12.0) | 0.061 |  | 3.4 (1.7, 9.7) | 0.25 |
|  |  |  |  |  |  |  |  |  |  |  |
| P6 | Negative | 55 | 0.0 (0.0, 3.0) | Ref. | 51 | 3.0 (1.0, 6.0) | Ref. |  | 1.2 (0.0, 4.0) | Ref. |
|  | Low^f^ | 21 | 2.0 (0.0, 3.0) | 0.31 | 19 | 3.0 (0.0, 13.0) | 0.56 |  | 1.7 (0.0, 7.7) | 0.34 |
|  | High^f^ | 26 | 5.0 (2.0, 7.0) | <0.0001 | 23 | 10.0 (7.0, 10.0) | <0.0001 |  | 4.1 (1.1, 6.9) | 0.0024 |
|  |  |  |  |  |  |  |  |  |  |  |
| P1 | Negative | 61 | 1.0 (0.0, 2.0) | Ref. | 50 | 3.0 (0.8, 5.0) | Ref. |  | 1.7 (0.0, 3.3) | Ref. |
|  | Low^f^ | 26 | 1.0 (0.0, 4.0) | 0.25 | 21 | 3.0 (1.5, 7.5) | 0.64 |  | 1.7 (0.0, 4.2) | 0.84 |
|  | High^f^ | 28 | 2.0 (0.3, 5.0) | 0.0069 | 25 | 6.0 (3.0, 9.5) | 0.0041 |  | 3.3 (1.2, 5.5) | 0.024 |
|  | |  |  |  |  |  |  |  |  |  |
| Lactobacilli^e,g^ | |  |  |  |  |  |  |  |  |  |
| P4a | Negative | 36 | 2.0 (0.0, 4.0) | Ref. | 33 | 4.0 (1.5, 10.0) | Ref. |  | 2.0 (0.0, 4.2) | Ref. |
|  | Low | 23 | 3.0 (1.0, 5.0) | 0.46 | 18 | 6.0 (3.8, 10.0) | 0.18 |  | 3.2 (1.1, 6.2) | 0.37 |
|  | High | 16 | 4.5 (1.0, 5.0) | 0.27 | 14 | 8.0 (4.0, 22.8) | 0.019 |  | 7.1 (1.3, 15.2) | 0.062 |
|  |  |  |  |  |  |  |  |  |  |  |
| P6 | Negative | 54 | 1.0 (0.0, 3.0) | Ref. | 49 | 3.0 (0.0, 7.0) | Ref. |  | 1.1 (0.0, 3.7) | Ref. |
|  | Low | 28 | 2.0 (0.0, 4.8) | 0.29 | 26 | 4.0 (1.0, 10.3) | 0.093 |  | 2.9 (0.7, 6.1) | 0.078 |
|  | High | 20 | 4.0 (1.5, 4.0) | 0.0015 | 18 | 7.0 (5.8, 11.5) | 0.00047 |  | 3.7 (1.6, 8.6) | 0.0060 |
|  |  |  |  |  |  |  |  |  |  |  |
| P1 | Negative | 68 | 0.5 (0.0, 3.0) | Ref. | 55 | 3.0 (1.0, 6.0) | Ref. |  | 1.7 (0.0, 3.6) | Ref. |
|  | Low | 31 | 2.0 (0.0, 3.0) | 0.33 | 27 | 3.0 (0.0, 7.0) | 0.90 |  | 2.4 (0.0, 4.8) | 0.98 |
|  | High | 16 | 2.5 (1.3, 4.8) | 0.0037 | 14 | 5.0 (2.8, 8.3) | 0.077 |  | 1.9 (1.0, 3.7) | 0.63 |
|  | |  |  |  |  |  |  |  |  |  |
| Streptococci^e,h^ | |  |  |  |  |  |  |  |  |  |
| P4a | Low | 21 | 3.0 (0.5, 4.0) | Ref. | 18 | 4.0 (2.8, 6.8) | Ref. |  | 1.9 (0.0, 4.1) | Ref. |
|  | Moderate | 26 | 2.5 (0.0, 5.0) | 0.67 | 24 | 5.0 (2.0, 12.0) | 0.52 |  | 3.4 (0.2, 8.7) | 0.20 |
|  | High | 28 | 2.0 (0.0, 6.0) | 0.85 | 23 | 8.0 (5.0, 10.0) | 0.056 |  | 2.9 (1.0, 7.6) | 0.25 |
|  |  |  |  |  |  |  |  |  |  |  |
| P6 | Low | 29 | 2.0 (0.0, 4.0) | Ref. | 27 | 2.0 (1.0, 7.0) | Ref. |  | 1.1 (0.0, 3.3) | Ref. |
|  | Moderate | 36 | 2.0 (0.0, 4.0) | 0.48 | 32 | 7.0 (3.0, 10.8) | 0.014 |  | 3.9 (0.8, 7.7) | 0.019 |
|  | High | 37 | 2.0 (0.0, 4.0) | 0.56 | 34 | 5.0 (1.0, 7.8) | 0.20 |  | 2.0 (0.6, 4.9) | 0.18 |
|  |  |  |  |  |  |  |  |  |  |  |
| P1 | Low | 19 | 2.0 (0.0, 4.0) | Ref. | 15 | 3.0 (1.0, 5.0) | Ref. |  | 1.0 (0.0, 2.6) | Ref. |
|  | Moderate | 56 | 1.5 (0.0, 3.0) | 0.55 | 46 | 3.5 (0.8, 7.3) | 0.45 |  | 2.1 (0.0, 4.5) | 0.084 |
|  | High | 40 | 1.0 (0.0, 2.8) | 0.39 | 35 | 4.0 (2.0, 6.0) | 0.43 |  | 1.8 (0.0, 3.5) | 0.14 |
| ^a^ High (P4a), moderate (P6), and low (P1) caries phenotypes with Swedish ethnicity defined by *PRH1*, *PRH2* genetic variation. | | | | | | | | | | |
| ^b^ Caries DeFS (Decayed, enamel-included, Filled Surfaces) at 12 and 17 years of age. | | | | | | | | | | |
| ^c^ΔDeFS (5y) = 5-year prospective caries increment from 12 to 17 years of age. | | | | | | | | | | |
| ^d^ 2-sided P value from Mann–Whitney U test. | | | | | | | | | | |
| ^e^ *S. mutans*, Lactobacilli and Streptococci load in whole saliva measured by culture counts at 12 years of age. | | | | | | | | | | |
| ^f^ *S. mutans* infection and load (Negative < 10,000 CFU, Low 10,000 – 300,000 CFU, High > 300,000 CFU). | | | | | | | | | | |
| ^g^ Lactobacilli infection and load (Negative < 20,000 CFU, Low 20,000 – 100,000 CFU, High > 100,000 CFU). | | | | | | | | | | |
| ^h^ Streptococci infection and load (Low < 10,000,000 CFU, Moderate 10,000,000 – 50,000,000 CFU, High > 50,000,000 CFU). | | | | | | | | | | |

| **Table S3**. Influence of load of *S. mutans*, lactobacilli, and streptococci on caries progression in the high (P4a), moderate (P6), and low (P1) caries phenotypes defined by *PRH1, PRH2* genetic variation (adolescents with orthodontic brackets excluded) | | | | | | | | | | | | |
| --- | --- | --- | --- | --- | --- | --- | --- | --- | --- | --- | --- | --- |
| Bacteria | *PRH1/2^a^* | Load |  | DeFS-12y^b^ | |  |  | DeFS-17y^b^ | |  | ΔDeFS-5y^c^ |  |
|  |  |  | n | mean ± SD | P^d^ |  | n | mean ± SD | P^d^ |  | mean ± SD | P^d^ |
| *S. mutans*^e,f^ | P4a | Negative | 30 | 2.1 ± 2.1 | Ref. |  | 26 | 6.4 ± 8.7 | Ref. |  | 4.4 ± 8.2 | Ref. |
|  |  | Low | 18 | 2.8 ± 2.3 | 0.29 |  | 13 | 5.5 ± 3.3 | 0.35 |  | 2.6 ± 2.4 | 0.74 |
|  |  | High | 16 | 4.3 ± 4.2 | 0.056 |  | 15 | 11.4 ± 10.6 | 0.042 |  | 6.6 ± 6.9 | 0.20 |
|  |  |  |  |  |  |  |  |  |  |  |  |  |
|  | P6 | Negative | 47 | 1.4 ± 1.8 | Ref. |  | 43 | 4.1 ± 3.7 | Ref. |  | 2.5 ± 2.9 | Ref. |
|  |  | Low^f^ | 18 | 2.1 ± 2.1 | 0.27 |  | 16 | 6.1 ± 6.1 | 0.48 |  | 4.0 ± 4.4 | 0.34 |
|  |  | High^f^ | 23 | 4.4 ± 3.1 | <0.0001 |  | 20 | 11.0 ± 12.1 | 0.00018 |  | 6.5 ± 9.7 | 0.010 |
|  |  |  |  |  |  |  |  |  |  |  |  |  |
|  | P1 | Negative | 49 | 1.6 ± 2.1 | Ref. |  | 38 | 3.7 ± 4.4 | Ref. |  | 2.1 ± 3.0 | Ref. |
|  |  | Low^f^ | 23 | 2.0 ± 2.4 | 0.44 |  | 19 | 5.3 ± 6.1 | 0.41 |  | 3.9 ± 5.8 | 0.37 |
|  |  | High^f^ | 23 | 2.7 ± 2.2 | 0.021 |  | 21 | 8.2 ± 7.7 | 0.0019 |  | 5.3 ± 6.4 | 0.0057 |
|  |  |  |  |  |  |  |  |  |  |  |  |  |
| Lactobacilli^e,g^ | P4a | Negative | 32 | 2.6 ± 3.4 | Ref. |  | 29 | 5.5 ± 7.5 | Ref. |  | 2.9 ± 4.3 | Ref. |
|  |  | Low | 19 | 2.6 ± 2.0 | 0.38 |  | 14 | 7.1 ± 4.6 | 0.056 |  | 4.1 ± 3.7 | 0.15 |
|  |  | High | 13 | 3.9 ± 2.9 | 0.10 |  | 11 | 13.8 ± 12.3 | 0.015 |  | 9.6 ± 12.1 | 0.12 |
|  |  |  |  |  |  |  |  |  |  |  |  |  |
|  | P6 | Negative | 46 | 1.9 ± 2.7 | Ref. |  | 41 | 4.2 ± 4.3 | Ref. |  | 2.5 ± 3.1 | Ref. |
|  |  | Low | 24 | 2.3 ± 2.5 | 0.56 |  | 22 | 8.1 ± 12.1 | 0.12 |  | 5.6 ± 9.4 | 0.044 |
|  |  | High | 18 | 3.5 ± 2.0 | 0.0018 |  | 16 | 8.7 ± 5.3 | 0.0034 |  | 4.9 ± 4.2 | 0.025 |
|  |  |  |  |  |  |  |  |  |  |  |  |  |
|  | P1 | Negative | 54 | 1.7 ± 2.0 | Ref. |  | 43 | 4.8 ± 5.4 | Ref. |  | 3.1 ± 4.5 | Ref. |
|  |  | Low | 28 | 2.1 ± 2.8 | 0.63 |  | 24 | 5.3 ± 6.8 | 1.0 |  | 3.7 ± 5.8 | 0.96 |
|  |  | High | 13 | 2.8 ± 2.0 | 0.036 |  | 11 | 7.1 ± 7.2 | 0.20 |  | 4.2 ± 5.3 | 0.52 |
|  |  |  |  |  |  |  |  |  |  |  |  |  |
| Streptococci^e,h^ | P4a | Low | 20 | 2.4 ± 1.8 | Ref. |  | 17 | 5.2 ± 6.0 | Ref. |  | 2.4 ± 4.2 | Ref. |
|  |  | Moderate | 22 | 3.1 ± 3.5 | 0.89 |  | 20 | 7.8 ± 10.1 | 0.83 |  | 4.4 ± 5.9 | 0.30 |
|  |  | High | 22 | 3.0 ± 3.2 | 0.93 |  | 17 | 9.7 ± 8.8 | 0.026 |  | 6.9 ± 9.4 | 0.065 |
|  |  |  |  |  |  |  |  |  |  |  |  |  |
|  | P6 | Low | 24 | 1.8 ± 2.2 | Ref. |  | 22 | 3.5 ± 3.3 | Ref. |  | 2.0 ± 2.0 | Ref. |
|  |  | Moderate | 33 | 2.7 ± 3.0 | 0.19 |  | 29 | 8.8 ± 10.9 | 0.012 |  | 5.7 ± 8.6 | 0.042 |
|  |  | High | 31 | 2.4 ± 2.3 | 0.28 |  | 28 | 5.7 ± 4.8 | 0.14 |  | 3.3 ± 3.3 | 0.31 |
|  |  |  |  |  |  |  |  |  |  |  |  |  |
|  | P1 | Low | 14 | 2.2 ± 2.1 | Ref. |  | 11 | 4.1 ± 2.9 | Ref. |  | 2.1 ± 2.2 | Ref. |
|  |  | Moderate | 46 | 2.1 ± 2.3 | 0.75 |  | 37 | 5.6 ± 6.0 | 0.79 |  | 3.6 ± 5.0 | 0.56 |
|  |  | High | 35 | 1.6 ± 2.2 | 0.20 |  | 30 | 5.3 ± 7.0 | 0.73 |  | 3.7 ± 5.6 | 0.75 |
| ^a^ High (P4a), moderate (P6), and low (P1) caries phenotypes with Swedish ethnicity defined by *PRH1*, *PRH2* genetic variation. | | | | | | | | | | | | |
| ^b^ Caries DeFS (Decayed, enamel-included, Filled Surfaces) at 12 and 17 years of age. | | | | | | | | | | | | |
| ^c^ΔDeFS (5y) = 5-year prospective caries increment from 12 to 17 years of age. | | | | | | | | | | | | |
| ^d^ 2-sided P value from Mann–Whitney U test. | | | | | | | | | | | | |
| ^e^ *S. mutans*, Lactobacilli and Streptococci load in whole saliva measured by culture counts. | | | | | | | | | | | | |
| ^f^ *S. mutans* infection and load (Negative < 10,000 CFU, Low 10,000 – 300,000 CFU, High > 300,000 CFU). | | | | | | | | | | | | |
| ^g^ Lactobacilli infection and load (Negative < 20,000 CFU, Low 20,000 – 100,000 CFU, High > 100,000 CFU). | | | | | | | | | | | | |
| ^h^ Streptococci infection and load (Low < 10,000,000 CFU, Moderate 10,000,000 – 50,000,000 CFU, High > 50,000,000 CFU). | | | | | | | | | | | | |

| **Table S4**. Influence of load of *S. mutans*, lactobacilli, and streptococci on caries progression in the high (P4a), moderate (P6), and low (P1) caries phenotypes defined by *PRH1, PRH2* genetic variation (adolescents with orthodontic brackets excluded) | | | | | | | | | | | | |
| --- | --- | --- | --- | --- | --- | --- | --- | --- | --- | --- | --- | --- |
| Bacteria | *PRH1/2^a^* | Load |  | DeFS-12y^b^ | |  |  | DeFS-17y^b^ | |  | ΔDeFS-5y^c^ |  |
|  |  |  | n | Median (IQR) | P^d^ |  | n | Median (IQR) | P^d^ |  | Median (IQR) | P^d^ |
| *S. mutans*^e,f^ | P4a | Negative | 30 | 2.0 (0.0, 4.0) | Ref. |  | 26 | 3.5 (1.0, 8.3) | Ref. |  | 1.9 (0.0, 5.1) | Ref. |
|  |  | Low | 18 | 3.0 (0.8, 4.3) | 0.29 |  | 13 | 5.0 (3.0, 8.5) | 0.35 |  | 3.1 (0.6, 4.3) | 0.74 |
|  |  | High | 16 | 2.5 (1.0, 7.0) | 0.056 |  | 15 | 8.0 (5.0, 16.0) | 0.042 |  | 5.9 (0.0, 12.7) | 0.20 |
|  |  |  |  |  |  |  |  |  |  |  |  |  |
|  | P6 | Negative | 47 | 0.0 (0.0, 3.0) | Ref. |  | 43 | 3.0 (1.0, 6.0) | Ref. |  | 1.8 (0.0, 4.4) | Ref. |
|  |  | Low^f^ | 18 | 2.0 (0.0, 3.3) | 0.27 |  | 16 | 5.0 (0.0, 13.8) | 0.48 |  | 3.1 (0.0, 8.4) | 0.34 |
|  |  | High^f^ | 23 | 4.0 (2.0, 7.0) | <0.0001 |  | 20 | 10.0 (6.3, 10.0) | 0.00018 |  | 4.1 (1.0, 6.9) | 0.010 |
|  |  |  |  |  |  |  |  |  |  |  |  |  |
|  | P1 | Negative | 49 | 1.0 (0.0, 2.5) | Ref. |  | 38 | 3.0 (0.0, 5.0) | Ref. |  | 1.4 (0.0, 3.2) | Ref. |
|  |  | Low^f^ | 23 | 1.0 (0.0, 4.0) | 0.44 |  | 19 | 3.0 (2.0, 9.0) | 0.41 |  | 1.8 (0.0, 5.1) | 0.37 |
|  |  | High^f^ | 23 | 2.0 (1.0, 5.0) | 0.021 |  | 21 | 6.0 (3.5, 9.5) | 0.0019 |  | 3.4 (1.7, 5.8) | 0.0057 |
|  |  |  |  |  |  |  |  |  |  |  |  |  |
| Lactobacilli^e,g^ | P4a | Negative | 32 | 2.0 (0.0, 3.8) | Ref. |  | 29 | 3.0 (1.0, 8.0) | Ref. |  | 1.7 (0.0, 3.9) | Ref. |
|  |  | Low | 19 | 3.0 (0.0, 4.0) | 0.38 |  | 14 | 5.5 (3.8, 9.3) | 0.056 |  | 3.7 (1.2, 6.2) | 0.15 |
|  |  | High | 13 | 5.0 (1.0, 6.0) | 0.10 |  | 11 | 8.0 (4.0, 25.0) | 0.015 |  | 6.0 (0.0, 15.5) | 0.12 |
|  |  |  |  |  |  |  |  |  |  |  |  |  |
|  | P6 | Negative | 46 | 1.0 (0.0, 3.0) | Ref. |  | 41 | 3.0 (0.0, 7.5) | Ref. |  | 0.9 (0.0, 4.2) | Ref. |
|  |  | Low | 24 | 2.0 (0.0, 4.8) | 0.56 |  | 22 | 5.0 (1.0, 10.3) | 0.12 |  | 3.4 (1.1, 6.1) | 0.044 |
|  |  | High | 18 | 4.0 (2.5, 4.0) | 0.0018 |  | 16 | 7.0 (5.3, 12.3) | 0.0034 |  | 3.7 (1.1, 8.3) | 0.025 |
|  |  |  |  |  |  |  |  |  |  |  |  |  |
|  | P1 | Negative | 54 | 1.0 (0.0, 3.0) | Ref. |  | 43 | 3.0 (2.0, 6.0) | Ref. |  | 1.7 (0.7, 3.7) | Ref. |
|  |  | Low | 28 | 1.5 (0.0, 2.8) | 0.63 |  | 24 | 3.0 (0.3, 8.3) | 1.0 |  | 2.4 (0.0, 5.1) | 0.96 |
|  |  | High | 13 | 2.0 (1.0, 4.5) | 0.036 |  | 11 | 5.0 (3.0, 7.0) | 0.20 |  | 2.1(1.1, 4.5) | 0.52 |
|  |  |  |  |  |  |  |  |  |  |  |  |  |
| Streptococci^e,h^ | P4a | Low | 20 | 2.5 (0.3, 3.8) | Ref. |  | 17 | 4.0 (2.5, 5.5) | Ref. |  | 1.7 (0.0, 3.9) | Ref. |
|  |  | Moderate | 22 | 2.0 (0.0, 4.3) | 0.89 |  | 20 | 4.0 (2.0, 10.5) | 0.83 |  | 2.3 (0.0, 6.4) | 0.30 |
|  |  | High | 22 | 2.0 (0.0, 6.3) | 0.93 |  | 17 | 8.0 (5.0, 10.5) | 0.026 |  | 4.2 (1.4, 8.9) | 0.065 |
|  |  |  |  |  |  |  |  |  |  |  |  |  |
|  | P6 | Low | 24 | 0.0 (0.0, 3.8) | Ref. |  | 22 | 2.0 (0.8, 7.0) | Ref. |  | 1.4 (0.0, 3.4) | Ref. |
|  |  | Moderate | 33 | 2.0 (0.0, 4.0) | 0.19 |  | 29 | 7.0 (3.0, 10.5) | 0.012 |  | 4.0 (0.8, 7.4) | 0.042 |
|  |  | High | 31 | 2.0 (0.0, 4.0) | 0.28 |  | 28 | 6.0 (1.0, 10.0) | 0.14 |  | 2.6 (0.2, 6.5) | 0.31 |
|  |  |  |  |  |  |  |  |  |  |  |  |  |
|  | P1 | Low | 14 | 2.0 (0.0, 4.3) | Ref. |  | 11 | 3.0 (2.0, 7.0) | Ref. |  | 1.1 (0.9, 3.6) | Ref. |
|  |  | Moderate | 46 | 2.0 (0.0, 3.3) | 0.75 |  | 37 | 4.0 (1.5, 9.0) | 0.79 |  | 1.7 (0.4, 5.0) | 0.56 |
|  |  | High | 35 | 1.0 (0.0, 2.0) | 0.20 |  | 30 | 3.0 (1.8, 5.3) | 0.73 |  | 2.0 (0.0, 3.5) | 0.75 |
| ^a^ High (P4a), moderate (P6), and low (P1) caries phenotypes with Swedish ethnicity defined by *PRH1*, *PRH2* genetic variation. | | | | | | | | | | | | |
| ^b^ Caries DeFS (Decayed, enamel-included, Filled Surfaces) at 12 and 17 years of age. | | | | | | | | | | | | |
| ^c^ΔDeFS (5y) = 5-year prospective caries increment from 12 to 17 years of age. | | | | | | | | | | | | |
| ^d^ 2-sided P value from Mann–Whitney U test. | | | | | | | | | | | | |
| ^e^ *S. mutans*, Lactobacilli and Streptococci load in whole saliva measured by culture counts. | | | | | | | | | | | | |
| ^f^ *S. mutans* infection and load (Negative < 10,000 CFU, Low 10,000 – 300,000 CFU, High > 300,000 CFU). | | | | | | | | | | | | |
| ^g^ Lactobacilli infection and load (Negative < 20,000 CFU, Low 20,000 – 100,000 CFU, High > 100,000 CFU). | | | | | | | | | | | | |
| ^h^ Streptococci infection and load (Low < 10,000,000 CFU, Moderate 10,000,000 – 50,000,000 CFU, High > 50,000,000 CFU). | | | | | | | | | | | | |

| **Table S5**. Influence of load of *S. mutans*, lactobacilli and streptococci on caries progression in 452 adolescents | | | | | | | | | |
| --- | --- | --- | --- | --- | --- | --- | --- | --- | --- |
| Bacterial load |  | DeFS-12y^c^ | |  | DeFS-17y^c^ | |  | ΔDeFS-5y^d^ | |
| Percentile^a,b^ (CFU) | n | median (IQR) | P^e^ | n | median | P^e^ |  | median | P^e^ |
| *S. mutans*^a^ |  |  |  |  |  |  |  |  |  |
| Neg (0) | 185 | 1.0 (0.0, 2.5) | 0.0033 | 166 | 3.0 (1.0, 6.0) | 0.021 |  | 1.7 (0.0, 3.8) | 0.076 |
| < *P*20 (1–32960) | 53 | 2.0 (0.0, 4.5) | Ref. | 41 | 5.0 (3.0, 6.0) | Ref. |  | 3.3 (0.4, 5.0) | Ref. |
| *P*20-40 (32961–98100) | 53 | 2.0 (0.5, 4.0) | 0.87 | 44 | 5.0 (2.0, 12.0) | 0.78 |  | 2.6 (1.1, 7.4) | 0.85 |
| *P*40-60 (98101–508000) | 54 | 3.0 (0.0, 5.0) | 0.19 | 46 | 6.0 (2.0, 9.0) | 0.74 |  | 2.3 (0.6, 4.3) | 0.53 |
| *P*60-80 (508001–1380000) | 54 | 4.0 (2.0, 6.3) | 0.0021 | 46 | 9.5 (5.8, 15.3) | 0.00086 |  | 5.1 (1.7, 10.1) | 0.025 |
| >*P*80 (>1380000) | 53 | 3.0 (2.0, 6.0) | 0.021 | 47 | 7.0 (4.0, 13.0) | 0.026 |  | 4.5 (1.8, 9.3) | 0.090 |
|  |  |  |  |  |  |  |  |  |  |
| Lactobacilli^b^ |  |  |  |  |  |  |  |  |  |
| Neg (0) | 61 | 1.0 (0.0, 2.5) | 0.32 | 53 | 3.0 (0.0, 6.0) | 0.056 |  | 1.2 (0.0, 3.7) | 0.050 |
| < *P*20 (1-2480) | 78 | 2.0 (0.0, 3.0) | Ref. | 71 | 4.0 (2.0, 8.0) | Ref. |  | 2.5 (0.0, 5.8) | Ref. |
| *P*20-40 (2480-12720) | 78 | 1.5 (0.0, 4.0) | 0.64 | 65 | 3.0 (1.0, 8.0) | 0.42 |  | 1.8 (0.0, 4.8) | 0.38 |
| *P*40-60 (12721-32400) | 78 | 2.0 (0.0, 5.0) | 0.020 | 67 | 6.0 (2.0, 9.0) | 0.43 |  | 2.5 (0.9, 4.9) | 0.93 |
| *P*60-80 (32401-100702) | 79 | 2.0 (0.0, 4.0) | 0.026 | 67 | 5.0 (2.0, 12.0) | 0.31 |  | 3.3 (0.8, 7.0) | 0.42 |
| >*P*80 (>100702) | 78 | 3.0 (1.0, 5.0) | <0.0001 | 67 | 7.0 (4.0, 13.0) | 0.00025 |  | 3.4 (1.8, 8.9) | 0.015 |
|  |  |  |  |  |  |  |  |  |  |
| Streptococci^b^ |  |  |  |  |  |  |  |  |  |
| Neg (0) | 1 | 3.0 | - |  | 3.0 | - |  | 0.0 | - |
| < *P*20 (1-9140000) | 90 | 2.0 (0.0, 5.0) | Ref. | 79 | 3.0 (1.0, 7.0) | Ref. |  | 1.1 (0.0, 3.7) | Ref. |
| *P*20-40 (9140001-26000000) | 87 | 2.0 (0.0, 4.0) | 0.92 | 78 | 5.0 (2.0, 9.3) | 0.12 |  | 2.5 (0.0, 5.4) | 0.030 |
| *P*40-60 (26000001-46000000) | 91 | 2.0 (0.0, 4.0) | 0.95 | 76 | 5.0 (2.0, 9.0) | 0.058 |  | 3.3 (0.8, 6.1) | 0.015 |
| *P*60-80 (46000001-85000000) | 90 | 2.0 (0.0, 4.0) | 0.48 | 76 | 5.5 (3.0, 9.0) | 0.0096 |  | 2.5 (0.9, 6.6) | 0.012 |
| >*P*80 (>85000000) | 92 | 2.0 (0.0, 4.0) | 0.85 | 79 | 6.0 (2.0, 10.0) | 0.014 |  | 3.1 (1.0, 6.1) | 0.0047 |
| ^a^ Percentiles (*P*) of *S. mutans* quantity measured by qPCR (CFU) in whole saliva at 12 years of age. | | | | | | | | | |
| ^b^ Percentiles *(P)* of Lactobacilli and total Streptococci quantity measured by culture (CFU) of whole saliva at 12 years of age. | | | | | | | | | |
| ^c^ Caries DeFS (Decayed, enamel included, Filled Surfaces) at 12 and 17 years of age. | | | | | | | | | |
| ^d^ ΔDeFS (5y) = 5-years prospective caries increment from 12 to 17 years of age. | | | | | | | | | |
| ^e^ 2-sided P value from Mann-Whitney U-test. | | | | | | | | | |

| **Table S6**. Influence of residency and temporal changes of *S. mutans* adhesion phenotypes on caries experience and progression in 452 adolescents | | | | | | | | | | | | | | | |
| --- | --- | --- | --- | --- | --- | --- | --- | --- | --- | --- | --- | --- | --- | --- | --- |
| Adhesion type^a^ | Infection^b^ | |  | Frequency^c^ | | Stab^d^ | DeFS-12y^e^ | |  | DeFS-17y^e^ | |  | ΔDeFS-5y^f^ | | |
|  | 12y | 17y |  | % | n | (%) | mean ± SD | P*^g^* |  | mean ± SD | P*^g^* |  | mean ± SD | P*^g^* | |
| *S. mutans* | |  |  |  |  |  |  |  |  |  |  |  |  |  | |
|  | + | + |  | 40.7 | 158 | 85 | 3.5 ± 3.1 | <0.0001 |  | 9.8 ± 9.1 | <0.0001 |  | 6.0 ± 7.3 | <0.0001 | |
|  | + | - |  | 7.0 | 27 |  | 2.5 ± 2.5 | 0.052 |  | 5.1 ± 5.0 | 0.41 |  | 2.8 ± 5.0 | 0.56 | |
|  | - | + |  | 14.9 | 58 |  | 1.8 ± 1.9 | 0.31 |  | 5.7 ± 6.0 | 0.048 |  | 3.6 ± 5.3 | 0.073 | |
|  | - | - |  | 37.4 | 145 |  | 1.6 ± 2.0 | Ref. |  | 4.5 ± 5.6 | Ref. |  | 2.9 ± 4.7 | Ref. | |
|  | | |  |  |  |  |  |  |  |  |  |  |  |  | |
| Mixed SpaP ABC | | |  |  |  |  |  |  |  |  |  |  |  |  | |
|  | mix | mix |  | 2.6 | 10 | 37 | 4.7 ± 3.3 |  |  | 11.6 ± 7.1 |  |  | 6.6 ± 4.9 |  | |
|  | mix | dom |  | 2.6 | 10 | 37 | 4.2 ± 3.0 |  |  | 12.9 ± 11.4 |  |  | 7.9 ± 8.3 |  | |
|  | dom | mix |  | 3.6 | 14 |  | 4.5 ± 3.1 |  |  | 13.1 ± 11.1 |  |  | 8.4 ± 10.3 |  | |
|  | mix | - |  | 1.8 | 7 |  | 3.3 ± 3.3 |  |  | 8.0 ± 8.0 |  |  | 4.3 ± 4.8 |  | |
|  | - | mix |  | 2.3 | 9 |  | 3.0 ± 1.5 |  |  | 11.2 ± 10.4 |  |  | 8.1 ± 11.2 |  | |
|  |  |  |  |  |  |  |  |  |  |  |  |  |  |  | |
| SpaP A/B/C | |  |  |  |  |  |  |  |  |  |  |  |  |  | |
|  | A | A |  | 18.6 | 72 | 80 | 3.0 ± 3.0 |  |  | 8.7 ± 8.7 |  |  | 5.5 ± 7.4 |  | |
|  | A | mix |  | 1.5 | 6 | 7 | 4.3 ± 3.1 |  |  | 9.7 ± 5.1 |  |  | 5.2 ± 4.7 |  | |
|  | mix | A |  | 1.8 | 7 |  | 3.7 ± 2.4 |  |  | 12.6 ± 12.3 |  |  | 7.8 ± 8.8 |  | |
|  | A | - |  | 3.1 | 12 |  | 2.1 ± 2.4 |  |  | 5.1 ± 5.9 |  |  | 3.2 ± 6.8 |  | |
|  | - | A |  | 7,7 | 30 |  | 2.2 ± 2.2 |  |  | 6.0 ± 5.7 |  |  | 3.9 ± 5.3 |  | |
|  |  |  |  |  |  |  |  |  |  |  |  |  |  |  | |
|  | B | B |  | 6.4 | 25 | 51 | 3.9 ± 3.9 |  |  | 10.5 ± 12.2 |  |  | 5.8 ± 9.0 |  | |
|  | B | mix |  | 2.1 | 8 | 16 | 4.6 ± 3.2 |  |  | 15.6 ± 13.9 |  |  | 10.8 ± 12.9 |  | |
|  | B | A or C |  | 1.0 | 4 |  |  |  |  |  |  |  |  |  | |
|  | mix | B |  | 0.8 | 3 |  | 4.0 ± 4.6 |  |  | 12.3 ± 12.8 |  |  | 8.0 ± 8.9 |  | |
|  | B | - |  | 3.1 | 12 |  | 3.0 ± 2.3 |  |  | 7.8 ± 4.1 |  |  | 4.6 ± 3.1 |  | |
|  | - | B |  | 2.8 | 11 |  | 1.2 ± 2.1 |  |  | 4.5 ± 4.0 |  |  | 3.1 ± 2.9 |  | |
|  |  |  |  |  |  |  |  |  |  |  |  |  |  |  | |
|  | C | C |  | 3.1 | 12 | 67 | 3.0 ± 1.6 |  |  | 8.4 ± 5.3 |  |  | 5.4 ± 4.4 |  | |
|  | C | A |  |  | 1 |  |  |  |  |  |  |  |  |  | |
|  | C | - |  | 1.3 | 5 |  | 4.0 ± 2.4 |  |  | 6.0 ± 3.7 |  |  | 2.2 ± 2.4 |  | |
|  | - | C |  | 3.1 | 11 |  | 2.0 ± 2.0 |  |  | 4.9 ± 4.2 |  |  | 2.9 ± 3.0 |  | |
|  | | |  |  |  |  |  |  |  |  |  |  |  |  | |
| Mixed vs dom SpaP | | |  |  |  |  |  |  |  |  |  |  |  |  | |
|  | mix/dom^h^ | mix/dom^h^ |  | 8.8 | 34 |  | 4.5 ± 3.0 | 0.015 |  | 12.6 ± 9.9 | 0.019 |  | 7.7 ± 8.2 | 0.10 | |
|  | dom | dom |  | 29.0 | 114 |  | 3.2 ± 3.1 | Ref. |  | 9.1 ± 9.3 | Ref. |  | 5.5 ± 7.5 | Ref. | |
|  |  |  |  |  |  |  |  |  |  |  |  |  |  |  | |
|  | - | mix |  | 2.3 | 9 |  | 3.0 ± 1.5 | 0.13 |  | 11.2 ± 10.4 | 0.035 |  | 8.1 ± 11.2 | 0.080 | |
|  | - | dom |  | 14.7 | 52 |  | 2.0 ± 2.1 | Ref. |  | 5.5 ± 5.0 | Ref. |  | 3.5 ± 4.4 | Ref. | |
|  |  |  |  |  |  |  |  |  |  |  |  |  |  |  | |
| Cnm/Cbm | |  |  |  |  |  |  |  |  |  |  |  |  |  | |
|  | Cnm | Cnm |  | 4.4 | 17 | 85 | 3.9 ± 2.2 | 0.0032 |  | 11.2 ± 9.2 | 0.0067 |  | 6.9 ± 7.5 | 0.048 | |
|  | Cnm | - |  | 0.8 | 3 |  | 4.3 ± 4.0 | 0.33 |  | 5.7 ± 3.1 | 0.78 |  | 1.3 ± 1.3 | 0.37 | |
|  | - | Cnm |  | 0.5 | 2 |  | 3.5 ± 2.1 | 0.85 |  | 6.5 ± 5.0 | 0.71 |  | 2.3 ± 6.5 | 0.66 | |
|  | - | - |  | 94.3 | 366 |  | 2.4 ± 2.7 | Ref. |  | 6.7 ± 7.6 | Ref. |  | 4.2 ± 6.0 | Ref. | |
|  |  |  |  |  |  |  |  |  |  |  |  |  |  |  | |
|  | Cbm | Cbm |  | 1.3 | 5 | 100 | 1.0 ± 1.4 | 0.21 |  | 3.8 ± 2.8 | 0.44 |  | 2.4 ± 1.6 | 0.83 | |
|  | - | Cbm |  | 0.3 | 1 |  | 0 | - |  | 9.0 | - |  | 8.0 | - | |
|  | - | - |  | 98.4 | 382 |  | 2.5 ± 2.7 | Ref. |  | 6.9 ± 7.7 | Ref. |  | 4.3 ± 6.2 | Ref. | |
| ^a^ Infection with *S. mutans* adhesion types at 12 and 17 years of age measured by qPCR. | | | | | | | | | | | |  |  |  | |
| ^b^ +, adolescents infected with *S. mutans*; -, adolescents not infected with *S. mutans*; mix, infection with more than one SpaP A, B, or C type; dom, children infected with one dominant SpaP type. | | | | | | | | | | | | | | | |
| ^c^ In total, 388 adolescents could provide saliva samples for qPCR analysis at both 12 and 17 years of age. | | | | | | | | | | | | | | | |
| ^d^ Stability was defined as proportion of the adolescents infected with *S. mutans* adhesion type at 12 and 17 years of age. | | | | | | | | | | | | | | | |
| ^e^ Caries DeFS (Decayed, enamel-included, Filled Surfaces) at 12 and 17 years of age. | | | | | | | | | | | |  |  | |  |
| ^f^ ΔDeFS (5y) = 5-year prospective caries increment from 12 to 17 years of age. | | | | | | | | | | |  |  |  | |  |
| ^g^ 2-sided P value from Mann–Whitney U test. | | | | | | |  |  |  |  |  |  |  | |  |
| ^h^ mix/dom, adolescents infected with mixed SpaP regardless of a stable infection status (mix,mix) or switching between dominant and mixed SpaP (mix,dom or dom,mix). | | | | | | | | | | | | | | | |

| **Table S7**. Influence of residency and temporal changes of *S. mutans* adhesion phenotypes on caries experience and progression in 452 adolescents | | | | | | | | | | | | | | |
| --- | --- | --- | --- | --- | --- | --- | --- | --- | --- | --- | --- | --- | --- | --- |
| Adhesion type^a^ | Infection^b^ | |  | Frequency^c^ | | Stab^d^ | DeFS-12y^e^ | |  | DeFS-17y^e^ | |  | ΔDeFS-5y^f^ | |
|  | 12y | 17y |  | % | n | (%) | median (IQR) | P*^g^* |  | Median (IQR) | P*^g^* |  | Median (IQR) | P*^g^* |
| *S. mutans* | |  |  |  |  |  |  |  |  |  |  |  |  |  |
|  | + | + |  | 40.7 | 158 | 85 | 3.0 (1.0, 5.3) | <0.0001 |  | 8.0 (4.0, 12.3) | <0.0001 |  | 3.4 (1.3, 8.7) | <0.0001 |
|  | + | - |  | 7.0 | 27 |  | 2.0 (0.0, 5.0) | 0.052 |  | 4.0 (1.0, 9.0) | 0.41 |  | 1.1 (0.0, 4.1) | 0.56 |
|  | - | + |  | 14.9 | 58 |  | 1.0 (0.0, 3.0) | 0.31 |  | 5.0 (1.8, 7.0) | 0.048 |  | 2.8 (0.8, 4.4) | 0.073 |
|  | - | - |  | 37.4 | 145 |  | 1.0 (0.0, 2.0) | Ref. |  | 3.0 (1.0, 6.0) | Ref. |  | 1.7 (0.0, 3.9) | Ref. |
|  | | |  |  |  |  |  |  |  |  |  |  |  |  |
| Mixed SpaP ABC | | |  |  |  |  |  |  |  |  |  |  |  |  |
|  | mix | mix |  | 2.6 | 10 | 37 | 3.5 (2.8, 8.0) |  |  | 11.5 (4.8, 18.5) |  |  | 7.4 (2.0, 11.1) |  |
|  | mix | dom |  | 2.6 | 10 | 37 | 4.0 (1.8, 7.3) |  |  | 8.0 (5.5, 25.5) |  |  | 4.2 (1.6, 17.6) |  |
|  | dom | mix |  | 3.6 | 14 |  | 5.5 (1.5, 7.0) |  |  | 10.0 (16.3, 6.8) |  |  | 6.7 (1.5, 10.3) |  |
|  | mix | - |  | 1.8 | 7 |  | 3.0 (0.0, 5.0) |  |  | 5.0 (5.0, 9.0) |  |  | 3.7 (0.0, 4.5) |  |
|  | - | mix |  | 2.3 | 9 |  | 3.0 (2.0, 4.0) |  |  | 10.0 (4.0, 12.5) |  |  | 4.0 (3.1, 7.9) |  |
|  |  |  |  |  |  |  |  |  |  |  |  |  |  |  |
| SpaP A/B/C | |  |  |  |  |  |  |  |  |  |  |  |  |  |
|  | A | A |  | 18.6 | 72 | 80 | 2.0 (0.0, 5.0) |  |  | 6.5 (2.0, 12.5) |  |  | 3.0 (1.0, 6.7) |  |
|  | A | mix |  | 1.5 | 6 | 7 | 4.5 (1.6, 6.8) |  |  | 10.0 (5.0, 14.5) |  |  | 3.7 (1.5, 9.6) |  |
|  | mix | A |  | 1.8 | 7 |  | 4.0 (2.0, 5.0) |  |  | 8.0 (4.0, 25.0) |  |  | 3.5 (1.7, 17.5) |  |
|  | A | - |  | 3.1 | 12 |  | 1.5 (0.0, 3.0) |  |  | 3.5 (0.0, 7.8) |  |  | 0.0 (0.0, 4.8) |  |
|  | - | A |  | 7,7 | 30 |  | 2.0 (0.0, 4.0) |  |  | 4.5 (2.0, 8.3) |  |  | 2.4 (0.9, 4.5) |  |
|  |  |  |  |  |  |  |  |  |  |  |  |  |  |  |
|  | B | B |  | 6.4 | 25 | 51 | 3.0 (1.0, 5.5) |  |  | 7.0 (3.5, 12.0) |  |  | 3.3 (1.7, 7.2) |  |
|  | B | mix |  | 2.1 | 8 | 16 | 6.0 (0.8, 7.0) |  |  | 9.5 (7.5, 28.3) |  |  | 7.9 (1.1, 17.1) |  |
|  | B | A or C |  | 1.0 | 4 |  |  |  |  |  |  |  |  |  |
|  | mix | B |  | 0.8 | 3 |  | 7.0 (-) |  |  | 8.0 (-) |  |  | 4.9 (-) |  |
|  | B | - |  | 3.1 | 12 |  | 2.5 (1.3, 4.8) |  |  | 7.5 (5.0, 10.0) |  |  | 3.9 (2.7, 7.0) |  |
|  | - | B |  | 2.8 | 11 |  | 0.0 (0.0, 2.0) |  |  | 4.0 (0.0, 8.0) |  |  | 2.5 (0.0, 6.1) |  |
|  |  |  |  |  |  |  |  |  |  |  |  |  |  |  |
|  | C | C |  | 3.1 | 12 | 67 | 3.0 (2.0, 4.0) |  |  | 8.5 (3.5, 13.5) |  |  | 5.9 (1.3, 9.6) |  |
|  | C | A |  |  | 1 |  |  |  |  |  |  |  |  |  |
|  | C | - |  | 1.3 | 5 |  | 4.0 (2.0, 6.0) |  |  | 7.0 (2.5, 9.0) |  |  | 0.9 (0.4, 4.6) |  |
|  | - | C |  | 3.1 | 11 |  | 2.0 (0.0, 4.0) |  |  | 5.0 (0.0, 9.0) |  |  | 3.3 (0.0, 4.4) |  |
|  | | |  |  |  |  |  |  |  |  |  |  |  |  |
| Mixed vs dom SpaP | | |  |  |  |  |  |  |  |  |  |  |  |  |
|  | mix/dom^h^ | mix/dom^h^ |  | 8.8 | 34 |  | 4.0 (2.0, 7.0) | 0.015 |  | 9.5 (6.0, 17.3) | 0.019 |  | 5.4 (1.7, 11.1) | 0.10 |
|  | dom | dom |  | 29.0 | 114 |  | 2.0 (0.8, 5.0) | Ref. |  | 7.0 (3.0, 12.0) | Ref. |  | 3.2 (1.1, 7.3) | Ref. |
|  |  |  |  |  |  |  |  |  |  |  |  |  |  |  |
|  | - | mix |  | 2.3 | 9 |  | 3.0 (2.0, 4.0) | 0.13 |  | 10.0 (4.0, 12.5) | 0.035 |  | 4.0 (3.1, 7.9) | 0.080 |
|  | - | dom |  | 14.7 | 52 |  | 1.0 (0.0, 3.8) | Ref. |  | 4.5 (2.0, 8.0) | Ref. |  | 2.5 (0.8, 4.4) | Ref. |
|  |  |  |  |  |  |  |  |  |  |  |  |  |  |  |
| Cnm/Cbm | |  |  |  |  |  |  |  |  |  |  |  |  |  |
|  | Cnm | Cnm |  | 4.4 | 17 | 85 | 3.0 (2.5, 5.5) | 0.0032 |  | 9.0 (6.0, 14.0) | 0.0067 |  | 4.5 (2.0, 9.1) | 0.048 |
|  | Cnm | - |  | 0.8 | 3 |  | 5.0 (-) | 0.33 |  | 5.0 (-) | 0.78 |  | 1.1 (-) | 0.37 |
|  | - | Cnm |  | 0.5 | 2 |  | 3.5 (-) | 0.85 |  | 6.5 (-) | 0.71 |  | 2.3 (-) | 0.66 |
|  | - | - |  | 94.3 | 366 |  | 2.0 (0.0, 4.0) | Ref. |  | 5.0 (2.0, 9.0) | Ref. |  | 2.4 (0.7, 5.3) | Ref. |
|  |  |  |  |  |  |  |  |  |  |  |  |  |  |  |
|  | Cbm | Cbm |  | 1.3 | 5 | 100 | 0.0 (0.0, 2.5) | 0.21 |  | 5.0 (1.0, 6.0) | 0.44 |  | 2.4 (0.9, 3.9) | 0.83 |
|  | - | Cbm |  | 0.3 | 1 |  | 0.0 (-) | - |  | 9.0 (-) | - |  | 8.0 (-) | - |
|  | - | - |  | 98.4 | 382 |  | 2.0 (0.0, 4.0) | Ref. |  | 5.0 (2.0, 9.0) | Ref. |  | 2.5 (0.7, 5.6) | Ref. |
| ^a^ Infection with *S. mutans* adhesion types at 12 and 17 years of age measured by qPCR. | | | | | | | | | | | |  |  |  |
| ^b^ +, adolescents infected with *S. mutans*; -, adolescents not infected with *S. mutans*; mix, infection with more than one SpaP A, B, or C type; dom, children infected with one dominant SpaP type. | | | | | | | | | | | | | | |
| ^c^ In total, 388 adolescents could provide saliva samples for qPCR analysis at both 12 and 17 years of age. | | | | | | | | | | | | | | |
| ^d^ Stability was defined as proportion of the adolescents infected with *S. mutans* adhesion type at 12 and 17 years of age. | | | | | | | | | | | | | | |
| ^e^ Caries DeFS (Decayed, enamel-included, Filled Surfaces) at 12 and 17 years of age. | | | | | | | | | | | |  |  | |
| ^f^ ΔDeFS (5y) = 5-year prospective caries increment from 12 to 17 years of age. | | | | | | | | | | |  |  |  | |
| ^g^ 2-sided P value from Mann–Whitney U test. | | | | | | |  |  |  |  |  |  |  | |
| ^h^ mix/dom, adolescents infected with mixed SpaP regardless of a stable infection status (mix,mix) or switching between dominant and mixed SpaP (mix,dom or dom,mix). | | | | | | | | | | | | | | |

|  |  |  |  |  |  |  |  |  |  |  |  |  |  |  |  |  |  |  |  |  |
| --- | --- | --- | --- | --- | --- | --- | --- | --- | --- | --- | --- | --- | --- | --- | --- | --- | --- | --- | --- | --- |
| **Table S8**. Geographic distribution of *S. mutans* adhesion types in Swedish adolescents at 12 years of age in Västerbotten County, Sweden | | | | | | | | | | | | | | | | | | | |  |
| Adhesion type^a^ |  |  | Geographic division^b^ | | | | | | | | | | | | | | | | |  |
|  |  | Total | South | North | p^c^ |  | South | | | | | |  | | North | | | | | |
|  |  |  |  |  |  |  | urban | | rural | | P^c^ | |  | | urban | | rural | | P^c^ | |
| *S. mutans* | + | 217 | 178 | 39 | 0.53 |  | 122 | | 45 | | 0.30 | |  | | 32 | | 7 | | 1.0 | |
|  | - | 234 | 198 | 36 |  |  | 146 | | 52 | |  | |  | | 29 | | 7 | |  | |
|  |  |  |  |  |  |  |  | |  | |  | |  | |  | |  | |  | |
| SpaP A | + | 111 | 93 | 18 | 1.0 |  | 66 | | 27 | | 1.0 | |  | | 14 | | 4 | | 0.73 | |
|  | - | 340 | 283 | 57 |  |  | 202 | | 81 | |  | |  | | 47 | | 10 | |  | |
|  |  |  |  |  |  |  |  | |  | |  | |  | |  | |  | |  | |
| SpaP B | + | 71 | 59 | 12 | 1.0 |  | 36 | | 23 | | 0.06 | |  | | 10 | | 2 | | 1.0 | |
|  | - | 380 | 317 | 63 |  |  | 232 | | 85 | |  | |  | | 51 | | 12 | |  | |
|  |  |  |  |  |  |  |  | |  | |  | |  | |  | |  | |  | |
| B-1 | + | 28 | 23 | 5 | 0.80 |  | 15 | | 8 | | 0.48 | |  | | 4 | | 1 | | 1.0 | |
|  | - | 423 | 353 | 70 |  |  | 253 | | 100 | |  | |  | | 57 | | 13 | |  | |
|  |  |  |  |  |  |  |  | |  | |  | |  | |  | |  | |  | |
| SpaP C | + | 19 | 15 | 4 | 0.54 |  | 11 | | 4 | | 1.0 | |  | | 3 | | 1 | | 0.57 | |
|  | - | 432 | 361 | 71 |  |  | 257 | | 104 | |  | |  | | 58 | | 13 | |  | |
|  |  |  |  |  |  |  |  | |  | |  | |  | |  | |  | |  | |
| Cnm | + | 26 | 16 | 10 | 0.0050 |  | 11 | | 5 | | 0.78 | |  | | 7 | | 3 | | 0.38 | |
|  | - | 425 | 360 | 65 |  |  | 257 | | 103 | |  | |  | | 54 | | 11 | |  | |
|  |  |  |  |  |  |  |  | |  | |  | |  | |  | |  | |  | |
| Cbm | + | 7 | 7 | 0 | 0.61 |  | 4 | | 3 | | 0.42 | |  | | 0 | | 0 | | - | |
|  | - | 444 | 369 | 75 |  |  | 264 | | 105 | |  | |  | | 61 | | 14 | |  | |
|  |  |  |  |  |  |  |  | |  | |  | |  | |  | |  | |  | |
| Total |  | 451 | 376 | 75 |  |  | 268 | | 108 | |  | |  | | 61 | | 14 | |  | |
| ^a^ *S. mutans* genotypes measured by qPCR in whole saliva from 451 adolescents aged 12 years; one adolescent out of 452 could not give saliva at 12 years. | | | | | | | | | | | | | | | | | | | |  |
| ^b^ Geographic distribution of *S. mutans* genotypes was divided into South (Umeå) and North (Skellefteå), and further into urban and rural, depending on the origin of the host child. | | | | | | | | | | | | | | | | | | | |  |
| ^c^P value from Fisher’s exact test (2-sided). | | | | | | | |  | |  | |  | |  | |  | |  | |  |

| **Table S9**. Influence of residency and load of *S. mutans* dominant and mixed adhesion types on caries progression in 452 adolescents | | | | | | | | | | | | | | | | | |  |
| --- | --- | --- | --- | --- | --- | --- | --- | --- | --- | --- | --- | --- | --- | --- | --- | --- | --- | --- |
| *S. mutans* | Infection^a^ | |  | Freq & stab.^b^ | | |  | DeFS-12y^c^ | | |  | DeFS-17y^c^ | | |  | ΔDeFS-5y^d^ | | |
|  | 12y | 17y |  | % | n | (%) |  | median (IQR) | P^e^ | mean diff  (95% CI)^j^ |  | median  (IQR) | P^e^ | mean diff |  | median  (IQR) | P^e^ | mean diff |
|  |  |  |  |  |  |  |  |  |  |  |  |  |  | (95% CI)^j^ |  |  |  | (95% CI)^j^ |
| *S. m* | + | + |  | 40.7 | 158 | 85 |  | 3.0 (1.0, 5.3) | <0.0001 | 2.0 (1.4, 2.6) |  | 8.0 (4.0, 12.3) | <0.0001 | 5.3 (3.7, 6.9) |  | 3.4 (1.3, 8.7) | <0.0001 | 3.1 (1.8, 4.4) |
|  | + | - |  | 7.0 | 27 |  |  | 2.0 (0.0, 5.0) | 0.052 | 1.0 (0.0, 1.9) |  | 4.0 (1.0, 9.0) | 0.41 | 0.6 (-1.3, 2.7) |  | 1.1 (0.0, 4.1) | 0.56 | -0.1 (-2.0, 2.1) |
|  | - | + |  | 14.9 | 58 |  |  | 1.0 (0.0, 3.0) | 0.31 | 0.2 (-0.4, 0.8) |  | 5.0 (1.8, 7.0) | 0.048 | 1.1 (-0.5, 2.9) |  | 2.8 (0.8, 4.4) | 0.073 | 0.7 (-0.7, 2.3) |
|  | - | - |  | 37.4 | 145 |  |  | 1.0 (0.0, 2.0) | Ref. | Ref. |  | 3.0 (1.0, 6.0) | Ref. | Ref. |  | 1.7 (0.0, 3.9) | Ref. | Ref. |
|  |  |  |  |  |  |  |  |  |  |  |  |  |  |  |  |  |  |  |
| Refs^f^ | + |  |  |  | 217 |  |  | 3.0 (1.0, 5.0) | < 0.0001 | 1.9 (1.4, 2.3) |  | 7.0 (3.0, 12.0) | < 0.0001 | 4.3 (2.8, 5.9) |  | 3.4 (1.1, 7.9) | < 0.0001 | 2.4 (1.2, 3.6) |
|  | - |  |  |  | 234 |  |  | 1.0 (0.0, 3.0) | Ref. | Ref. |  | 3.0 (1.0, 6.0) | Ref. | Ref. |  | 2.1 (0.0, 4.1) | Ref. | Ref. |
|  |  |  |  |  |  |  |  |  |  |  |  |  |  |  |  |  |  |  |
| Mixed SpaP ABC^f^ | B | mix |  | 2.1 | 8 |  |  | 6.0 (0.8, 7.0) | 0.12 | 1.6 (-0.9, 3.8) |  | 9.5 (7.5, 28.3) | 0.072 | 7.3 (-1.8, 17.9) |  | 7.9 (1.1, 17.1) | 0.21 | 5.5 (-1.9, 16.0) |
|  | - | mix |  | 2.3 | 9 |  |  | 3.0 (2.0, 4.0) | 0.92 | -0.5 (-1.8, 0.6) |  | 10.0 (4.0, 12.5) | 0.53 | 2.9 (-4.5, 13.1) |  | 4.0 (3.1, 7.9) | 0.38 | 3.5 (-3.4, 14.9) |
|  | mix | B |  | 0.8 | 3 |  |  | 7.0 (-) | 0.29 | 2.3 (-3.1, 6.1) |  | 8.0 (-) | 0.30 | 5.3 (-3.1, 19.1) |  | 4.9 (-) | 0.46 | 2.8 ( -4.2, 13.3) |
|  | mix | A |  | 1.8 | 7 |  |  | 4.0 (2.0, 5.0) | 0.40 | 0.7 (-1.1, 2.7) |  | 8.0 (4.0, 25.0) | 0.37 | 4.2 (-4.5, 14.8) |  | 3.5 (1.7, 17.5) | 0.39 | 2.6 (-3.8, 10.0) |
|  | Cnm | Cnm |  | 4.4 | 17 | 85 |  | 3.0 (2.5, 5.5) | 0.11 | 0.8 (-0.3, 2.1) |  | 9.0 (6.0, 14.0) | 0.12 | 2.8 (-1.6, 8.0) |  | 4.5 (2.0, 9.1) | 0.21 | 1.7 (-1.8, 6.1) |
|  | mix | mix |  | 2.6 | 10 | 37 |  | 3.5 (2.8, 8.0) | 0.10 | 1.7 (-0.4, 3.8) |  | 11.5 (4.8, 18.5) | 0.10 | 3.2 (-1.3, 7.9) |  | 7.4 (2.0, 11.1) | 0.17 | 1.4 (-1.8, 4.6) |
|  | A | A |  | 18.6 | 72 | 80 |  | 2.0 (0.0, 5.0) | 0.76 | 0.1 (-0.9, 0.8) |  | 6.5 (2.0, 12.5) | 0.87 | 0.4 (-2.3, 2.9) |  | 3.0 (1.0, 6.7) | 0.81 | 0.3 ( -1.9, 2.6) |
|  | B | B |  | 6.4 | 25 | 51 |  | 3.0 (1.0, 5.5) | 0.39 | 0.9 (-0.6, 2.6) |  | 7.0 (3.5, 12.0) | 0.33 | 2.1 (-2.2, 7.8) |  | 3.3 (1.7, 7.2) | 0.56 | 0.6 (-2.7, 4.5) |
|  |  |  |  |  |  |  |  |  |  |  |  |  |  |  |  |  |  |  |
|  | mix/dom^g^ | mix/dom^g^ |  | 8.8 | 34 |  |  | 4.0 (2.0, 7.0) | 0.015 | 1.3 (0.1, 2.5) |  | 9.5 (6.0, 17.3) | 0.019 | 3.5 (0.0, 7.3) |  | 5.4 (1.7, 11.1) | 0.10 | 2.2 (-0.5, 5.4) |
|  | dom^g^ | dom^g^ |  | 29.0 | 114 |  |  | 2.0 (0.8, 5.0) | Ref. | Ref. |  | 7.0 (3.0, 12.0) | Ref. | Ref. |  | 3.2 (1.1, 7.3) | Ref. | Ref. |
|  |  |  |  |  |  |  |  |  |  |  |  |  |  |  |  |  |  |  |
| SpaP B-1 | |  |  | 3.3 | 15 |  |  | 7.0 (3.0, 8.0) | 0.0015 | 2.8 (1.0, 4.8) |  | 12.0 (7.0, 20.5) | 0.028 | 6.8 (0.3, 15.4) |  | 7.9 (2.4, 14.2) | 0.074 | 5.2 (-1.4, 14.3) |
| SpaP B-1 excl^h^ | |  |  | 12.3 | 56 |  |  | 3.0 (1.0, 5.8) | 0.29 | 0.7 (-0.2, 1.7) |  | 8.0 (3.8, 11.3) | 0.16 | 2.2 (-1.0, 5.9) |  | 3.4 (1.7, 7.9) | 0.26 | 0.8 (-1.8, 3.6) |
|  |  |  |  |  |  |  |  |  |  |  |  |  |  |  |  |  |  |  |
| Load *P*_60-80_^i^ | B |  |  |  | 17 |  |  | 5.0 (2.0, 7.5) | 0.036 | 2.2 (0.2, 4.4) |  | 10.0 (6.5, 32.0) | 0.012 | 9.9 (0.7, 20.2) |  | 6.9 (2.3, 17.3) | 0.031 | 6.9 (-0.5, 15.5) |
|  | Cnm |  |  |  | 6 |  |  | 5.0 (2.8, 7.3) | 0.059 | 2.0 (0.1, 4.1) |  | 10.0 (5.5, 32.5) | 0.11 | 8.8 (-2.8, 23.5) |  | 3.2 (1.4, 24.8) | 0.36 | 5.9 (-4.4, 19.0) |
|  | B-1 |  |  |  | 11 |  |  | 5.0 (4.0, 8.0) | 0.0011 | 3.2 (1.6, 5.3) |  | 9.0 (5.0, 27.0) | 0.088 | 7.2 (-1.7, 18.7) |  | 4.1 (0.9, 18.1) | 0.52 | 4.9 (-3.9, 16.4) |
|  | mix |  |  |  | 16 |  |  | 5.0 (4.0, 7.8) | 0.0061 | 2.2 (0.5, 3.8) |  | 9.5 (7.0, 19.8) | 0.0073 | 6.1 (0.7, 11.9) |  | 5.2 (3.4, 12.3) | 0.041 | 4.1 (-0.6, 9.7) |
|  | A |  |  |  | 23 |  |  | 4.0 (0.0, 6.0) | 0.39 | 0.7 (-0.9, 2.2) |  | 10.0 (4.3, 14.8) | 0.17 | 2.6 (-1.9, 7.5) |  | 5.3 (0.5, 8.7) | 0.29 | 1.7 (-2.1, 6.0) |
|  |  |  |  |  |  |  |  |  |  |  |  |  |  |  |  |  |  |  |
| Load >*P*_90_^i^ | + | + |  |  | 3 |  |  | 8.0 (-) | 0.25 | 2.6 (-3.2, 6.2) |  | 27.0 (-) | 0.038 | 17.0 (-1.1, 33.2) |  | 18.1 (-) | 0.024 | 15.6 (0.1, 33.8) |
|  | + | - |  |  | 21 |  |  | 3.0 (2.0, 5.0) | 0.56 | 0.3 (-0.9, 1.7) |  | 7.0 (3.0, 10.0) | 0.80 | -0.4 (-3.7, 3.2) |  | 3.3 (1.7, 7.6) | 0.52 | -0.1 (-2.8, 2.4) |
|  | + |  |  |  | 24 |  |  | 3.0 (2.0, 5.0) | 0.36 | 0.6 (-0.7, 2.0) |  | 7.0 (3.8, 11.5) | 0.35 | 2.0 (-2.3, 7.1) |  | 4.5 (2.0, 8.8) | 0.18 | 2.0 (-1.6, 6.5) |
|  |  |  |  |  |  |  |  |  |  |  |  |  |  |  |  |  |  |  |
| Refs | A |  |  | 24.6 | 111 |  |  | 2.0 (2.0, 7.0) | Ref. | Ref. |  | 6.0 (2.0, 10.8) | Ref. | Ref. |  | 2.7 (1.0, 6.7) | Ref. | Ref. |
|  | B |  |  | 15.7 | 71 |  |  | 4.0 (1.0, 7.0) | 0.034 | 1.1 (0.2, 2.1) |  | 8.0 (5.0, 14.0) | 0.045 | 3.0 (-0.1, 6.8) |  | 3.6 (1.7, 9.5) | 0.11 | 1.5 (-1.0, 4.6) |
|  | C |  |  | 4.2 | 19 |  |  | 3.0 (2.0, 5.0) | 0.37 | 0.3 (-0.8, 1.3) |  | 7.0 (3.8, 12.0) | 0.75 | -0.9 (-3.7, 1.8) |  | 2.7 (0.9, 7.6) | 0.93 | -0.9 (-3.4, 1.4) |
|  | Cnm |  |  | 5.8 | 26 |  |  | 4.0 (3.0, 6.3) | 0.012 | 1.4 (0.4, 2.5) |  | 8.5 (5.3, 13.5) | 0.18 | 2.0 (-1.8, 6.4) |  | 3.3 (1.1, 8.6) | 0.46 | 0.8 (-2.4, 4.6) |
|  | Cbm |  |  | 1.5 | 7 |  |  | 2.0 (0.0, 5.0) | 0.63 | -0.2 (-2.4, 2.0) |  | 5.0 (1.0, 6.0) | 0.20 | -4.6 (-7.6, 1.9) |  | 2.4 (0.9, 3.9) | 0.60 | -2.8 (-4.9, -1.0) |
| ^a^ Infection status at 12 and 17 years measured by qPCR or culturing (*P*_60-80_). | | | | | | | | | | | | | | | | | | |
| ^b^ Totally 388 adolescents were sampled for saliva at both 12 and 17 years of age. | | | | | | | | | | | | | | | | | | |
| ^c^ Caries DeFS (Decayed, enamel included, Filled Surfaces) at 12 and 17 years of age. | | | | | | | | | | | | | | | | | | |
| ^d^ ΔDeFS (5y) = 5-years prospective caries increment from 12 to 17 years of age. | | | | | | | | | | | | | | | | | | |
| ^e^ 2-sided P value from Mann-Whitney U-test. | | | | | | | | | | | | | | | | | | |
| ^f^ Mixed (mix) marks adolescents infected with mixed SpaP ABC type, dom marks adolescents infected with dominant SpaP A/B/C type. | | | | | | | | | | | | | | | | | | |
| ^g^ mix/dom, adolescents infected with mixed SpaP regardless of a stable infection status (mix,mix) or switching between dominant and mixed SpaP (mix,dom or dom,mix) | | | | | | | | | | | | | | | | | | |
| ^h^ SpaP B-1 excl marks adolescents infected with non-B-1 SpaP B type. | | | | | | | | | | | | | | | | | | |
| ^i^ *P* marks Percentile from culturing (*P*_60-80_) or qPCR (>*P*_90_). | | | | | | | | | | | | | | | | | | |
| ^j^ Mean difference estimate with 95% CIs by bootstrapping 1000 times | | | | | | | | | | | | | | | | | | |

| **Table S10**. Influence of *S. mutans* adhesin type and *S. mutans* load on caries experience and development in 452 adolescents from 12 to 17 years of age | | | | | | | |  |
| --- | --- | --- | --- | --- | --- | --- | --- | --- |
| *S.mutans* load*^a^* (CFU) | |  | DeFS-12y^b^ |  | DeFS-17y |  | ΔDeFS-5y^c^ |  |
|  |  | n | mean±SD |  | mean±SD |  | mean±SD |  |
| SpaP A | | | |  |  |  |  |  |
| <*P*_20_ |  | 7 | 2.0 ± 2.1 |  | 7.3 ± 5.6 |  | 4.9 ± 3.7 |  |
| *P*_20-40_ |  | 21 | 2.3 ± 1.9 |  | 6.2 ± 5.7 |  | 3.7 ± 4.6 |  |
| *P*_40-60_ |  | 25 | 2.6 ± 2.8 |  | 5.6 ± 5.5 |  | 3.2 ± 5.2 |  |
| *P*_60-80_ |  | 23 | 3.7 ± 3.2 |  | 11.0 ± 9.3 |  | 6.9 ± 8.3 |  |
| *>P*_80_ |  | 28 | 3.9 ± 3.1 |  | 9.8 ± 8.8 |  | 5.7 ± 6.4 |  |
| SpaP B | | | |  |  |  |  |  |
| <*P*_20_ |  | 8 | 4.1 ± 3.9 |  | 10.5 ± 7.2 |  | 6.0 ± 6.1 |  |
| *P*_20-40_ |  | 13 | 3.5 ± 3.7 |  | 7.9 ± 5.7 |  | 4.0 ± 3.7 |  |
| *P*_40-60_ |  | 16 | 3.8 ± 2.5 |  | 9.0 ± 7.6 |  | 4.9 ± 5.9 |  |
| *P*_60-80_ |  | 17 | 5.2 ± 4.3 |  | 18.2 ± 17.1 |  | 12.2 ± 14.3 |  |
| *>P*_80_ |  | 15 | 4.3 ± 3.3 |  | 10.2 ± 9.5 |  | 5.5 ± 6.6 |  |
| B-1 |  |  |  |  |  |  |  |  |
| <*P*_20_ |  | 6 | 3.5 ± 4.0 |  | 6.8 ± 3.6 |  | 5.0 ± 3.1 |  |
| *P*_20-40_ |  | 5 | 2.2 ± 2.7 |  | 7.0 ± 4.7 |  | 5.7 ± 3.4 |  |
| *P*_40-60_ |  | 4 | 4.5 ± 2.4 |  | 8.3 ± 3.5 |  | 4.0 ± 3.1 |  |
| *P*_60-80_ |  | 11 | 6.3 ± 2.9 |  | 15.6 ± 13.6 |  | 10.2 ± 14.0 |  |
| *>P*_80_ |  | 2 | 2.0 ± 1.4 |  | 9.5 ± 6.4 |  | 6.5 ± 4.3 |  |
| mixed SpaP A/B/C | | | |  |  |  |  |  |
| <*P*_20_ |  | 4 | 3.8 ± 2.9 |  | 12.3 ± 6.1 |  | 7.6 ± 3.8 |  |
| *P*_20-40_ |  | 7 | 2.9 ± 2.0 |  | 9.1 ± 6.3 |  | 5.4 ± 3.9 |  |
| *P*_40-60_ |  | 9 | 4.3 ± 3.5 |  | 6.4 ± 5.8 |  | 1.9 ± 3.2 |  |
| *P*_60-80_ |  | 16 | 5.3 ± 3.0 |  | 14.4 ± 10.9 |  | 9.3 ± 10.0 |  |
| *>P*_80_ |  | 9 | 3.9 ± 2.6 |  | 14.0 ± 10.7 |  | 9.0 ± 6.7 |  |
| Cnm | | |  |  |  |  |  |  |
| <*P*_20_ |  | 2 | 4.0 ± 1.4 |  | 7.0 ± 2.8 |  | 2.6 ± 3.7 |  |
| *P*_20-40_ |  | 5 | 5.0 ± 3.6 |  | 9.0 ± 5.7 |  | 4.8 ± 4.2 |  |
| *P*_40-60_ |  | 7 | 3.6 ± 2.6 |  | 7.0 ± 6.4 |  | 3.2 ± 3.9 |  |
| *P*_60-80_ |  | 6 | 5.0 ± 2.3 |  | 17.2 ± 14.1 |  | 11.1 ± 12.6 |  |
| *>P*_80_ |  | 6 | 4.5 ± 3.0 |  | 9.0 ± 3.8 |  | 6.2 ± 3.1 |  |
| ^a^ Percentiles *(P)* of *S. mutans* quantity (CFU) measured by culture in whole saliva at 12 years of age. | | | | | | | |  |
| ^b^ Caries DeFS (Decayed, enamel included, Filled Surfaces) at 12 and 17 years of age. | | | | | | | |  |
| ^c^ ΔDeFS (5y) = 5-years prospective caries increment from 12 to 17 years of age. | | | | | | | |  |
|  | | | | | | | |  |

| **Table S11**. Influence of *S. mutans* adhesin type and *S. mutans* load on caries experience and development in 452 adolescents from 12 to 17 years of age | | | | | | |
| --- | --- | --- | --- | --- | --- | --- |
| *S. mutans* load*^a^* (CFU) |  | DeFS-12y^b^ |  | DeFS-17y^b^ |  | ΔDeFS-5y^c^ |
|  | n | median (IQR) | n | median  (IQR) |  | median  (IQR) |
| SpaP A | | |  |  |  |  |
| <*P*_20_ | 7 | 2.0 (0.0, 3.0) | 6 | 6.0 (3.0, 14.0) |  | 4.2 (1.9, 8.9) |
| *P*_20-40_ | 21 | 2.0 (1.0, 3.5) | 18 | 4.5 (2.0, 10.0) |  | 2.1 (0.0, 5.9) |
| *P*_40-60_ | 25 | 2.0 (0.0, 5.0) | 21 | 4.0 (1.0, 8.0) |  | 1.2 (0.4, 4.3) |
| *P*_60-80_ | 23 | 4.0 (0.0, 6.0) | 20 | 10.0 (4.3, 14.8) |  | 5.3 (0.5, 8.7) |
| *>P*_80_ | 28 | 3.5 (2.0, 6.0) | 24 | 7.0 (4.0, 13.0) |  | 2.9 (1.7, 9.2) |
| SpaP B | | | |  |  |  |
| <*P*_20_ | 8 | 3.5 (0.3, 7.8) | 4 | 9.0 (4.5, 18.0) |  | 6.4 (0.0, 11.8) |
| *P*_20-40_ | 13 | 2.0 (0.5, 5.0) | 11 | 7.0 (3.0, 14.0) |  | 2.6 (1.1, 7.5) |
| *P*_40-60_ | 16 | 4.5 (2.0, 6.0) | 13 | 8.0 (4.0, 9.5) |  | 3.6 (1.4, 5.5) |
| *P*_60-80_ | 17 | 5.0 (2.0, 7.5) | 13 | 10.0 (6.5, 32.0) |  | 6.9 (2.3, 17.3) |
| *>P*_80_ | 15 | 4.0 (1.0, 7.0) | 13 | 6.0 (3.5, 14.5) |  | 4.1 (1.4, 8.8) |
| B-1 |  |  |  |  |  |  |
| <*P*_20_ | 6 | 1.5 (0.8, 7.8) | 4 | 5.5 (4.3, 10.5) |  | 3.7 (3.2, 8.2) |
| *P*_20-40_ | 5 | 1.0 (0.0, 5.0) | 4 | 5.0 (4.3, 11.8) |  | 4.9 (2.8, 9.3) |
| *P*_40-60_ | 4 | 4.5 (2.3, 6.8) | 3 | 8.0 (-) |  | 3.6 (-) |
| *P*_60-80_ | 11 | 5.0 (4.0, 8.0) | 7 | 9.0 (5.0, 27.0) |  | 4.1 (0.9, 18.1) |
| *>P*_80_ | 2 | 2.0 (-) | 2 | 9.5 (-) |  | 6.5 (-) |
| mixed SpaP A/B/C | | |  |  |  |  |
| <*P*_20_ | 4 | 2.5 (2.0, 6.8) | 4 | 11.5 (6.8, 18.5) |  | 6.9 (4.3, 11.5) |
| *P*_20-40_ | 7 | 4.0 (0.0, 4.0) | 7 | 12.0 (2.0, 13.0) |  | 7.0 (1.7, 8.2) |
| *P*_40-60_ | 9 | 5.0 (0.5, 7.5) | 9 | 8.0 (0.0, 10.0) |  | 1.0 (0.0, 3.1) |
| *P*_60-80_ | 16 | 5.0 (4.0, 7.8) | 16 | 9.5 (7.0, 19.8) |  | 5.2 (3.4, 12.3) |
| *>P*_80_ | 9 | 3.0 (2.0, 6.0) | 9 | 10.0 (4.5, 25.0) |  | 7.9 (3.0, 15.9) |
| Cnm | |  |  |  |  |  |
| <*P*_20_ | 2 | 4.0 (-) | 2 | 7.0 (-) |  | 2.6 (-) |
| *P*_20-40_ | 5 | 4.0 (2.0, 8.5) | 4 | 10.5 (3.0, 13.5) |  | 4.7 (1.1, 8.6) |
| *P*_40-60_ | 7 | 3.0 (1.0, 6.0) | 5 | 7.0 (1.5, 12.5) |  | 2.6 (0.2, 6.5) |
| *P*_60-80_ | 6 | 5.0 (2.8, 7.3) | 5 | 10.0 (5.5, 32.5) |  | 3.2 (1.4, 24.8) |
| *>P*_80_ | 6 | 3.0 (2.8, 7.0) | 4 | 8.0 (6.0, 13.0) |  | 6.2 (3.3, 9.1) |
| ^a^ Percentiles *(P)* of *S. mutans* quantity (CFU) measured by culture in whole saliva at 12 years of age. | | | | | | |
| ^b^ Caries DeFS (Decayed, enamel included, Filled Surfaces) at 12 and 17 years of age. | | | | | | |
| ^c^ ΔDeFS (5y) = 5-years prospective caries increment from 12 to 17 years of age. | | | | | | |

| **Table S12**. Characteristics of *S. mutans* strains used in present study | | | | | | | | | | | | | |  |
| --- | --- | --- | --- | --- | --- | --- | --- | --- | --- | --- | --- | --- | --- | --- |
| Strain no. | SpaP | Cnm/Cbm | Serotype |  | Collagen | DMBT1^b^ | Saliva^b^ |  | 12y | 17y | ΔDeFS |  | Repeat^d^ | T*^e^ |
| 29 | B | Cnm | c |  | No | 13.75 | 38.45 |  | 2 | 6 | 2.88 |  | - | + |
| 56 | A | Cnm | c |  | Yes | 26.82 | 54.29 |  | 4 | 14 | 8.66 |  | 20 | - |
| 139 | B | Cnm | c |  | Yes | 88.01 | 66.36 |  | 10 | - | - |  | 17 | - |
| 200 | A | Cnm | e |  | No | 22.98 | 48.38 |  | 3 | 7 | 3.41 |  | - | + |
| 254 | B-1 | Cnm | c |  | No | 6.14 | 13.26 |  | 3 | - | - |  | - | + |
| 309 | A | Cnm | e |  | Yes | 28.69 | 64.83 |  | 7 | 10 | 3.17 |  | 16 | - |
| 318 | B | Cnm | c |  | Yes | 31.52 | 70.21 |  | 3 | 6 | 2.85 |  | 20 | - |
| 320 | A | Cnm | c |  | Yes | 45.97 | 72.62 |  | 5 | 31 | 26.88 |  | 20 | - |
| 343 | A | Cnm | c |  | No | 10.86 | 31.64 |  | 5 | 5 | 0 |  | - | + |
| 351 | B | Cnm | f |  | Yes | 18.43 | 32.16 |  | 5 | 5 | 0 |  | 18 | - |
| 432 | A | Cnm | c |  | Yes | 37.45 | 55.39 |  | 9 | - | - |  | 20 | - |
| 437 | A | Cnm | e |  | Yes | 23.83 | 52.04 |  | 7 | 8 | 1.13 |  | 5 | - |
|  |  |  |  |  |  |  |  |  |  |  |  |  |  |  |
| 106 | B-1 | Cbm | c |  | No | 8.52 | 31.62 |  | 0 | 5 | 3.72 |  | - | + |
| 220 | C | Cbm | k |  | Yes |  |  |  | 5 | - | - |  | 19 | - |
| 322 | B-1 | Cbm | c |  | Yes |  |  |  | 10 | - | - |  | 19 | - |
| 363 | A | Cbm | k |  | Yes |  |  |  | 3 | 7 | 4.03 |  | 15 | - |
| 422 | B-1 | Cbm | c |  | Yes | 26.73 | 33.54 |  | 7 | - | - |  | 17 | - |
|  |  |  |  |  |  |  |  |  |  |  |  |  |  |  |
| 98 | A | - | c |  | No | 14.55 | 20.22 |  | 0 | 6 | 4.89 |  | - | - |
| 189 | B-1 | - | c |  | No | 19.54 | 16.67 |  | 5 | - | - |  | - | - |
| 449 | C | - | c |  | No | 11.54 | 18.45 |  | 2 | 3 | 1.13 |  | - | - |
|  |  |  |  |  |  |  |  |  |  |  |  |  |  |  |
| Mut 1 | A | - | c |  | No |  |  |  |  |  |  |  |  |  |
| Mut 2 | - | Cnm | c |  | Yes |  |  |  |  |  |  |  |  |  |
| DM^f^ | - | - | c |  | No |  |  |  |  |  |  |  |  |  |
| ^a^ Adhesin typing of *S. mutans* strains using qPCR. | | | | | |  |  |  |  |  |  |  |  |  |
| ^b^ Binding to DMBT1 and saliva was measured in hydroxyapatite adhesin assay described previously [8]. The data show the proportion of bound bacteria out of the total amount of added bacteria (percent adhesion). | | | | | | | | | | | | | | |
| ^c^ Caries levels of the host at baseline; DeFS, Decayed, enamel-included Filled Surfaces. | | | | | | | | | | |  |  |  |  |
| ^d^ Number of repeats in the B-repeat domain of *cnm* or *cbm.* | | | | | | | | | | |  |  |  |  |
| ^e^ Presence of stop codon before or in collagen-binding domain in *cnm* or *cbm.* | | | | | | | | | | |  |  |  |  |
| ^f^ Double mutant with both *spap* and *cnm* gene knocked out. | | | | | | |  |  |  |  |  |  |  |  |

| **Table S13.** Oral hygiene and diet habits in Swedish adolescents with different *S. mutans* infection status, 12 to 17 years of age | | | | | | | | |
| --- | --- | --- | --- | --- | --- | --- | --- | --- |
|  |  | *S. mutans* infection status, 12 to 17 years of age^a^  Frequency % (number n) | | | | | |  |
| Lifestyle^b^ | | S.m neg | Stable dom. | Loss | Gain | Dom to mix | Mix to dom | Stable mix |
| Tooth brushing^c^ | |  |  |  |  |  |  |  |
| 12y | >2 times/day | 1 | 2 (2) | 0 (0) | 2 (1) | 7 (1) | 10 (1) | 0 (0) |
|  | 2 times/day | 89 | 77 (84) | 97 (35) | 85 (51) | 79 (11) | 80 (8) | 70 (0) |
|  | Once/day | 10 | 15 (16) | 3 (1) | 13 (8) | 14 (2) | 10 (1) | 20 (20) |
|  | Irregularly | 1 | 6 (7) | 0 (0) | 0 (0) | 0 (0) | 0 (0) | 10 (10) |
|  |  |  |  |  |  |  |  |  |
| 17y | >2 times/day | 5 | 2 | 0 | 2 | 7 | 0 | 0 |
|  | 2 times/day | 82 | 72 | 86 | 70 | 71 | 90 | 90 |
|  | Once/day | 9 | 23 | 11 | 16 | 21 | 10 | 0 |
|  | Irregularly | 4 | 3 | 3 | 12 | 0 | 0 | 10 |
|  |  |  |  |  |  |  |  |  |
| Intake of sweets | |  |  |  |  |  |  |  |
| 12y | Never | 0 | 1 | 0 | 0 | 0 | 0 | 0 |
|  | Once a month | 1 | 1 | 0 | 2 | 7 | 0 | 0 |
|  | Once a week | 40 | 39 | 31 | 54 | 29 | 50 | 33 |
|  | Several times a week | 49 | 54 | 58 | 43 | 57 | 40 | 56 |
|  | Once a day | 8 | 5 | 11 | 2 | 7 | 10 | 11 |
|  | Several times a day | 1 | 1 | 0 | 0 | 0 | 0 | 0 |
|  |  |  |  |  |  |  |  |  |
| 17y | Never | 1 | 1 | 0 | 2 | 0 | 0 | 0 |
|  | Once a month | 5 | 5 | 9 | 11 | 0 | 0 | 0 |
|  | Once a week | 29 | 28 | 43 | 36 | 7 | 20 | 40 |
|  | Several times a week | 57 | 54 | 46 | 46 | 64 | 70 | 50 |
|  | Once a day | 6 | 5 | 3 | 0 | 21 | 10 | 10 |
|  | Several times a day | 1 | 1 | 0 | 5 | 7 | 0 | 0 |
|  |  |  |  |  |  |  |  |  |
| Intake of sugary drinks | |  |  |  |  |  |  |  |
| 12y | Never | 2 | 3 | 3 | 7 | 0 | 0 | 0 |
|  | Once a month | 4 | 6 | 6 | 8 | 7 | 0 | 0 |
|  | Once a week | 32 | 28 | 33 | 36 | 21 | 40 | 50 |
|  | Several times a week | 49 | 56 | 47 | 44 | 64 | 40 | 30 |
|  | Once a day | 11 | 5 | 8 | 5 | 7 | 10 | 20 |
|  | Several times a day | 2 | 3 | 3 | 2 | 0 | 10 | 0 |
|  |  |  |  |  |  |  |  |  |
| 17y | Never | 3 | 9 | 6 | 10 | 0 | 10 | 0 |
|  | Once a month | 7 | 6 | 14 | 11 | 0 | 0 | 0 |
|  | Once a week | 27 | 29 | 26 | 30 | 36 | 40 | 30 |
|  | Several times a week | 48 | 42 | 46 | 43 | 57 | 40 | 50 |
|  | Once a day | 12 | 9 | 6 | 3 | 0 | 0 | 20 |
|  | Several times a day | 4 | 4 | 3 | 3 | 7 | 10 | 0 |
|  | |  |  |  |  |  |  |  |
| Use of extra fluoride | |  |  |  |  |  |  |  |
| 12y | Yes | 21 | 23 | 31 | 25 | 0 | 40 | 30 |
|  | No | 79 | 77 | 69 | 75 | 100 | 60 | 70 |
|  |  |  |  |  |  |  |  |  |
| 17y | Yes | 16 | 25 | 26 | 33 | 7 | 50 | 40 |
|  | No | 83 | 75 | 74 | 67 | 93 | 50 | 60 |
|  |  |  |  |  |  |  |  |  |
| Total (N) | | 144 | 109 | 36 | 60 | 14 | 10 | 10 |
| ^a^ *S. mutans* infection status measured by qPCR in whole saliva from 388 adolescents at 12 and 17 years of age. | | | | | | | | |
| ^b^ Lifestyle data collected by questionnaire at 12 and 17 years of age. | | | | | | | | |
| ^c^ Significant differences (p = 0.0083, chi-square test) in tooth brushing frequency among different *S. mutans* infection status from 12 to 17 years of age. | | | | | | | | |

| **Table S14**. Virulence-related properties of wild-type isolates of the *S. mutans* adhesion phenotypes | | | | | | | | | | | | | | | | | | | |
| --- | --- | --- | --- | --- | --- | --- | --- | --- | --- | --- | --- | --- | --- | --- | --- | --- | --- | --- | --- |
|  |  |  | Adhesion to^b^ | | | | | | | |  | Binding to^c^ | |  | Biofilm formation | |  | Acid tolerance | |
|  |  |  | collagen type | | | | | |  | DMBT1 |  | buccal tissue | |  | surface coverage (%) | high-density area |  | (% viability)^d^ | |
| Strain/ | Adhesin^a^ |  | I | II | III | IV | V | VI |  |  |  | epith | sub. |  |  |  |  | no adapt | adapt^e^ |
| Protein |  |  |  |  |  |  |  |  |  |  |  |  | epith |  |  |  |  |  |  |
| 98 | SpaP A |  | - | 1 | 1 | - | 2 | - |  | 2 |  | - | - |  | 1.6 | No |  | 44 | 80 |
| 189 | SpaP B |  | 1 | - | 1 | - | 1 | - |  | 2 |  | - | - |  | 2.5 | No |  | 42 | 90 |
| 449 | SpaP C |  | - | - | 1 | - | 2 | - |  | 2 |  | - | - |  | 2.1 | No |  | 2 | 82 |
| 56 | Cnm (A) |  | 3 | 4 | 3 | 5 | 4 | - |  | 2 |  | - | 3 |  | 9.3 | Yes |  | 1 | 61 |
| 422 | Cbm (B) |  | 3 | 5 | 5 | 5 | 5 | - |  | 2 |  | - | 4 |  | 2.3 | No |  | 11 | 63 |
| Recombinant proteins | | | | |  |  |  |  |  |  |  |  |  |  |  |  |  |  |  |
| rCnm |  |  | 5 | 3 | 1 | 2 | 2 | 1 |  | 2 |  |  |  |  |  |  |  |  |  |
| rCbm |  |  | 4 | 1 | 1 | 1 | 2 | - |  | 2 |  |  |  |  |  |  |  |  |  |
| ^a^ Adhesion typing of *S. mutans* strains using qPCR. | | | | | | | | | | |  |  |  |  |  |  |  |  |  |
| ^b^ *S. mutans* adhesion to collagen and DMBT1 was measured in a dot blot assay. The scores (1-5) mark avidity of binding, - marks no binding. | | | | | | | | | | | | | | | | | | | |
| ^c^ *In situ* binding of FITC-labeled *S. mutans* strains to buccal tissue sections | | | | | | | | | | | | | | | | | | | |
| ^d^ Acid tolerance was measured by counting viable cells under confocal microscopy after exposure to pH 3.5. | | | | | | | | | | | | | | | | | | | |
| ^e^ *S. mutans* cells were adapted at sublethal pH value (5.5) before exposure to a lower pH (3.5). | | | | | | | | | | | | | | | | |  |  |  |

| **Table S15**. Prevalence of *S. mutans* adhesion types during adolescence and puberty | | | | | | | |
| --- | --- | --- | --- | --- | --- | --- | --- |
| Type^a^ |  | Prevalence^b^ | | | | | |
|  |  | 12 years of age  (N = 452) | |  | 17 years of age  (N = 390) | |  |
|  |  | % | n |  | % | n | P*^c^* |
| *S. mutans* |  | 48 | 217 |  | 55 | 216 | 0.027 |
|  |  |  |  |  |  |  |  |
| SpaP A |  | 24 | 111 |  | 29 | 113 | 0.14 |
| SpaP B |  | 16 | 71 |  | 10 | 39 | 0.018 |
| SpaP C |  | 4 | 19 |  | 6 | 24 | 0.21 |
|  |  |  |  |  |  |  |  |
| Mixed SpaP ABC |  | 6 | 27 |  | 9 | 33 | 0.16 |
|  |  |  |  |  |  |  |  |
| Cnm |  | 6 | 26 |  | 5 | 19 | 0.65 |
| Cbm |  | 2 | 7 |  | 2 | 6 | 1.0 |
| ^a^ *S. mutans* types measured in whole saliva by qPCR in adolescents at 12 and 17 years of age. | | | | | | | |
| ^b^ Prevalence in adolescents at 12 and 17 years of age; data were collected from 452 children at 12 years of age and from 390 adolescents at 17 years of age. | | | | | | | |
| ^c^ P value from Fisher’s exact test (2-sided). | | | | | | | |

**Fig. S1.** Numbers of *S. mutans* adhesion types and modes measured by qPCR at 12 (a) and 17 (b) years of age (Mann-Whitney U test). Box-and-whisker plot with median and lower (Q1) and upper (Q3) quartiles and whiskers representing non-outlier minimum and maximum values; individual outliers (circles) are 1.5× interquartile range outside of Q1 and Q3.

The proportion of mixed SpaP ABC out of total streptococci was also increased significantly compared to dominant SpaP A/B/C (data not shown).

**Fig. S2. a.** Identification of Cnm, Cbm proteins in cell wall extracts from Cnm and Cbm *S. mutans* clinical isolates using western blot with Cnm- and Cbm-specific antisera, showing one distinct Cnm/Cbm protein band that migrated slightly lower than 150 kDa. Variation in both band size and number can be noted among the strains. Results are from three separate membranes. **b.** Detection of glycoprotein in cell wall extracts from 12 Cnm and 5 Cbm strains. The positive control was parotid saliva. Strains with a stop codon in the collagen-binding domain (strain 29, 200, 245–343 and 106), as well as additional 2 Cnm-positive strains (351 and 437) with shorter B-repeat domains, had no glycan detected. Results are from three separate membranes. The figure is a duplicate of Fig. 4c.

**Commentary results**

**Protein patterns in *cnm-* and *cbm*-positive strains**

To explore the protein pattern of *cnm*-and *cbm*-positive strains, we analyzed cell wall extracts from 12 *cnm-* and 5 *cbm*-positive strains of known *cnm* gene sequences using Cnm- and Cbm-specific antisera (generated by using recombinant Cnm, Cbm protein; Agrisera, Sweden).

The Cnm-specific antisera detected one distinct Cnm band at approximately 150 kDa in the eight *cnm*-positive strains with intact *cnm* genes. Three of the four strains with translational stop codons in the collagen-binding domain in their *cnm* genes had an apparent lack of expression of Cnm-related protein bands. Strain 29, with an early stop codon before the collagen-binding domain, yielded two bands of size 75 and 90 kDa, respectively, and no 150-kDa band. For strains 139, 351, and 437, there were Cnm-related protein bands of slightly lower molecular weight in accordance with their smaller gene sizes because of their shorter repeats domain (Fig. S2a). Moreover, the intensity of antisera staining of the Cnm-related protein bands was inversed in some of the strains, indicating additional factors regulating expression of Cnm-related proteins.

A single Cbm protein band of 120 kDa was detected for four *cbm*-positive strains by the antisera, and the Cbm size banding pattern matched the variation in *cbm* gene size, with strain 363 showing a smaller, faster-migrating Cbm protein band. For strain 106, with a translational stop codon, there was no corresponding Cbm band (Fig. S2a).

**Glycosylation patterns in *cnm*- and *cbm*-positive strains**

The glycosylation pattern of the *cnm*-and *cbm*-positive strains was determined by glycan staining (Fig. 4 and S1B). Several glycosylated protein bands were detected for the majority of strains expressing Cnm or Cbm but not in the strains with stop codons and not expressing Cnm- and Cbm-related proteins (Figs. 4 and S2b). However, strains 351 and 437, expressing Cnm but possessing shorter B-repeat domains, also lacked glycosylation (Figs. 4 and S2b).

**
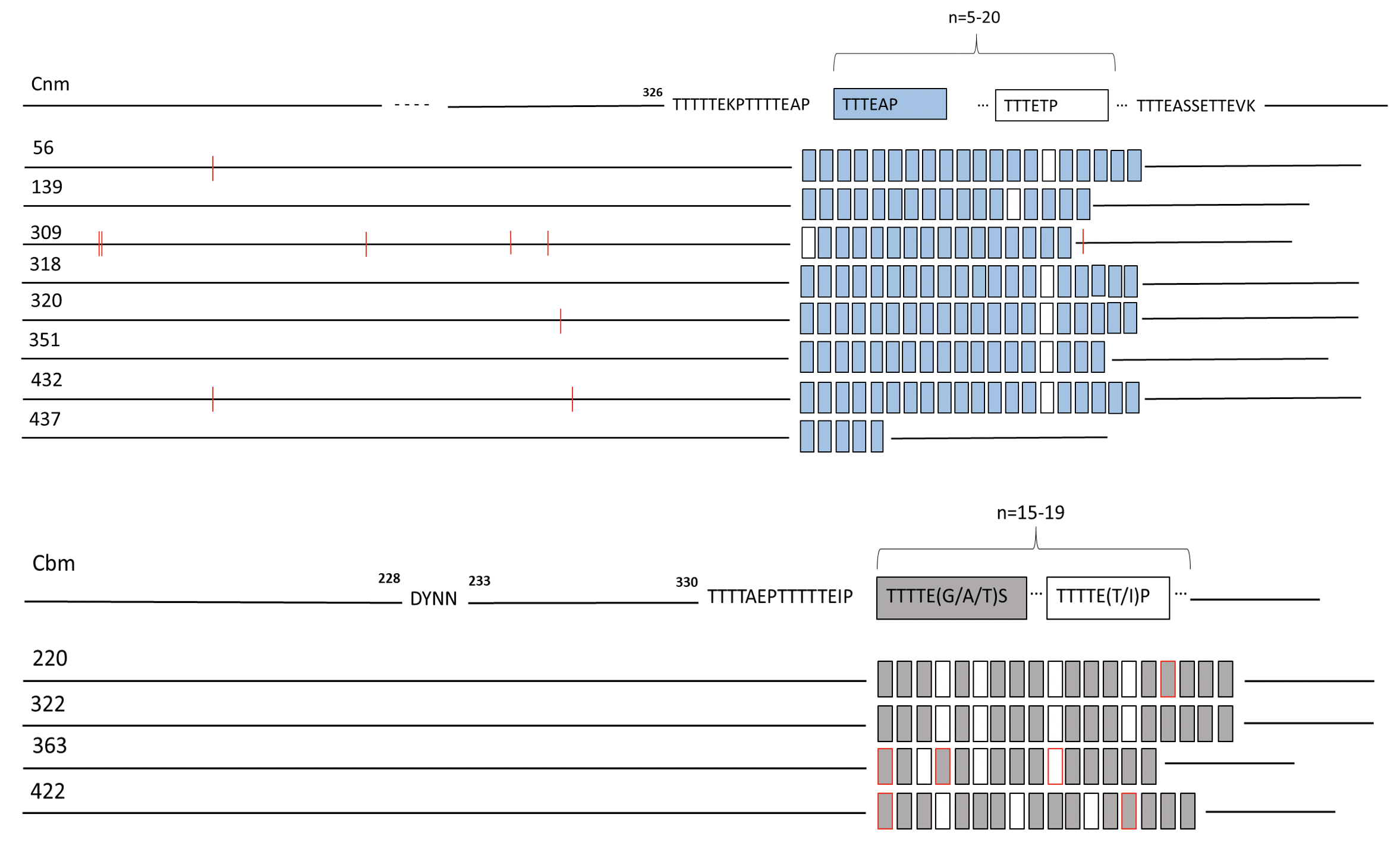
**

**Fig. S3.** Variation in Cnm and Cbm protein structures, focusing on variation in numbers and sequence structures of the B-repeat motifs for O-glycosylation. The deduced Cnm and Cbm protein structures were highly homologous, particularly in the collagen-binding domain, with consensus threonine-rich B-repeat motifs TTTE(A/T)P for Cnm and TTTTE(G/A/T)S or TTTTE(T/I)P for Cbm. The major B-repeat motifs also varied within Cnm and Cbm isolates. The collagen-binding domain in Cbm had a unique DYNN insertion (aa 229–232).

**Fig. S4. a.** Range of *S. mutans* SpaP B in infected children at 12 years of age (bold line) and changes at 17 years of age (grey line). Quantitative numbers of *S. mutans* SpaP B are given as the qPCR response (pg DNA) in each child at 12 (bold line) and 17 (grey line) years of age. The cut-off level (dotted line) marks infected and non-infected children at 12 years of age (- / +). **b.** Fluctuation of *S. mutans* amounts in saliva at repeated measures in 71 volunteers. Three saliva samples collected from 71 individuals at three different time points (once a month) as measured by qPCR for *S. mutans* quantity.
